# Evaluation of three decades of the burden of low back pain in China before COVID-19: Estimates from the Global Burden of Disease Database 2019

**DOI:** 10.7189/jogh.14.04006

**Published:** 2024-03-15

**Authors:** Shuai Xu, Jinlei Qi, Chenjun Liu, Weiwei Xia, Zhenbo Wang, Kexin Li, Maigeng Zhou, Haiying Liu

**Affiliations:** 1 Department of Spinal Surgery, Peking University People’s Hospital, Peking University, Beijing, China; 2 National Center for Chronic and Noncommunicable Disease Control and Prevention, Chinese Center for Disease Control and Prevention, Beijing, China; 3 Institute of Geographical Sciences and Natural Resources Research, Chinese Academy of Sciences, Beijing, China

## Abstract

**Background:**

Low back pain (LBP) is reported as an urgent public-health concern globally because it occurs in all age groups and is now the leading cause of disability, with health systems unable to cope with this burden. We present China’s burden of LBP by estimating its prevalence and years lived with disability (YLDs) from 1990 to 2019.

**Methods:**

We obtained the data relating to LBP from the Global Burden of Disease Database (GBD) 2019. Then we calculated years lived with disability caused by LBP by multiplying the prevalence of LBP sequelae by their corresponding disability weights. We performed an analysis of the age-, sex-, and province-specific prevalence and YLDs of 33 provinces/regions in China, as well as their relationship with the sociodemographic index (SDI).

**Results:**

China has the largest numbers of people with LBP (91.3 million) and YLDs (8.6 million) globally, and LBP is the leading cause of YLDs. The age-standardised prevalence was 7.25% in 1990, and this decreased to 5.13% in 2019. The age-standardised YLD rate was 579/100 000 in 2019, having decreased by 28.97%. Both measurements increased with age, being higher in women and varying across the 33 provinces/regions. For the 5-to-14-year age group, the prevalence (4.50%) and YLD rate (4.51%) increased in 2019 from 1990 (3.21% and 3.21%, respectively) when compared to the elderly group. Age-standardised YLD rates experienced decreases with increasing SDI, while there was an increasing tendency as SDI increased further; the changes for women were more obvious.

**Conclusions:**

Over the three decades considered, China has continued to have the largest number of people with LBP in the world, even though the age-standardised prevalence has decreased. YLDs were found to decrease as SDI increased, but they subsequently increased again. LBP still presents a burden, particularly for children and postmenopausal women.

Low back pain (LBP) is a symptom characterised by a range of biophysical, psychological, and social dimensions [1–3]. It is an extremely common symptom experienced by all ages, and it is the most common cause of years lived with disability (YLDs) globally [4]. In high-income countries, the prevalent health care approaches to LBP contribute to the overall burden and cost, and social support systems are negatively affected by LBP in low- and middle-income countries. Thus, LBP is an urgent global public health concern [3,5,6].

As a developing and middle-income country, China has an increasing number of people with LBP, and it already has the largest number of any country worldwide; however, the 30-year epidemiological characteristics of LBP in China are still unclear [7,8]. Because previous epidemiological studies of LBP have used small sample sizes and limited localities, the Global Burden of Disease, Injuries, and Risk Factors Study 2019 (GBD 2019) [4,9,10] included a comprehensive assessment of the epidemiological characteristics of the prevalence and YLDs caused by LBP in China. The GBD 2019 included an updated DisMod-MR tool, the construction of a sociodemographic index (SDI), and further research to establish disability weights (DWs) [4].

Therefore, using GBD 2019, this study sought to examine China’s burden of LBP by estimating its spatial, temporal, and population point prevalence and YLDs, their 30-year changes, and the relationship between YLDs and SDI. The results are expected to lead to more attention being paid to the prevention and treatment of LBP in China.

## METHODS

### Data source

We obtained all data and analysis from the updated GBD 2019 (https://www.healthdata.org/gbd). In GBD 2019, we can use the large number of regions and countries to provide sufficient population-based prevalence and burden estimates of LBP, and its quantification of LBP is thus more accurate than previous GBD studies. It provides comprehensive assessments of age- and sex-specific mortality and years of life lost for 282 causes, the prevalence and YLDs for 87 risk factors, and 369 diseases and injuries in 204 countries and territories (all World Health Organization member states) for the three decades from 1990 to 2019[4,9–11].

The present study focused mainly on the prevalence of LBP and the numbers of LBP-related YLDs in China. First, we obtained the incidence of LBP for all countries (https://vizhub.healthdata.org/gbd-results/) and then identified China’s rank [4]. We also pooled data from each of 33 province-level regions, including 31 mainland provinces and the Hong Kong and Macao special administrative regions; data from Taiwan is not included. We also acquired the 30-year SDI from all 33 provinces/regions.

Generally, annual updates are conducted to the GBD to incorporate new causes and data (including published literature, surveillance data, survey data, hospital and clinical data, and other types of data) relating to LBP and to improve demographic and statistical methods. In this database, different methods can be applied to use the available data and to measure specific epidemiological patterns of LBP. Then we can use data relating to the severity and occurrence of LBP to establish the proportions of cases experiencing each sequela. For China, we extracted all relevant open data on LBP from available databases and pooled into the GBD. The China Health and Retirement Longitudinal Study (CHARLS) plays an important role among these data sources. CHARLS aims to analyse the issue of population aging (over 45) in China and promote interdisciplinary research on aging issues. By 2021, the sample had covered a total of 19 000 respondents from 12 400 households [12].

The scholars applied a standard Bayesian meta-regression tool (version DisMod-MR 2.1) in GBD 2019 to handle multi-source data sets. This is used to address the challenges involved in estimating the point-prevalence and YLD outcomes, increasing the computational speed and allowing consistent computations between all disease parameters at the country level. More detailed descriptions of this modelling strategy for GBD 2019 have been published elsewhere [4,13].

### Prevalence and YLDs estimates

The experts defined low back pain as back pain that lasts for at least one day (with/without one or both lower limbs). The definition of the ‘low back’ region is the posterior area between the lower margin of the 12th ribs and the lower gluteal folds [1,7,14]. The DWs represent the magnitudes of health loss associated with LBP. Those are measured on a scale from zero to one, with zero representing a state of full health and one representing a state equivalent to death. The DWs used in GBD 2019 have been described previously [15,16].

There are a total of eight sequelae to describe the different levels of LBP severity and its associated functional loss [17]: (I) severe acute LBP without leg pain; (II) severe chronic LBP without leg pain; (III) mild acute LBP without leg pain; (IV) mild chronic LBP without leg pain; (V) severe acute LBP with leg pain; (VI) severe chronic LBP with leg pain; (VII) mild acute LBP with leg pain; and (VIII) mild chronic LBP with leg pain. Since there is no mortality from LBP, the YLDs and disability-adjusted life years (DALYs) values are the same. Thus, we have only used YLD values in this paper, which are calculated using: (1) *YLD_total_ = YLD_sequela1_ + YLD_sequela2_ + ·  ·  · + YLD_sequela8_*;

(2) *YLD_sequela_ = Prevalence_sequela_ × DW_sequela_*.

### Sociodemographic index

The sociodemographic index was first constructed in GBD 2015 [18,19]. This is the geometric mean of zero-to-one indices of: total fertility rate under the age of 25 (TFU25); mean education for those aged 15 and older (EDU15+); and lag-distributed income (LDI) per capita. The experts calculated uncertainty interval (UI) from the standard errors generated from the input data, and calculated the uncertainty from all steps of data manipulations. The 95% UI ranges from the 2.5 to 97.5 centile values [20].

## RESULTS

### Numbers of those with LBP in China

Globally, the estimated prevalence of LBP was 568.4 million (95% UI = 505.0–640.6 million) in 2019, with an increase of 182.4 million since 1990. Over the three decades considered, LBP has always been ranked as the leading cause of YLDs in the world. According to GBD 2019, China had the largest population with LBP both in 1990 (75.3 million, 95% UI = 66.0–85.1 million) and 2019 (91.3 million, 95% UI = 80.5–104.1 million), showing an increase of 16.0 million. In addition, China also had the largest population base of YLDs caused by LBP (10.3 million) in 2019, an increase of 1.78 million. We presented the data in Table S1–S2 and Figure S1 in the **Online Supplementary Document**.

### Prevalence of LBP in China

**Figure 1:**
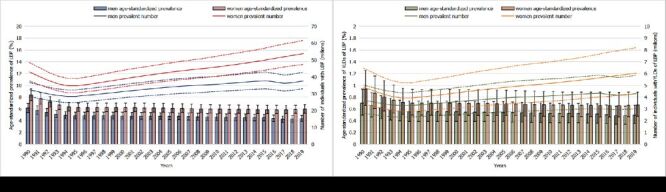
Thirty-year trends in prevalence and YLDs caused by LBP in China. **Panel A.** Estimated prevalence and age-standardised point prevalence of people with LBP from 1990 to 2019 by gender. **Panel B.** Estimated prevalence and age-standardised point prevalence of YLDs caused by LBP from 1990 to 2019 by gender. The dotted lines indicate the upper uncertainty interval (UUI) and lower uncertainty interval (LUI). LBP – low back pain, YLDs – years lived with disability

**Figure 2:**
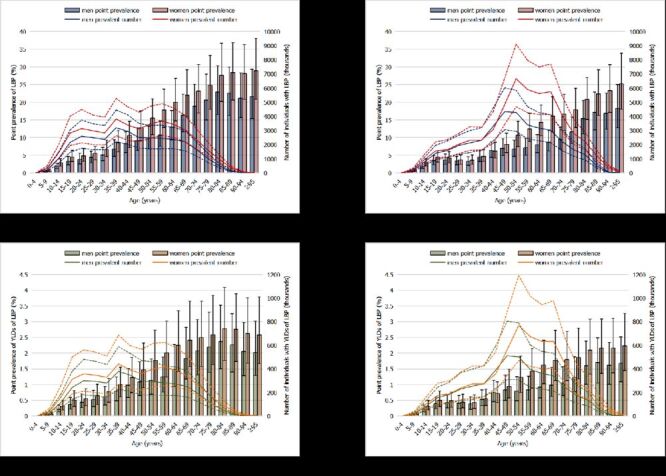
Prevalence and YLDs caused by LBP for different age groups in China. **Panel A.** Estimated prevalence and point prevalence rate of LBP in 1990 by gender. **Panel B.** Estimated prevalence and point prevalence rate of LBP in 2019 by gender. **Panel C.** Estimated prevalence and point prevalence rate of YLDs caused by LBP in 1990 by gender. **Panel D.** Estimated prevalence and point prevalence rate of YLDs caused by LBP in 2019 by gender. The dotted lines indicate UUI and LUI. LBP – low back pain, LUI – lower uncertainty interval, UUI – upper uncertainty interval, YLDs – years lived with disability

In China, the age-standardised prevalence of LBP was 7.25% (95% UI = 6.39–8.17%) in 1990, and this decreased to 5.13% (95% UI = 4.55–5.79%) in 2019. The prevalence for women was higher than that for men throughout the period from 1990 to 2019 (**Figure 1**, panel A). Overall, in 1990, the prevalence of LBP was concentrated in the age range 35 to 50 years, peaking between 35 and 39 years; in 2019, this moved to 45 to 70 years with a peak between 50 and 54 years. The point prevalence increased from the lowest level in the category zero to four years and peaked at >95 years for both 1990 and 2019, with similar trends for men and women, excluding for men in 1990. The prevalence in women increased faster than that in men, and a sex disparity occurred in the 30-to-34 age group in 1990 but in the 45-to-49 age group in 2019 (**Figure 2**, panels A and B).

**Figure 3:**
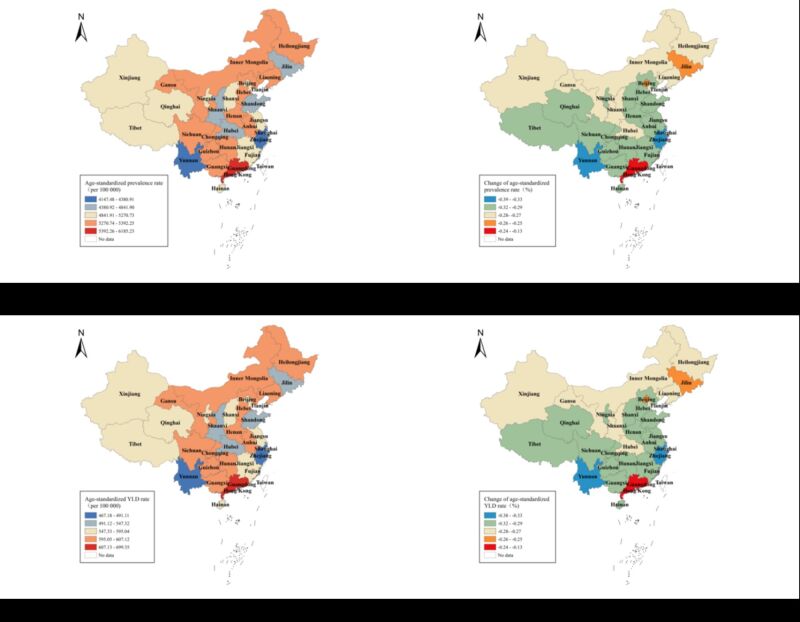
Maps of the age-standardised prevalence and LBP-related YLDs in China. **Panel A.** Age-standardised point prevalence of LBP in 2019. **Panel B.** Age-standardised point prevalence of LBP with 30-year change (1990–2019). **Panel C.** Age-standardised YLDs caused by LBP in 2019. **Panel D.** Age-standardised YLDs caused by LBP with 30-year change (1990–2019). LBP – low back pain, YLDs – years lived with disability.)

In the 33 provinces/regions in China, Guizhou had the highest age-standardised point prevalence of LBP at 7.6% (95% UI = 6.8–8.5%) in 1990, while Hong Kong and Guangdong were always in the top-two places after 1994. Shanghai had the lowest age-standardised prevalence of 6.5% (95% UI = 5.5–7.4%) in 1990, but Zhejiang had the lowest age-standardised prevalence in 2019, with the largest decrease, by 38.6% (95% UI = 34.6–43.0%). The age-standardised prevalence values in most provinces/regions (25/33) were ˃7.0% in 1990, while most (32/33) were less than 6.0% in 2019 (**Figure 3**, panels A and B). We presented the data in Table S3 in the **Online Supplementary Document**.

### Prevalence of YLDs caused by LBP in China

The age-standardised YLD rate was 0.58% (95% UI = 0.41–0.78%) in 2019, a decrease of 28.97% (95% UI = 27.0–30.8%) since 1990, and the YLD rate was higher for women than for men (**Figure 1**, panel B). With similar trends to the prevalence of LBP, the number of YLDs peaked in the 35-to-39-year age range in 1990, and this moved to the 50-to-54-year age range in 2019. In 1990, the YLD rate increased with age from zero to four years and peaked at 80 to 84 years, while the YLD rate peaked at the age of ˃95 years in 2019. The sex disparity, with rates higher in women, increased in the 30-to-34-year age range in 1990, while it moved to the 50-to-54-year age range in 2019 (**Figure 2**, panels C and D).

Hong Kong and Guangdong were the two regions with the highest age-standardised YLDs (0.70 and 0.65%, respectively) and Zhejiang was the province with the lowest YLDs (0.47%) in 2019. Zhejiang and Shanghai had the greatest decrease since 1990. Low back pain ranked as the leading cause of YLDs in all 33 provinces/regions in 1990, but only in 25 of the 33 provinces/regions in 2019 (**Figure 3**, C and D). Overall, LBP was always the leading cause of YLDs in China (**Table 1**). We presented the data in Table S4 and Figure S2 in the **Online Supplementary Document**.

**Table 1:** Proportions of all-cause age-standardised YLDs relating to LBP by region (1990 and 2019)

Province-level administrative units	YLDs		Prevalence	
	Proportion of all causes (ranking)* in 1990	Proportion of all causes (ranking)* in 2019	**Proportion of all causes (ranking) in 1990**	**Proportion of all causes (ranking) in 2019**
Anhui	8.72% (1)	7.16% (1)	6.80%	7.13%
Beijing	8.49% (1)	6.57% (1)	7.24%	6.76%
Chongqing	8.87% (1)	6.82% (1)	7.44%	7.55%
Fujian	8.63% (1)	7.01% (1)	6.37%	6.57%
Gansu	8.30% (1)	6.86% (1)	6.47%	6.82%
Guangdong	8.03% (1)	7.35% (1)	6.27%	6.94%
Guangxi	8.60% (1)	6.96% (1)	6.51%	6.41%
Guizhou	8.13% (1)	6.81% (1)	6.40%	6.54%
Hainan	8.34% (1)	6.76% (1)	6.41%	6.32%
Hebei	9.13% (1)	6.93% (1)	7.05%	6.81%
Heilongjiang	8.48% (1)	7.04% (2)	6.49%	7.65%
Henan	9.06% (1)	7.08% (1)	6.91%	6.66%
Hong Kong	9.14% (1)	8.26% (1)	7.88%	9.33%
Hubei	7.44% (1)	6.19% (2)	5.82%	6.40%
Hunan	8.47% (1)	6.88% (1)	6.89%	7.00%
Inner Mongolia	8.62% (1)	7.03% (1)	6.36%	7.13%
Jiangsu	8.84% (1)	6.86% (1)	7.35%	7.36%
Jiangxi	8.41% (1)	7.02% (1)	6.44%	6.42%
Jilin	7.70% (1)	6.69% (2)	5.75%	6.93%
Liaoning	9.15% (1)	7.09% (1)	7.18%	7.81%
Macao	8.78% (1)	7.02% (1)	7.10%	6.80%
Ningxia	8.57% (1)	6.92% (1)	5.90%	6.19%
Qinghai	8.78% (1)	7.05% (1)	6.04%	6.18%
Shaanxi	7.79% (1)	6.40% (2)	5.85%	6.10%
Shandong	7.97% (1)	6.19% (2)	6.23%	6.14%
Shanghai	7.68% (1)	5.31% (3)	7.41%	5.69%
Shanxi	8.70% (1)	7.08% (1)	6.79%	6.74%
Sichuan	8.84% (1)	6.78% (1)	7.21%	7.35%
Tianjin	8.98% (1)	6.90% (1)	7.23%	6.76%
Tibet	7.97% (1)	6.57% (2)	6.15%	5.33%
Xinjiang	8.21% (1)	6.73% (1)	6.03%	6.07%
Yunnan	7.11% (1)	5.56% (2)	5.51%	5.21%
Zhejiang	7.81% (1)	5.38% (4)	6.62%	5.56%
China	8.43% (1)	6.72% (1)	6.64%	6.70%

LBP – low back pain, YLDs – years lived with disability

*Ranking of LBP among all causes, according to the burden of age-standardised YLDs in 1990 and 2019.

### Prevalence and YLD rate based on age stratification

**Figure 4:**
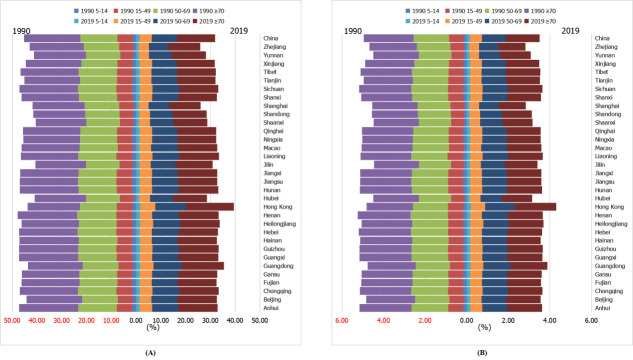
Point prevalence and LBP-related YLDs in the various age groups (5–14, 15–49, 50–69, and ≥70) in different provinces/regions in China. **Panel A.** Point prevalence of LBP in 1990 and 2019. **Panel B.** LBP-related YLDs in 1990 and 2019. LBP – low back pain, YLDs – years lived with disability

Four age categories, 5 to 14, 15 to 49, 50 to 69, and ≥70 years, were used for age stratification. In 1990, Jiangxi, Hebei, Henan, and Hainan had the highest LBP prevalence in all four age groups, while in 2019, Hong Kong had the highest prevalence for all age groups. The proportions of LBP prevalence in the 5-to-14-year (4.50%) and 15-to-49-year (15.1%) age groups increased for all provinces/regions over the 30-year period (3.21% and 13.3%, respectively) (**Figure 4**, panel A). We presented the data in Table S5 in the **Online Supplementary Document**. Similarly, Hong Kong was the region with highest YLD rate for all age groups in 2019. The proportions of the YLD rate also had 30-year increases in the 5-to-14-year (4.51%) and 15-to-49-year (16.1%) age groups in 2019 (**Figure 4**, panel B). We presented the data in Table S6 in the **Online Supplementary Document**.

### SDI and the age-standardised YLDs

**Figure 5:**
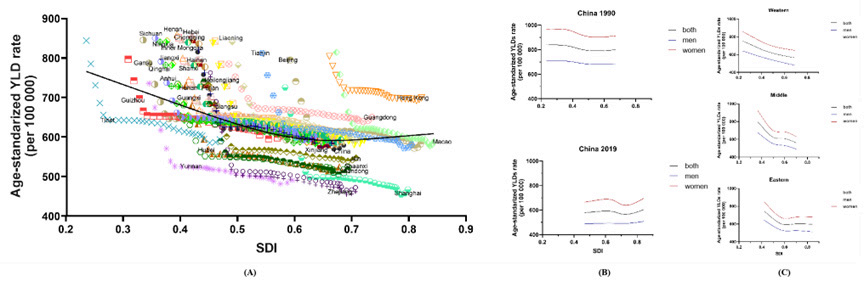
Relationship between YLDs and SDI in China. **Panel A.** Co-evaluation of age-standardised YLDs caused by LBP as functions of SDI in China and for 33 provinces/regions, 1990–2019. **Panel B.** Respective evaluation of relationship between YLDs and SDI in 1990 and in 2019. **Panel C.** Relationship between YLDs and SDI in the eastern, central, and western regions of China, 1990–2019. LBP – low back pain, SDI – socio-demographic index, YLDs – years lived with disability

**Figure 5** shows the age-standardised LBP-related YLD rates as functions of SDI for all 33 provinces/regions from 1990 to 2019. In general, age-standardised LBP-related YLD rates in all provinces/regions experienced decreases with increasing SDI. However, notably, the curve had a climbing tendency with SDI values beyond 0.65 (**Figure 5**, panel A). In 1990, age-standardised YLD rates decreased with SDI, and they had a high overall level. In 2019, it slightly increased in a lower level with a forward-moved SDI range (**Figure 5**, panel B).

We divided the provinces/regions into the eastern, central, and western regions of China, and each area showed a decreasing trend. However, there were particular features in each region: western China presented an almost straight line, and central China outlined an ‘S’ shape. The trend of eastern China remained stable for SDI values above 0.6, and this approximated the overall pattern of China as a whole. In addition, all outcomes showed that the rate change for women was more obvious than that for men (**Figure 5**, panel C). We presented the data in Table S7 in the **Online Supplementary Document**.

## DISCUSSION

This study examined the changes in LBP data in China from 1990 to 2019 using GBD 2019 data. Overall, the age-standardised prevalence of LBP decreased over this 30-year period, but the number of LBP cases and YLDs have increased, both in China and worldwide. It has been reported that LBP has long been the leading cause of YLDs worldwide [9], and this is also the case in China. The population with LBP and YLDs is thus still a notable problem for in China.

From this study, it is concluded that the age-standardised prevalence of LBP is higher in women. Potential explanations of sex differences include comprehensive interactions of different factors [1,21,22]. Physically, women are affected by more painful conditions of the musculoskeletal system and paraspinal muscle, especially the psoas major and erector spinae [23]. Psychologically, it has been suggested that women have higher sensitivity to pain and greater willingness to report painful complaints [24]. Biologically, the menstrual cycle, hormonal fluctuations, and pregnancy may also have an impact on the musculoskeletal and neurological systems [25]. In addition, the prevalence of osteoporosis and micro-fractures in the lumbar spine is significantly higher in older women [26]. Interestingly, there is a higher prevalence in men in countries distributed in Eastern sub-Saharan Africa and North Africa, which may be more intensively influenced by psychological and sociocultural factors [27]. We presented the data in Table S8–S9 in the **Online Supplementary Document**.

Hong Kong and Guangdong were the two provinces/regions with the highest age-standardised prevalence of LBP in most periods. It has been reported that physically challenging work may be a risk factor for overloading of the spine, which may contribute to LBP caused by the deterioration of paraspinal muscle, disc herniation, and lumbar stenosis [28,29]. Hong Kong and Guangdong are provinces/regions with large levels of migrant labour, with lights turned on all night and high work pressure. Therefore, identification of the aetiology and causes of LBP is critical since this wide-ranging complaint, especially nonspecific and chronic LBP, can be classified into many types [30,31]. It must be acknowledged that LBP can arise and be aggravated by serious psychological problems, excessive earning pressure, as well as limited exercise time [32]. Clearly, Hong Kong and Guangdong are provinces/regions that typically exhibit these two groups of overlying cause.

There was a notable sex disparity in the point prevalence in the younger age group in 1990, while this was delayed to the 45-to-49-year age group 30 years later, when LBP was the most common cause of physical-labour-related disability in young and middle-aged populations [3,30]. Thus, it seems that in 1990, more women engaged in manual labour after high school, or they even left formal education at that time. However, currently, younger people tend to choose their vocations after bachelors and graduate degrees, obtaining higher education levels and undertaking more mental rather than physical labour [24]. However, in the 55-to-59-year age group, the larger sex disparity in LBP remained stable, which may be due to the menopause [33]. This phenomenon is also reflected by the prevalence in the 15-to-49-year (pre-menopausal) age group being 6.8% in 1990 and 5.1% in 2019, while it was 18.4% in 1990 and 13.0% in 2019 in the 50-to-69-year (menopausal) age group.

Low back pain-related YLDs mainly depended on the age-standardised prevalence of LBP, the population size, and life expectancy. Over the three decades considered, the age-standardised prevalence of LBP decreased by 29.1%. However, China has the second-largest population in the world (second only to India), and life expectancy has also increased (from 70.5 for women and 66.8 for men in 1990 to 79.4 for women and 73.6 for men in 2019; https://data.stats.gov.cn/). Consequently, due to the greater proportional population increase, the number of YLDs increased by 2.0 million even though the age-standardised prevalence decreased by as much as 29.0%. The peak age category for the number of YLDs was delayed by 10–15 years in 2019 when compared to 1990. This can probably be attributed to the extended life expectancy in 2019 (a mean increase of 7.7 years compared to 1990) and the baby-boom period in the 1970s [9,34].

Consistent with the rest of the world, in China, LBP has consistently been the leading cause of YLDs since 1990 [9,15,30]. It took the leading rank in all provinces/regions in 1990, but it dropped in some provinces (such as Zhejiang, Shanghai, Yunnan, Shandong, and Hubei) in 2019 and was replaced by age-related hearing loss and headache disorders. We presented the data in Figure S1 in the **Online Supplementary Document**. In total, the decreased prevalence of LBP in these provinces probably correlated with the development of medical care, self-protection of the spine, adequate physical exercise, and national health strategies [35,36].

Over the 30-year period, 15 of the 33 provinces/regions had increased LBP prevalence in the 5-to-14 age group, and all provinces/regions had decreased prevalence in other age groups. It has been noted that although 21 trials with 30 850 adults have considered LBP prevention [37], leading to moderate-quality evidence for exercise and education, the evidence relating to children – particularly concerning primary prevention – is much more limited [38]. A 2014 systematic review of 11 trials involving 2700 children showed moderate-quality evidence for the role of effective education and very-low-quality evidence for the use of ergonomically designed furniture, which is unpromising and should be paid more attention [39,40].

Socio-demographic index can be used to better reflect the burden of disease, the degree of healthy development, and the level of economic ability [4,41]. The relationship in this study can be summarised as the YLDs being negatively correlated with SDI in the lower SDI interval, being relatively stable within middle values of SDI, and being slightly positively correlated with SDI in the higher-value interval, in which the number of YLDs was still lower than that in the low-SDI interval. As a health-discrepancy index, the YLD rate reflects the loss of healthy life caused by disease. The more developed the socioeconomic culture, the greater the life expectancy, and thus the longer the time spent living with disease [42,43]. This is the reason that the YLD rate decreases in low-SDI countries as SDI increases but also increases in high-SDI countries with longer life expectancy.

As a non-communicable disease, the burden of LBP is multifactorial, although it has particular common features [3,22]. More specifically, primary and secondary industries mainly dominate in regions with lower SDI values and urbanisation rates, where manual labour forms the majority of employment [44]. There is then a relative balance between manual labour and mental work as the SDI increases. In higher-SDI regions, the proportion of mental work, a faster-paced life, and a higher exposure rate to LBP increase in tertiary industries [45,46]. Therefore, three SDI strata, which to a great extent reflect the level of SDI and industrial structure, are defined according to the administrative divisions defined by the National Development and Reform Commission of China. Here, the western and central regions fall into the lower-SDI category, while eastern China has a larger proportion of tertiary industry, and the respective relationship between YLDs and SDI basically corresponds to the previous explanation [47,48]. Interestingly, it was foreseeable that the LBP-related YLD rate would rise with increasing SDI in China during the decades of growth since the turning point in the prevalence of this complaint. Based on this time, studies considering examining the burden of LBP over longer time periods are essential.

As previously noted, over the three decades considered, LBP was always the leading cause of disability [6,41]. The continued increase in disability caused by LBP is a worldwide burden, especially in low- and middle-income countries, where the public health and social systems are poorly equipped to deal with such a burden in addition to that caused by infectious diseases [3]. In China, despite the 30-year decrease in age-standardised prevalence, the number of people living with LBP has still increased due to the increased population, increasing the burden on the government. However, evidence relating to prevention has mainly been collected for adults in high-income countries. Therefore, it is still necessary for policymakers to develop effective solutions for LBP in China, especially at this key time.

Some limitations should be noted. It was reported that the GBD causes of LBP may induce selection bias because they rely on including representative studies from hospitals [9]. The situation in China is serious in comparison with high-income countries because epidemiological surveys of LBP have seldom been performed. In recent years, the CHARLS database has increased the generalisability and reliability of the available data [49]. Furthermore, the GBD data relating to LBP is published only on an annual basis, and this can cause discrepancies from real-world analysis. However, this problem was alleviated by updating and incorporating clinical records from hospitals in more locations. Finally, LBP is a symptom with a wide range of causes, including specific causes such as lumbar disc hernia and nonspecific types, and the treatment methods are distinctive for each [32]. Therefore, a GBD system or epidemiological screening focusing on specific causes would be more meaningful.

## CONCLUSIONS

In conclusion, over the three decades considered, LBP was ranked as the leading cause of YLDs in China. China has the largest number of people with LBP in the world, although the age-standardised prevalence rate and YLDs have decreased since 1990. We found the prevalence and YLD rates to vary by gender, different age groups, and different provinces. We found the prevalence and YLD rates to increase in children and postmenopausal women. There was a trend of a decrease in the YLD rate with increasing SDI, but this appeared to begin to climb again at higher SDI values. LBP is thus still a notable public-health concern, and it is a key time to call for action for prevention and treatment.

## Additional material


Online Supplementary Document
