# Supplementary material for: Evaluation of three decades of the burden of low back pain in China before COVID-19: Estimates from the Global Burden of Disease Database 2019
Source: J Glob Health. 2024 Mar 15;14:04006. doi: 10.7189/jogh.14.04006 (PMC10940963; doi:10.7189/jogh.14.04006)
Supplement: Online Supplementary Document [file jogh-14-04006-s001.pdf]

Table S1 The prevalent number of YLDs caused by LBP by countries/regions all over the world (1990 and 2019)

Countries	men						women						Both					
	1990			2019			1990			2019			1990			2019		
	Number (×1,000)	UUI	LUI	Number (×1,000)	UUI	LUI	Number (×1,000)	UUI	LUI	Number (×1,000)	UUI	LUI	Number (×1,000)	UUI	LUI	Number (×1,000)	UUI	LUI
Afghanistan	295.44	336.29	258.99	908.85	1040.78	785.88	299.41	342.01	259.02	848.78	974.68	730.87	594.85	672.49	520.57	1757.63	2005.76	1520.20
Albania	144.17	165.05	125.33	167.54	190.82	147.73	140.77	159.62	123.42	170.66	194.26	149.84	284.94	323.98	249.66	338.20	384.75	298.17
Algeria	663.29	755.35	576.43	1523.29	1727.26	1325.33	635.15	726.10	551.94	1415.51	1609.90	1232.18	1298.44	1469.54	1131.66	2938.81	3336.91	2560.18
American Samoa	1.37	1.55	1.18	1.95	2.21	1.69	1.71	1.94	1.48	2.67	3.00	2.36	3.08	3.47	2.68	4.62	5.20	4.05
Andorra	2.65	3.09	2.29	4.53	5.25	3.86	3.07	3.51	2.67	5.81	6.70	5.08	5.72	6.58	4.99	10.34	11.87	8.98
Angola	213.68	243.49	184.82	585.67	670.92	510.45	217.95	249.09	189.19	669.02	765.16	583.21	431.63	491.88	374.44	1254.69	1426.19	1096.57
Antigua and Barbuda	1.38	1.58	1.20	2.55	2.92	2.23	2.20	2.48	1.94	3.88	4.43	3.37	3.59	4.04	3.15	6.43	7.29	5.62
Argentina	936.79	1079.59	807.57	1408.17	1624.09	1217.87	1367.65	1563.60	1194.30	2053.99	2348.66	1782.57	2304.45	2650.74	2013.43	3462.16	3975.48	3021.74
Armenia	104.38	119.27	91.50	120.85	138.03	106.74	144.99	165.83	127.96	171.55	196.16	150.76	249.37	282.89	219.88	292.40	333.12	259.18
Australia	872.49	970.24	774.76	1206.66	1389.74	1046.87	958.31	1066.53	858.45	1469.54	1677.16	1274.76	1830.80	2030.34	1639.18	2676.19	3061.07	2339.33
Austria	320.01	367.85	278.40	372.52	435.74	318.34	498.76	564.32	440.86	576.43	654.59	506.73	818.76	926.52	724.41	948.94	1083.90	831.91
Azerbaijan	189.57	214.61	164.68	355.96	411.67	308.84	258.47	292.55	226.59	441.17	504.55	385.48	448.04	507.91	393.62	797.14	909.38	700.87
Bahamas	5.61	6.43	4.89	10.26	11.75	8.89	8.54	9.72	7.44	16.02	18.34	13.96	14.14	16.15	12.32	26.28	29.88	22.90
Bahrain	18.57	21.69	15.65	80.23	93.70	67.71	10.81	12.43	9.28	39.97	46.06	34.25	29.38	33.82	25.04	120.20	139.07	102.20
Bangladesh	2377.64	2696.39	2060.38	4186.89	4759.26	3694.67	2789.45	3164.83	2434.08	5990.05	6828.55	5238.81	5167.09	5847.76	4507.39	10176.93	11585.07	8928.31
Barbados	6.49	7.32	5.68	9.45	10.75	8.29	10.63	12.09	9.35	15.06	17.25	13.16	17.12	19.35	15.04	24.51	28.09	21.52
Belarus	405.56	466.28	352.07	433.36	499.57	380.00	644.22	731.42	567.15	678.02	770.28	600.69	1049.78	1192.09	927.46	1111.38	1272.15	987.59
Belgium	469.74	538.33	406.99	564.86	655.55	492.62	694.51	794.65	609.11	827.76	942.75	728.41	1164.25	1324.62	1020.79	1392.62	1596.08	1222.27
Belize	3.66	4.15	3.15	9.91	11.32	8.62	4.91	5.57	4.29	14.09	16.04	12.20	8.57	9.64	7.46	24.00	27.23	20.83
Benin	96.65	110.10	83.87	268.58	307.72	232.14	101.55	116.44	88.53	272.71	309.03	236.49	198.19	225.52	173.31	541.30	617.02	469.31
Bermuda	1.70	1.93	1.47	2.26	2.60	1.97	2.62	2.96	2.30	3.58	4.08	3.17	4.31	4.88	3.79	5.84	6.68	5.16
Bhutan	11.86	13.54	10.31	20.42	23.39	17.85	15.64	17.87	13.64	27.06	30.84	23.62	27.50	31.17	23.93	47.48	53.93	41.65

Bolivia (Plurinational State of)	128.94	146.84	111.94	294.95	336.54	255.56	166.10	190.03	144.50	369.51	418.71	321.88	295.04	334.05	257.81	664.46	752.26	582.24
Bosnia and Herzegovina	200.45	228.88	175.93	193.57	224.43	169.50	207.83	236.51	182.18	206.10	235.61	181.59	408.28	464.93	361.37	399.67	457.71	353.07
Botswana	23.99	27.41	20.87	57.55	66.20	49.93	25.19	28.49	22.01	56.09	63.84	48.77	49.18	55.74	43.07	113.64	129.36	99.06
Brazil	4278.48	4850.42	3720.50	7939.89	9018.94	6937.02	5232.83	5944.34	4534.43	10253.88	11624.59	9010.05	9511.31	10770.34	8253.99	18193.77	20574.64	15921.83
Brunei Darussalam	7.36	8.59	6.22	16.05	18.71	13.60	9.94	11.50	8.55	22.14	25.60	19.00	17.30	19.99	14.77	38.19	44.05	32.81
Bulgaria	528.13	605.11	464.88	475.92	543.60	420.69	523.73	598.18	458.83	488.82	561.04	430.67	1051.86	1196.66	928.72	964.74	1100.69	852.43
Burkina Faso	188.51	214.46	164.13	461.00	530.62	401.34	203.16	229.88	178.17	508.14	578.66	440.06	391.68	442.21	344.60	969.15	1108.07	848.66
Burundi	113.62	128.37	98.53	275.24	316.58	240.17	99.29	113.43	86.95	199.92	227.45	174.10	212.92	240.37	186.07	475.17	542.03	414.38
Cabo Verde	7.50	8.53	6.54	16.61	18.94	14.46	8.96	10.19	7.90	16.64	18.91	14.64	16.45	18.64	14.51	33.25	37.69	29.25
Cambodia	238.12	270.45	207.38	507.75	573.64	441.71	362.82	412.61	317.71	715.09	813.82	630.24	600.94	680.65	525.49	1222.85	1387.74	1073.24
Cameroon	238.75	273.48	207.90	706.65	811.20	611.77	237.08	271.22	206.48	683.81	784.39	590.56	475.83	541.13	416.09	1390.45	1590.85	1212.04
Canada	1125.40	1165.47	1090.14	1798.20	2069.61	1560.80	1431.72	1478.73	1392.34	2452.59	2828.10	2138.01	2557.12	2638.19	2485.61	4250.79	4870.94	3709.62
Central African Republic	55.74	63.46	48.37	113.40	130.16	98.59	63.80	73.04	55.70	124.63	141.91	108.02	119.54	136.04	104.36	238.03	271.98	208.47
Chad	136.69	154.27	120.12	352.14	402.32	308.35	139.36	157.80	122.69	330.64	379.64	286.55	276.05	311.49	243.78	682.78	779.58	594.57
Chile	345.56	400.29	298.16	611.55	714.34	532.03	501.78	577.89	436.52	872.42	1003.27	757.53	847.33	972.68	734.60	1483.97	1709.83	1294.14
China	32373.59	36679.94	28241.88	37567.53	42858.89	33023.98	42924.83	48442.15	37602.28	53771.90	61618.27	47456.18	75298.42	85123.50	65995.27	91339.43	104119.89	80527.99
Colombia	706.51	810.20	608.27	1407.11	1585.27	1232.18	1333.20	1531.74	1158.52	2610.82	2941.76	2297.00	2039.71	2337.34	1772.95	4017.93	4498.77	3561.63
Comoros	10.89	12.23	9.64	21.88	24.78	19.14	8.52	9.55	7.49	18.01	20.43	15.67	19.41	21.73	17.18	39.90	45.28	34.91
Congo	48.76	55.09	42.70	127.48	146.03	110.39	54.88	62.44	48.07	132.68	151.34	115.48	103.64	117.16	90.92	260.16	295.46	226.50
Cook Islands	0.62	0.70	0.55	0.77	0.87	0.68	0.75	0.85	0.66	1.09	1.24	0.97	1.37	1.54	1.21	1.86	2.11	1.64
Costa Rica	60.48	69.33	52.51	121.68	139.26	106.82	111.60	127.56	97.42	224.07	254.24	195.77	172.08	195.39	150.36	345.75	390.42	302.58
Croatia	265.29	296.68	238.87	263.73	301.56	230.94	337.61	370.89	307.18	321.70	370.07	283.45	602.91	667.39	546.96	585.42	670.87	516.69
Cuba	296.04	336.90	260.00	362.50	404.22	323.80	469.80	534.41	412.78	633.22	696.08	575.60	765.84	866.82	672.94	995.72	1093.78	905.76
Cyprus	34.48	39.67	29.91	68.57	78.33	59.57	44.73	51.20	38.81	90.53	103.56	78.88	79.21	90.56	69.00	159.10	181.93	139.20

Czechia	570.38	643.13	504.06	700.63	802.63	615.69	645.14	731.46	572.20	740.67	842.59	651.49	1215.51	1371.82	1081.44	1441.30	1640.99	1270.39
Côte d'Ivoire	264.97	301.89	231.81	649.36	744.61	562.84	218.74	250.45	190.80	560.08	645.89	486.29	483.71	548.99	424.44	1209.44	1385.85	1055.30
Democratic People's Republic of Korea	567.16	645.24	492.61	915.90	1045.71	799.19	899.43	1016.92	787.02	1346.07	1533.65	1188.08	1466.59	1662.22	1289.58	2261.97	2568.29	2004.60
Democratic Republic of the Congo	753.07	862.83	653.82	1865.69	2136.25	1621.33	863.03	979.58	756.96	1965.02	2236.60	1698.52	1616.10	1836.52	1413.53	3830.71	4378.88	3320.43
Denmark	312.51	339.78	290.56	371.51	430.68	320.36	416.76	447.83	388.21	475.81	547.77	413.95	729.27	785.26	680.26	847.32	972.93	737.26
Djibouti	10.19	11.70	8.79	33.53	38.32	29.12	6.65	7.64	5.74	22.43	25.72	19.26	16.84	19.37	14.56	55.96	64.09	48.63
Dominica	1.74	1.97	1.52	2.14	2.44	1.87	2.71	3.06	2.37	2.92	3.31	2.59	4.45	5.01	3.92	5.06	5.71	4.48
Dominican Republic	148.47	168.40	130.23	289.07	326.66	251.51	181.82	206.92	158.03	359.93	407.63	314.44	330.29	372.95	288.57	649.00	735.35	568.71
Ecuador	208.37	234.06	180.35	425.66	475.87	382.44	243.22	275.41	212.56	519.99	574.52	473.65	451.59	507.75	393.93	945.65	1045.71	859.68
Egypt	1567.81	1777.93	1354.97	3411.80	3869.75	2963.73	1456.29	1656.40	1264.07	2952.34	3369.10	2572.83	3024.11	3419.90	2632.48	6364.14	7233.28	5533.17
El Salvador	92.90	105.58	80.46	139.35	158.18	120.83	184.70	210.50	161.09	289.82	329.81	251.93	277.61	313.98	242.18	429.17	486.28	376.14
Equatorial Guinea	8.07	9.22	7.00	29.90	34.59	25.59	10.32	11.73	9.00	29.53	33.55	25.84	18.39	20.82	16.08	59.43	67.72	51.47
Eritrea	50.69	57.87	44.06	141.53	163.35	121.73	42.86	49.41	37.35	112.98	128.33	98.32	93.55	107.29	81.68	254.51	291.05	221.33
Estonia	62.85	70.41	55.68	63.37	72.44	55.55	89.64	101.52	79.65	90.86	103.51	80.51	152.48	171.93	135.91	154.23	174.54	136.84
Eswatini	13.40	15.21	11.71	23.46	26.97	20.49	13.04	14.76	11.43	23.14	26.15	20.16	26.44	29.80	23.23	46.59	52.98	40.66
Ethiopia	981.72	1112.86	855.92	2221.50	2528.06	1927.53	918.80	1042.13	805.19	1930.62	2192.26	1678.54	1900.52	2156.92	1664.44	4152.13	4723.80	3616.66
Fiji	22.03	25.05	19.22	32.04	36.62	27.98	28.60	32.43	24.99	43.00	48.63	37.78	50.62	57.21	44.33	75.05	85.23	65.63
Finland	215.92	247.28	188.73	261.14	299.10	229.76	307.64	347.41	270.67	363.93	413.15	320.36	523.56	593.76	461.16	625.07	708.58	552.64
France	2426.38	2765.26	2116.98	3050.49	3506.54	2659.06	4043.04	4571.72	3590.80	5243.78	5966.75	4611.07	6469.42	7323.29	5723.37	8294.27	9446.37	7317.84
Gabon	21.51	24.60	18.89	43.80	49.88	38.00	24.36	27.66	21.46	48.96	55.62	42.89	45.87	51.99	40.42	92.77	104.71	81.21
Gambia	20.36	23.53	17.64	49.01	56.37	42.68	18.12	20.64	15.72	48.33	55.04	41.76	38.48	44.14	33.47	97.34	111.04	84.94
Georgia	163.14	185.36	143.46	133.59	153.46	116.71	233.70	266.39	206.38	189.02	216.51	167.61	396.84	453.16	350.37	322.61	368.31	285.26
Germany	4238.53	4856.44	3698.92	5002.61	5727.58	4381.98	6443.91	7280.58	5691.24	7171.38	8155.71	6320.34	10682.44	12120.81	9438.80	12174.00	13886.21	10743.42
Ghana	278.45	315.75	244.19	630.63	711.27	558.54	269.50	306.92	235.07	694.82	782.99	613.38	547.95	619.76	481.31	1325.45	1486.60	1172.95
Greece	449.32	513.47	390.88	508.64	586.11	442.16	651.02	742.29	570.14	773.33	891.30	678.78	1100.34	1249.79	960.89	1281.97	1470.29	1123.59

Greenland	2.87	3.35	2.44	3.19	3.70	2.76	2.91	3.36	2.51	3.51	4.05	3.05	5.78	6.69	4.99	6.70	7.71	5.81
Grenada	1.85	2.09	1.62	3.03	3.47	2.66	2.91	3.28	2.56	4.18	4.76	3.64	4.75	5.34	4.20	7.20	8.19	6.32
Guam	4.55	5.21	3.92	6.75	7.63	6.00	5.19	5.86	4.54	8.80	9.88	7.76	9.73	11.04	8.51	15.56	17.42	13.81
Guatemala	158.50	180.23	138.80	400.49	456.50	346.42	238.57	271.52	208.08	689.39	784.31	599.38	397.07	447.90	348.10	1089.88	1236.84	950.62
Guinea	139.52	159.08	122.23	273.09	309.94	237.51	140.46	159.36	122.23	279.53	318.91	243.20	279.98	317.45	245.05	552.62	628.25	482.31
Guinea-Bissau	20.04	22.89	17.31	39.72	45.69	34.56	20.76	23.81	18.08	41.54	47.47	35.95	40.80	46.59	35.54	81.26	93.51	70.72
Guyana	15.77	18.08	13.58	19.16	21.88	16.74	22.63	25.81	19.56	28.38	32.28	24.75	38.40	43.78	33.17	47.53	53.98	41.76
Haiti	121.50	138.24	105.87	260.37	298.32	227.31	180.56	205.53	156.80	404.66	464.11	351.61	302.06	340.98	262.65	665.03	759.52	581.28
Honduras	78.00	88.71	67.38	204.98	232.86	177.89	146.61	167.96	127.24	383.37	437.26	332.07	224.61	255.20	195.01	588.35	667.45	510.58
Hungary	586.47	661.45	517.01	665.08	760.87	586.91	614.88	696.15	545.27	677.69	774.17	600.02	1201.35	1356.16	1065.22	1342.77	1524.87	1197.84
Iceland	11.92	13.74	10.38	16.91	19.47	14.73	15.95	18.17	13.96	23.06	26.35	20.28	27.88	31.60	24.47	39.96	45.64	35.30
India	16485.89	18706.66	14373.60	22802.57	25941.72	19959.88	29113.82	33024.93	25436.12	45134.97	51300.44	39601.80	45599.71	51579.04	39871.07	67937.53	77131.45	59709.07
Indonesia	4987.42	5648.67	4370.53	8638.70	9827.71	7573.43	7587.72	8551.53	6660.89	12862.13	14623.80	11297.02	12575.13	14169.56	11055.73	21500.83	24357.42	18906.51
Iran (Islamic Republic of)	1952.05	2207.24	1711.47	3761.77	4259.10	3310.14	1749.11	1984.43	1530.13	3653.27	4130.29	3219.19	3701.16	4181.66	3239.98	7415.04	8393.69	6524.55
Iraq	438.46	498.58	379.10	1289.64	1468.86	1119.00	411.47	466.52	358.55	1186.73	1344.87	1028.60	849.93	963.88	738.67	2476.37	2802.29	2157.32
Ireland	167.22	190.83	146.03	259.25	298.20	226.12	218.10	248.97	191.45	343.02	386.95	302.37	385.33	439.19	338.30	602.27	684.57	531.44
Israel	192.61	221.03	168.48	383.13	439.36	332.88	271.27	306.21	239.93	543.36	616.53	475.13	463.88	523.42	409.05	926.49	1053.06	811.35
Italy	2719.65	3130.34	2370.73	3338.34	3845.01	2904.12	4366.52	4962.93	3830.33	4869.21	5555.66	4284.15	7086.17	8087.35	6208.10	8207.56	9358.93	7183.34
Jamaica	52.98	59.86	46.13	80.56	91.54	70.31	78.89	89.13	69.44	117.42	133.00	103.41	131.87	148.57	115.83	197.98	224.54	174.30
Japan	6101.41	7011.02	5289.38	6837.68	7804.98	5956.10	11083.95	12602.86	9761.17	12019.92	13604.67	10623.60	17185.37	19646.75	15056.84	18857.61	21406.52	16597.34
Jordan	93.97	108.21	81.04	396.81	456.58	345.36	81.60	93.45	70.74	320.33	363.66	278.28	175.57	199.97	151.93	717.15	816.40	622.95
Kazakhstan	493.64	557.60	433.71	616.31	707.86	538.86	634.93	715.44	558.26	796.07	910.06	698.21	1128.57	1273.61	991.92	1412.37	1610.29	1242.29
Kenya	471.69	535.24	411.92	1290.76	1468.50	1126.07	383.33	433.66	336.11	966.96	1092.87	843.30	855.02	969.41	747.13	2257.72	2555.49	1969.00
Kiribati	1.92	2.18	1.67	3.50	4.01	3.04	2.76	3.12	2.41	5.22	5.93	4.57	4.68	5.28	4.10	8.72	9.85	7.65
Kuwait	62.64	73.49	53.48	188.18	217.93	161.00	40.67	46.79	34.45	158.18	183.62	135.29	103.31	118.76	87.98	346.36	398.41	297.42
Kyrgyzstan	111.33	126.66	97.41	181.80	206.66	158.09	153.86	173.51	135.35	233.33	266.69	203.28	265.18	298.81	233.22	415.13	471.18	360.86

Lao People's Democratic Republic	99.11	111.62	87.51	209.98	238.22	183.93	130.43	147.02	115.21	267.33	303.74	234.66	229.54	258.65	203.52	477.30	538.39	418.56
Latvia	102.07	115.76	89.51	89.64	103.28	78.99	172.19	195.24	152.28	150.41	172.18	132.94	274.26	309.44	242.43	240.05	273.83	213.21
Lebanon	87.09	99.66	75.71	166.24	188.43	146.52	86.40	98.34	75.37	180.23	204.56	158.66	173.49	197.17	151.47	346.48	392.39	304.63
Lesotho	36.60	41.51	32.08	48.06	54.90	41.91	37.72	42.94	33.12	48.01	54.61	41.83	74.32	84.06	65.30	96.06	108.76	83.85
Liberia	47.15	53.62	41.15	115.87	132.55	100.67	43.70	49.82	38.22	105.63	121.73	90.36	90.84	102.75	79.44	221.50	252.41	192.15
Libya	113.94	130.71	98.57	260.10	298.40	223.44	91.49	104.20	79.82	230.11	263.14	200.06	205.43	234.67	179.16	490.21	561.13	423.63
Lithuania	150.19	170.89	131.65	136.52	157.23	119.98	224.86	256.46	197.72	214.03	244.85	188.85	375.05	426.05	330.88	350.55	400.40	311.73
Luxembourg	17.08	19.69	14.70	29.02	33.78	25.01	28.98	32.90	25.53	46.90	53.44	41.31	46.06	52.61	40.34	75.92	87.23	66.55
Madagascar	281.62	319.08	245.93	637.77	728.48	551.49	221.24	252.00	193.62	494.15	567.59	430.25	502.86	567.50	439.38	1131.91	1292.01	985.42
Malawi	206.87	234.36	180.91	393.04	449.87	337.46	169.97	192.85	150.20	333.81	378.87	291.44	376.84	424.66	331.48	726.85	828.35	630.54
Malaysia	441.18	489.43	397.70	1019.40	1162.74	892.26	619.03	686.84	559.42	1349.33	1529.72	1186.99	1060.21	1170.05	960.65	2368.73	2683.05	2081.73
Maldives	4.82	5.47	4.22	18.85	21.57	16.27	5.30	6.07	4.60	14.66	16.64	12.72	10.12	11.49	8.87	33.50	38.10	29.13
Mali	160.69	180.33	141.89	413.37	473.68	361.14	155.11	176.80	136.83	392.23	448.28	339.75	315.81	357.08	278.51	805.60	915.35	702.92
Malta	17.85	20.50	15.42	26.08	30.18	22.81	25.26	28.87	21.99	37.51	42.61	33.24	43.11	49.35	37.51	63.59	72.37	56.28
Marshall Islands	1.07	1.21	0.92	1.80	2.05	1.56	1.34	1.53	1.18	2.27	2.59	1.98	2.41	2.74	2.10	4.08	4.63	3.55
Mauritania	46.92	52.72	41.32	98.75	112.45	86.30	42.32	47.88	37.30	91.82	105.49	80.17	89.25	100.24	78.58	190.58	217.08	167.20
Mauritius	32.73	36.73	28.85	51.69	59.19	45.44	35.52	40.49	31.27	61.38	69.57	53.89	68.25	77.09	60.22	113.07	127.99	99.46
Mexico	1243.19	1425.87	1068.60	2659.83	3023.99	2311.95	2934.60	3337.28	2540.54	5538.66	6287.30	4832.02	4177.79	4751.06	3615.85	8198.48	9313.98	7162.93
Micronesia (Federated States of)	2.67	3.04	2.32	3.36	3.81	2.93	3.38	3.86	2.96	4.47	5.06	3.92	6.05	6.86	5.28	7.83	8.84	6.85
Monaco	1.63	1.88	1.40	2.03	2.35	1.76	2.57	2.94	2.25	3.02	3.46	2.63	4.19	4.80	3.66	5.04	5.80	4.40
Mongolia	50.42	57.63	44.14	98.82	114.35	85.26	62.43	70.65	54.39	125.73	142.63	109.06	112.86	127.90	98.82	224.56	256.55	194.68
Montenegro	30.76	35.12	26.78	37.10	42.96	32.42	33.05	37.58	29.03	39.17	44.72	34.38	63.82	72.47	56.16	76.27	87.69	66.77
Morocco	750.29	849.29	658.75	1411.68	1609.09	1226.46	688.18	782.94	605.05	1358.35	1540.52	1187.80	1438.47	1616.58	1268.96	2770.03	3142.59	2428.12
Mozambique	290.39	331.96	254.57	623.33	711.08	536.29	259.46	296.88	227.12	542.38	617.06	470.80	549.85	628.72	481.81	1165.71	1324.82	1009.84
Myanmar	904.01	1014.94	797.40	1509.50	1695.52	1321.56	1227.67	1396.62	1083.34	2261.25	2550.31	1982.97	2131.68	2392.48	1885.45	3770.74	4239.68	3317.59

Namibia	29.58	33.33	25.93	56.64	64.50	49.05	28.57	32.36	25.12	58.16	66.05	50.74	58.15	65.50	51.29	114.80	130.13	99.95
Nauru	0.27	0.31	0.23	0.29	0.33	0.25	0.33	0.38	0.28	0.39	0.45	0.34	0.60	0.68	0.52	0.68	0.78	0.59
Nepal	463.27	521.20	402.62	801.52	919.29	696.82	580.77	657.00	508.13	1259.13	1439.01	1096.05	1044.03	1171.88	915.83	2060.65	2346.50	1802.84
Netherlands	624.18	715.26	543.85	789.24	914.26	679.30	874.15	992.80	772.66	1131.65	1291.81	990.86	1498.33	1696.59	1319.88	1920.89	2196.40	1674.17
New Zealand	150.86	174.80	129.98	209.78	241.26	182.61	197.85	226.51	171.40	285.19	325.87	249.21	348.71	398.75	302.94	494.97	565.18	432.79
Nicaragua	62.90	72.32	53.89	144.45	165.79	125.40	123.07	141.50	106.93	263.96	301.74	230.02	185.96	212.81	161.46	408.41	464.10	356.85
Niger	159.22	182.05	138.06	437.72	504.16	379.80	147.84	169.38	127.76	433.23	498.18	378.20	307.06	349.99	265.70	870.95	1002.13	760.15
Nigeria	2460.13	2781.24	2159.41	5166.75	5864.28	4519.92	2368.22	2673.93	2080.93	6134.46	6972.69	5369.74	4828.35	5453.12	4241.09	11301.20	12839.10	9885.44
Niue	0.08	0.09	0.07	0.07	0.08	0.06	0.11	0.13	0.10	0.10	0.11	0.09	0.19	0.21	0.17	0.17	0.19	0.15
North Macedonia	88.99	101.41	77.82	121.22	139.37	105.34	95.12	108.80	82.69	122.43	140.40	106.98	184.10	209.54	160.42	243.65	279.51	213.95
Northern Mariana Islands	1.58	1.82	1.35	1.93	2.23	1.68	1.63	1.87	1.39	2.46	2.84	2.13	3.20	3.69	2.76	4.39	5.04	3.82
Norway	190.00	216.84	165.44	249.48	285.26	218.11	265.54	301.71	234.07	328.77	373.64	289.74	455.54	518.72	399.62	578.26	660.37	507.92
Oman	62.07	71.43	52.71	213.96	250.83	178.82	34.55	39.33	30.07	93.62	107.75	80.58	96.62	110.46	83.21	307.58	357.61	259.82
Pakistan	1794.56	2069.20	1528.62	3927.80	4526.20	3320.98	2425.40	2810.68	2059.50	5324.28	6162.34	4543.89	4219.96	4884.87	3602.95	9252.07	10707.60	7943.24
Palau	0.51	0.58	0.44	0.87	1.00	0.75	0.64	0.73	0.56	1.01	1.15	0.87	1.15	1.31	1.00	1.87	2.14	1.63
Palestine	46.05	52.85	40.07	136.84	155.57	119.55	47.64	54.76	41.39	128.85	146.60	112.17	93.69	107.05	81.68	265.70	301.14	232.24
Panama	48.94	55.97	42.62	105.82	121.26	92.45	85.88	97.94	74.69	176.91	200.04	154.93	134.82	153.10	117.43	282.73	320.33	248.08
Papua New Guinea	112.58	128.27	97.89	293.30	334.05	255.78	137.60	155.59	119.43	359.96	411.99	311.91	250.17	282.32	218.16	653.26	743.66	566.90
Paraguay	103.41	117.01	91.18	220.69	250.62	193.68	114.96	129.89	100.93	259.52	294.88	226.55	218.37	245.68	191.92	480.21	542.47	420.81
Peru	440.19	500.22	384.69	907.62	1029.03	790.91	548.97	623.77	478.56	1150.81	1301.02	1013.49	989.15	1123.49	864.83	2058.43	2321.82	1807.89
Philippines	1795.44	2025.82	1569.31	3490.58	3958.42	3048.87	2508.09	2839.01	2198.86	5195.69	5881.32	4575.82	4303.53	4863.55	3769.76	8686.27	9801.42	7646.92
Poland	1838.00	2077.53	1617.82	2287.00	2600.59	2028.35	2462.35	2786.01	2176.52	2829.54	3208.71	2516.57	4300.35	4866.13	3799.46	5116.53	5828.56	4547.47
Portugal	464.16	531.42	406.13	560.71	646.89	494.90	768.66	874.77	674.52	1007.50	1135.13	898.18	1232.82	1403.63	1089.56	1568.21	1774.40	1398.57
Puerto Rico	90.26	102.72	79.08	113.40	128.87	100.00	140.41	158.98	122.94	187.25	212.59	165.99	230.67	261.10	202.07	300.66	340.73	267.03
Qatar	20.98	24.81	17.36	176.65	207.65	149.04	7.20	8.38	6.12	46.10	53.88	39.05	28.18	33.03	23.48	222.75	258.44	188.14
Republic of Korea	1470.15	1701.05	1251.48	2506.29	2891.81	2168.63	2416.95	2778.76	2104.72	3784.99	4361.62	3282.17	3887.10	4465.94	3367.77	6291.27	7244.07	5470.61

Republic of Moldova	159.81	182.14	140.01	168.24	192.54	147.74	235.33	266.01	206.97	242.19	275.61	212.45	395.14	446.53	348.85	410.43	468.24	362.10
Romania	1300.63	1479.65	1136.26	1219.48	1399.78	1064.28	1503.34	1707.99	1322.66	1426.07	1617.38	1256.60	2803.97	3176.10	2460.40	2645.55	3016.05	2344.39
Russian Federation	5311.70	6047.10	4652.85	5789.95	6579.49	5105.10	10473.68	11806.55	9295.41	11765.01	13269.54	10486.42	15785.38	17862.09	13985.65	17554.96	19819.46	15670.16
Rwanda	158.67	179.00	137.77	320.53	365.37	279.31	138.75	159.17	122.16	281.45	321.35	247.26	297.42	336.50	260.63	601.98	684.38	527.91
Saint Kitts and Nevis	0.94	1.07	0.83	1.80	2.07	1.57	1.44	1.63	1.27	2.53	2.89	2.21	2.39	2.68	2.10	4.33	4.96	3.79
Saint Lucia	2.89	3.31	2.50	5.44	6.22	4.74	4.46	5.06	3.91	7.96	9.09	6.96	7.35	8.32	6.42	13.40	15.28	11.72
Saint Vincent and the Grenadines	2.27	2.59	1.97	3.43	3.92	3.00	3.34	3.78	2.94	4.60	5.23	4.03	5.61	6.33	4.91	8.03	9.13	7.06
Samoa	5.10	5.83	4.46	7.21	8.16	6.32	6.35	7.18	5.60	9.18	10.42	8.06	11.44	12.94	10.07	16.40	18.55	14.42
San Marino	1.11	1.28	0.97	1.61	1.85	1.39	1.54	1.78	1.35	2.41	2.75	2.11	2.65	3.04	2.32	4.02	4.58	3.52
Sao Tome and Principe	2.37	2.69	2.07	4.77	5.46	4.15	2.38	2.71	2.09	4.52	5.14	3.93	4.75	5.39	4.17	9.29	10.58	8.10
Saudi Arabia	477.52	549.34	411.64	1581.74	1836.23	1341.12	305.47	347.29	263.28	971.83	1131.08	838.29	782.99	893.40	674.61	2553.57	2947.79	2178.91
Senegal	146.00	164.16	128.60	325.70	371.50	286.05	143.03	162.43	124.87	326.29	371.37	283.48	289.03	325.57	254.56	651.99	739.22	575.13
Serbia	519.41	598.60	455.49	557.15	637.22	488.90	544.71	619.47	477.03	577.62	663.15	503.88	1064.12	1218.28	929.40	1134.78	1300.11	997.67
Seychelles	2.06	2.33	1.82	3.85	4.37	3.37	2.73	3.06	2.41	4.43	5.04	3.91	4.79	5.37	4.26	8.29	9.37	7.28
Sierra Leone	87.77	100.12	76.44	192.24	218.55	166.71	84.88	96.89	73.97	182.40	209.64	158.08	172.66	196.08	150.68	374.64	427.79	325.35
Singapore	88.45	102.35	74.64	208.49	243.25	178.72	140.14	160.43	120.52	291.14	339.00	249.68	228.59	262.44	196.74	499.62	574.93	429.57
Slovakia	286.28	323.67	253.99	346.54	394.83	302.58	286.71	323.97	251.86	352.43	400.51	310.00	572.99	646.08	510.02	698.96	795.29	615.85
Slovenia	107.43	122.14	95.54	137.31	157.37	120.92	111.85	125.69	99.00	134.80	152.98	118.22	219.28	248.39	195.26	272.11	308.71	239.37
Solomon Islands	8.45	9.59	7.31	18.26	20.94	15.80	10.06	11.45	8.81	23.52	26.78	20.40	18.51	20.98	16.19	41.78	47.45	36.30
Somalia	150.82	174.36	130.89	430.85	493.39	371.01	118.49	135.74	103.05	334.36	382.22	289.78	269.31	308.34	235.44	765.21	871.13	663.42
South Africa	756.64	854.18	660.14	1335.28	1514.57	1169.21	892.24	1006.88	784.93	1579.20	1790.04	1391.07	1648.89	1860.42	1448.98	2914.49	3313.53	2555.99
South Sudan	141.09	159.80	122.59	218.57	249.87	189.85	91.25	104.07	79.63	167.98	193.57	145.77	232.34	262.56	203.14	386.56	443.34	335.44
Spain	1773.49	1869.30	1684.86	1711.41	1991.86	1476.47	2262.53	2430.41	2097.15	2910.43	3313.15	2583.59	4036.02	4292.85	3799.32	4621.84	5303.11	4082.36
Sri Lanka	475.48	533.59	418.70	756.02	856.50	661.20	521.84	592.26	454.22	977.46	1105.83	856.01	997.32	1122.98	876.77	1733.48	1949.09	1517.78
Sudan	488.71	554.01	425.84	1080.81	1231.59	937.77	466.27	533.40	402.09	1012.85	1154.28	875.71	954.98	1084.95	830.09	2093.65	2382.92	1817.65
Suriname	8.74	10.00	7.56	15.71	17.95	13.73	12.24	13.86	10.67	23.63	26.89	20.76	20.99	23.83	18.35	39.34	44.74	34.60

Sweden	388.29	433.76	347.25	486.19	556.49	424.62	568.62	626.70	515.86	670.11	758.41	593.43	956.91	1061.17	865.47	1156.30	1314.63	1021.34
Switzerland	484.25	506.90	462.86	517.30	597.18	450.30	685.78	721.37	653.01	726.83	828.79	638.55	1170.03	1225.33	1119.32	1244.13	1427.12	1090.62
Syrian Arab Republic	315.73	358.21	274.58	498.90	570.39	431.66	289.58	329.11	251.52	508.13	578.37	441.50	605.32	686.62	528.06	1007.02	1147.94	871.60
Taiwan (Province of China)	861.42	914.89	810.76	1084.68	1237.41	949.98	1111.22	1183.40	1004.44	1522.15	1725.19	1342.86	1972.65	2094.74	1830.64	2606.83	2969.81	2305.90
Tajikistan	119.86	136.47	104.83	244.62	280.11	212.09	152.48	173.43	132.97	288.00	330.23	249.83	272.35	309.38	238.58	532.62	608.56	463.35
Thailand	1421.26	1632.37	1236.49	2690.68	3066.82	2369.53	1836.37	2105.91	1600.20	3668.62	4167.06	3214.04	3257.63	3724.10	2859.61	6359.30	7211.89	5580.52
Timor-Leste	18.47	21.05	16.12	33.81	38.33	29.58	21.65	24.91	18.73	41.37	46.84	36.24	40.12	45.66	34.99	75.18	84.88	66.27
Togo	68.93	78.98	59.67	179.37	204.54	154.67	71.43	82.36	62.13	191.61	218.66	166.99	140.36	161.20	122.27	370.98	420.83	321.83
Tokelau	0.05	0.06	0.04	0.05	0.06	0.04	0.07	0.08	0.06	0.07	0.07	0.06	0.12	0.14	0.11	0.12	0.13	0.10
Tonga	2.74	3.09	2.40	3.29	3.72	2.90	3.79	4.30	3.33	4.71	5.32	4.12	6.53	7.36	5.74	8.00	9.00	7.06
Trinidad and Tobago	28.03	31.92	24.47	43.51	49.76	37.81	39.82	45.23	34.58	62.28	70.44	54.49	67.84	76.89	59.09	105.79	119.76	92.66
Tunisia	242.96	275.40	213.38	452.90	519.15	395.35	213.07	241.04	186.47	445.04	507.88	387.51	456.04	514.65	401.45	897.95	1024.03	783.94
Turkey	2657.56	2973.70	2288.94	3976.93	4527.23	3463.38	2083.72	2366.02	1825.10	3753.90	4239.91	3310.67	4741.28	5294.48	4167.29	7730.84	8691.68	6788.73
Turkmenistan	84.94	96.53	74.20	155.59	178.44	135.46	112.30	127.04	98.87	185.85	210.37	162.97	197.24	222.45	173.53	341.43	386.75	299.11
Tuvalu	0.27	0.31	0.24	0.43	0.48	0.38	0.44	0.50	0.39	0.56	0.63	0.49	0.72	0.81	0.63	0.99	1.11	0.87
Uganda	347.22	396.02	301.49	835.62	965.52	719.71	282.12	320.92	243.94	703.24	795.84	612.38	629.34	712.30	545.61	1538.86	1758.69	1338.67
Ukraine	2265.84	2582.65	1998.10	2123.87	2439.83	1882.50	3833.36	4350.58	3398.36	3650.96	4116.77	3232.01	6099.20	6925.65	5420.50	5774.83	6521.74	5125.37
United Arab Emirates	79.17	92.78	66.79	603.80	725.60	500.09	26.74	31.16	22.85	166.53	198.17	139.34	105.91	123.41	89.48	770.33	919.31	641.48
United Kingdom	3050.61	3474.78	2671.09	3502.99	4000.96	3060.62	4294.85	4839.36	3803.98	5150.20	5839.24	4541.97	7345.47	8302.73	6485.66	8653.19	9841.00	7616.30
United Republic of Tanzania	540.09	612.46	471.67	1257.02	1443.28	1090.56	455.65	517.96	397.27	1037.23	1181.75	903.00	995.73	1129.80	874.03	2294.24	2604.46	2004.45
United States of America	17542.90	19890.45	15560.38	22878.61	24893.80	21138.53	23093.51	25985.38	20497.08	29226.82	31650.29	27041.08	40636.41	45823.74	36177.56	52105.43	56404.01	48196.03
United States Virgin Islands	2.54	2.90	2.20	3.24	3.69	2.84	3.93	4.45	3.41	5.36	6.16	4.68	6.47	7.34	5.62	8.60	9.83	7.55
Uruguay	94.55	108.36	82.31	116.78	132.96	101.69	134.11	154.59	116.16	178.08	203.72	155.52	228.66	262.02	198.91	294.86	336.58	257.62

Uzbekistan	468.16	531.47	404.91	938.24	1067.71	816.91	611.19	693.84	536.33	1166.32	1335.59	1017.21	1079.35	1214.32	940.54	2104.56	2387.38	1834.61
Vanuatu	4.39	4.99	3.82	9.99	11.36	8.74	5.29	6.02	4.64	12.65	14.33	11.08	9.68	11.00	8.48	22.64	25.62	19.93
Venezuela (Bolivarian Republic of)	335.30	382.89	289.17	668.87	755.78	582.62	697.35	793.91	602.94	1292.42	1469.19	1136.32	1032.64	1170.53	893.77	1961.29	2218.14	1724.37
Viet Nam	1577.52	1772.65	1389.08	3444.98	3914.04	3013.21	2223.76	2512.94	1965.35	4676.53	5313.79	4089.44	3801.28	4271.08	3357.62	8121.52	9185.15	7138.26
Yemen	306.43	348.88	265.01	817.81	941.80	705.60	298.84	340.23	259.42	785.49	896.15	680.95	605.27	688.30	525.09	1603.30	1831.45	1388.52
Zambia	141.26	159.29	123.98	397.17	458.84	341.35	106.40	120.00	93.48	300.44	342.63	258.56	247.66	278.65	217.47	697.61	797.53	602.13
Zimbabwe	198.50	223.21	174.44	335.70	380.91	289.82	184.80	209.85	162.78	353.38	402.63	310.40	383.30	432.02	338.33	689.08	779.09	603.77

Footnote: LBP: low back pain; UUI: upper uncertainty interval; LUI: lower uncertainty interval

Table S2 The prevalent number of YLDs caused by LBP by countries/regions all over the world (1990 and 2019)

Countries	men						women						Both					
	1990			2019			1990			2019			1990			2019		
	Number (×1,000)	UUI	LUI	Number (×1,000)	UUI	LUI	Number (×1,000)	UUI	LUI	Number (×1,000)	UUI	LUI	Number (×1,000)	UUI	LUI	Number (×1,000)	UUI	LUI
Afghanistan	32.79	44.00	23.05	102.50	138.60	71.18	32.74	44.03	23.33	93.53	125.60	65.51	65.53	88.11	46.45	196.02	264.25	136.06
Albania	16.44	22.48	11.41	18.99	25.85	13.42	15.93	21.35	11.02	19.24	25.85	13.40	32.37	43.74	22.58	38.23	51.60	26.77
Algeria	76.21	103.90	53.16	174.89	235.77	121.38	71.32	96.51	49.87	158.92	214.78	110.17	147.53	198.76	103.23	333.81	447.25	230.16
American Samoa	0.16	0.21	0.11	0.22	0.29	0.16	0.19	0.26	0.13	0.29	0.39	0.21	0.35	0.47	0.24	0.51	0.69	0.36
Andorra	0.30	0.41	0.21	0.52	0.71	0.36	0.35	0.47	0.24	0.65	0.88	0.45	0.65	0.87	0.45	1.16	1.59	0.81
Angola	24.14	32.64	16.68	66.46	90.38	46.33	24.33	32.72	16.95	75.03	100.86	52.66	48.47	64.74	33.62	141.49	190.55	99.47
Antigua and Barbuda	0.16	0.22	0.11	0.29	0.39	0.20	0.25	0.33	0.17	0.44	0.59	0.31	0.40	0.54	0.28	0.73	0.98	0.51
Argentina	107.10	147.04	73.53	160.39	219.10	112.07	153.33	206.99	107.50	229.78	313.76	159.48	260.43	352.18	180.94	390.17	529.54	272.74
Armenia	11.87	15.92	8.14	13.75	18.73	9.74	16.35	21.96	11.42	19.22	25.85	13.59	28.23	37.83	19.59	32.97	44.38	23.55
Australia	98.78	132.07	68.97	135.59	184.73	94.99	106.81	141.45	75.83	163.03	219.34	114.55	205.59	273.44	145.11	298.62	402.40	209.36
Austria	36.25	49.12	25.36	41.93	57.18	29.90	55.28	74.24	39.65	63.70	86.08	45.29	91.53	123.11	64.73	105.63	142.81	75.64
Azerbaijan	21.77	29.25	15.15	40.95	55.87	28.30	29.14	39.23	20.37	49.91	67.89	34.96	50.90	68.93	35.85	90.86	123.41	63.21
Bahamas	0.64	0.87	0.44	1.17	1.60	0.81	0.96	1.29	0.66	1.80	2.42	1.27	1.61	2.14	1.12	2.97	4.01	2.09
Bahrain	2.15	2.94	1.46	9.26	12.76	6.33	1.21	1.64	0.84	4.48	6.08	3.09	3.36	4.54	2.30	13.74	18.77	9.48
Bangladesh	268.98	366.61	184.57	474.29	636.18	330.36	310.23	418.33	216.73	668.50	902.06	468.31	579.21	784.54	404.59	1142.79	1530.93	805.70
Barbados	0.74	1.00	0.52	1.07	1.44	0.75	1.19	1.59	0.83	1.69	2.26	1.19	1.93	2.59	1.35	2.76	3.71	1.93
Belarus	45.78	60.83	31.67	48.95	65.82	34.43	71.61	96.50	50.40	75.31	100.97	53.92	117.39	157.36	82.07	124.26	166.21	87.96
Belgium	53.36	73.29	37.22	63.61	85.77	44.34	77.10	104.28	54.79	91.12	121.80	64.79	130.46	176.84	91.79	154.72	207.05	109.53
Belize	0.42	0.57	0.29	1.13	1.53	0.80	0.55	0.74	0.39	1.59	2.12	1.11	0.97	1.31	0.68	2.72	3.62	1.91
Benin	10.90	14.78	7.75	30.66	41.44	21.49	11.30	15.26	8.00	30.56	41.07	21.45	22.20	29.94	15.75	61.22	82.28	42.99
Bermuda	0.19	0.26	0.14	0.26	0.35	0.18	0.30	0.40	0.21	0.40	0.54	0.28	0.49	0.66	0.35	0.66	0.89	0.47
Bhutan	1.35	1.84	0.94	2.32	3.13	1.61	1.74	2.35	1.22	3.02	4.08	2.14	3.09	4.17	2.16	5.34	7.16	3.75

Bolivia (Plurinational State of)	14.65	19.75	10.16	33.66	45.29	23.26	18.58	25.02	13.07	41.52	55.53	29.09	33.23	44.69	23.30	75.17	100.38	52.50
Bosnia and Herzegovina	22.83	30.75	15.79	21.70	29.34	15.21	23.46	31.73	16.43	22.98	30.96	16.23	46.29	62.47	32.48	44.68	60.10	31.59
Botswana	2.71	3.63	1.91	6.50	8.72	4.47	2.81	3.75	2.00	6.20	8.33	4.41	5.52	7.36	3.91	12.70	17.09	8.92
Brazil	484.45	652.14	339.20	899.81	1203.19	631.14	582.66	776.44	411.16	1144.29	1518.83	811.20	1067.11	1431.47	750.27	2044.10	2715.67	1441.81
Brunei Darussalam	0.85	1.16	0.57	1.85	2.54	1.27	1.13	1.53	0.78	2.52	3.44	1.73	1.97	2.68	1.36	4.36	5.98	3.00
Bulgaria	59.48	80.84	41.84	53.53	72.58	37.79	58.99	79.31	41.55	54.53	72.83	38.44	118.47	159.51	83.49	108.06	145.84	76.76
Burkina Faso	21.16	28.56	14.93	52.54	70.76	37.05	22.58	30.17	16.00	57.07	76.30	39.90	43.73	58.47	30.81	109.61	148.09	77.03
Burundi	12.85	17.30	8.93	31.03	41.91	21.79	11.07	14.90	7.79	22.39	29.76	15.72	23.91	32.17	16.75	53.42	71.17	37.70
Cabo Verde	0.85	1.15	0.59	1.90	2.56	1.33	1.00	1.35	0.71	1.87	2.48	1.32	1.85	2.50	1.31	3.77	5.01	2.63
Cambodia	26.97	36.31	18.75	57.98	77.65	40.02	40.58	54.80	28.59	80.29	106.95	55.99	67.56	90.55	47.17	138.27	184.51	95.38
Cameroon	26.89	36.21	18.85	80.42	108.74	56.14	26.40	35.25	18.42	76.54	102.79	53.35	53.29	71.16	37.12	156.96	211.27	110.11
Canada	128.33	169.46	91.03	203.35	273.85	143.27	160.29	209.23	115.41	272.82	368.47	193.64	288.62	378.12	206.02	476.17	641.76	335.65
Central African Republic	6.23	8.34	4.36	12.79	17.17	8.95	7.07	9.51	4.95	13.88	18.75	9.68	13.30	17.78	9.33	26.67	35.92	18.70
Chad	15.43	20.65	10.82	39.89	53.58	27.99	15.55	20.74	10.91	36.96	50.43	26.12	30.98	41.36	21.91	76.85	104.46	54.14
Chile	39.48	53.87	27.19	69.58	94.47	48.56	56.22	75.96	39.07	97.33	131.17	67.72	95.70	129.47	66.13	166.91	226.32	115.67
China	3720.13	5011.47	2606.66	4307.31	5857.23	3017.05	4832.72	6431.64	3400.52	6026.80	8179.08	4270.86	8552.86	11419.16	6028.31	10334.10	14004.81	7329.88
Colombia	80.76	108.99	55.75	160.19	216.66	112.72	150.65	202.39	104.05	294.52	394.39	205.34	231.41	312.34	160.42	454.71	609.19	317.35
Comoros	1.23	1.66	0.86	2.50	3.35	1.74	0.95	1.29	0.67	2.02	2.72	1.43	2.18	2.94	1.53	4.52	6.08	3.17
Congo	5.51	7.47	3.87	14.53	19.70	10.09	6.09	8.15	4.29	14.80	20.12	10.25	11.60	15.54	8.17	29.33	39.39	20.29
Cook Islands	0.07	0.10	0.05	0.09	0.12	0.06	0.08	0.11	0.06	0.12	0.16	0.09	0.16	0.21	0.11	0.21	0.27	0.15
Costa Rica	6.92	9.35	4.78	13.84	18.68	9.73	12.59	16.97	8.76	25.23	34.10	17.69	19.51	26.21	13.62	39.07	52.86	27.28
Croatia	29.98	40.58	21.05	29.51	39.94	20.78	37.86	50.49	26.60	35.59	48.13	25.09	67.85	90.98	47.41	65.11	88.04	45.75
Cuba	33.74	45.21	23.69	40.92	54.44	28.73	52.70	70.78	37.22	70.61	94.15	50.61	86.44	115.53	60.96	111.53	149.15	79.72
Cyprus	3.94	5.37	2.71	7.82	10.66	5.42	5.01	6.80	3.47	10.11	13.78	7.09	8.95	12.18	6.20	17.94	24.33	12.60
Czechia	64.22	86.52	45.33	78.14	105.61	55.17	71.97	97.36	50.61	81.99	110.75	57.54	136.19	184.77	96.60	160.13	215.85	113.77
Côte d'Ivoire	29.84	40.27	20.65	73.94	99.38	51.49	24.26	32.47	16.89	62.78	84.38	43.52	54.09	72.46	37.72	136.72	184.07	95.53

Democratic People's Republic of Korea	65.78	89.01	45.64	106.01	142.51	73.90	101.60	136.23	71.66	150.83	200.20	107.05	167.39	224.08	118.37	256.84	341.31	181.23
Democratic Republic of the Congo	84.34	112.79	59.45	211.26	282.86	148.58	95.23	127.45	67.28	218.54	296.30	153.47	179.56	238.14	127.18	429.80	579.27	301.25
Denmark	35.54	46.86	25.32	42.08	57.01	29.39	46.00	60.80	33.16	52.59	70.72	37.34	81.54	107.66	58.04	94.67	127.33	66.89
Djibouti	1.17	1.58	0.82	3.85	5.23	2.67	0.75	1.01	0.52	2.54	3.45	1.77	1.92	2.57	1.33	6.38	8.61	4.42
Dominica	0.20	0.27	0.14	0.24	0.32	0.17	0.30	0.41	0.21	0.33	0.44	0.23	0.50	0.68	0.35	0.57	0.76	0.40
Dominican Republic	16.97	22.72	11.84	33.01	44.31	23.15	20.55	27.68	14.43	40.55	54.53	28.48	37.51	50.39	26.34	73.56	98.97	51.49
Ecuador	23.82	32.04	16.54	48.59	64.91	34.10	27.40	36.67	19.20	58.37	77.72	41.36	51.22	68.65	35.82	106.96	142.07	75.70
Egypt	180.18	242.80	123.69	392.38	530.74	272.85	163.33	221.49	114.61	332.29	444.17	232.09	343.51	460.01	237.30	724.67	974.42	504.74
El Salvador	10.47	13.99	7.38	15.70	20.86	11.06	20.73	27.91	14.37	32.51	43.80	22.87	31.20	41.71	21.84	48.22	64.77	33.80
Equatorial Guinea	0.90	1.21	0.63	3.40	4.57	2.37	1.14	1.52	0.81	3.29	4.41	2.31	2.04	2.72	1.45	6.69	8.99	4.69
Eritrea	5.68	7.60	4.01	16.08	21.85	11.10	4.77	6.40	3.35	12.66	16.82	8.94	10.45	14.02	7.35	28.73	38.65	20.08
Estonia	7.07	9.47	4.98	7.14	9.52	4.99	9.96	13.31	7.02	10.02	13.34	7.12	17.03	22.81	12.02	17.16	22.97	12.19
Eswatini	1.52	2.05	1.07	2.63	3.55	1.83	1.46	1.94	1.02	2.55	3.39	1.82	2.98	3.97	2.07	5.17	6.89	3.64
Ethiopia	110.89	149.16	78.35	252.55	338.77	176.72	102.58	137.97	71.71	217.36	290.71	151.73	213.47	285.44	149.46	469.91	624.49	326.67
Fiji	2.51	3.39	1.73	3.63	4.94	2.57	3.21	4.32	2.25	4.78	6.37	3.39	5.73	7.73	4.01	8.40	11.21	5.99
Finland	24.56	33.31	16.97	29.40	40.00	20.70	34.08	45.54	24.41	40.01	54.48	28.54	58.63	78.66	41.34	69.41	94.46	49.66
France	276.62	374.06	194.69	346.59	468.45	241.40	448.64	602.94	315.73	580.83	778.49	409.14	725.26	975.75	511.23	927.42	1259.72	653.33
Gabon	2.44	3.27	1.71	4.97	6.69	3.44	2.70	3.61	1.92	5.45	7.25	3.81	5.14	6.85	3.62	10.42	13.93	7.26
Gambia	2.32	3.15	1.60	5.59	7.54	3.88	2.02	2.72	1.42	5.38	7.32	3.75	4.34	5.87	3.03	10.97	14.88	7.67
Georgia	18.61	25.13	12.98	15.03	20.18	10.61	26.31	35.87	18.37	20.96	28.36	14.95	44.93	60.88	31.44	35.99	48.36	25.55
Germany	483.53	657.54	337.36	564.06	762.98	392.47	714.64	959.63	505.67	788.35	1049.23	553.98	1198.17	1606.25	844.66	1352.41	1805.63	944.97
Ghana	31.67	42.47	22.49	72.03	96.65	50.92	30.12	40.30	21.38	78.03	105.10	54.67	61.79	82.65	44.14	150.06	201.41	106.45
Greece	51.26	69.54	35.71	57.51	78.34	40.49	72.58	97.06	51.14	85.45	115.48	60.15	123.84	167.05	86.91	142.96	194.71	100.30
Greenland	0.33	0.44	0.22	0.36	0.49	0.25	0.32	0.44	0.23	0.39	0.52	0.28	0.65	0.88	0.45	0.75	1.01	0.53
Grenada	0.21	0.28	0.15	0.34	0.47	0.24	0.32	0.43	0.23	0.47	0.63	0.33	0.53	0.71	0.38	0.81	1.09	0.57

Guam	0.53	0.71	0.36	0.77	1.02	0.54	0.59	0.79	0.41	0.98	1.30	0.70	1.11	1.49	0.77	1.75	2.33	1.24
Guatemala	17.91	24.27	12.51	45.41	61.80	31.63	26.70	36.04	18.81	77.41	103.60	53.83	44.60	60.07	31.38	122.81	165.26	85.28
Guinea	15.80	21.13	11.09	31.04	42.14	21.87	15.66	21.03	11.06	31.29	41.97	21.88	31.46	42.16	22.16	62.33	84.01	43.93
Guinea-Bissau	2.26	3.05	1.61	4.53	6.11	3.14	2.32	3.14	1.65	4.65	6.23	3.22	4.58	6.15	3.25	9.18	12.32	6.35
Guyana	1.78	2.39	1.25	2.16	2.88	1.52	2.52	3.39	1.74	3.16	4.19	2.23	4.30	5.77	2.99	5.31	7.13	3.75
Haiti	13.77	18.64	9.66	29.54	40.05	20.58	20.15	26.87	14.24	45.15	60.78	31.29	33.92	45.39	23.87	74.69	100.00	51.69
Honduras	8.88	12.05	6.20	23.35	31.32	16.22	16.48	22.29	11.60	43.20	58.58	29.84	25.36	34.20	17.81	66.55	89.64	46.40
Hungary	65.88	88.51	46.58	74.73	101.59	52.67	68.52	92.41	48.78	75.30	101.17	53.30	134.40	180.71	95.90	150.03	201.77	106.41
Iceland	1.36	1.86	0.95	1.93	2.60	1.33	1.78	2.41	1.25	2.57	3.47	1.81	3.14	4.25	2.21	4.49	6.07	3.15
India	1851.82	2463.92	1288.71	2549.57	3453.77	1793.64	3202.24	4303.17	2258.30	4954.57	6602.47	3510.54	5054.06	6738.18	3560.55	7504.14	10033.08	5316.00
Indonesia	567.86	752.78	396.88	986.81	1329.23	687.29	851.85	1137.66	598.37	1447.27	1956.75	1018.21	1419.71	1886.54	995.02	2434.09	3256.77	1707.31
Iran (Islamic Republic of)	222.75	298.28	155.68	429.69	579.10	299.27	195.48	262.88	137.74	408.08	547.13	285.31	418.23	558.43	292.45	837.78	1128.83	583.89
Iraq	49.65	67.00	34.49	147.13	200.41	100.29	45.64	61.55	32.27	132.47	177.03	92.85	95.28	127.95	67.11	279.60	377.24	193.90
Ireland	19.05	25.84	13.33	29.44	39.90	20.38	24.28	33.06	17.09	38.15	51.39	26.92	43.33	58.89	30.56	67.59	91.20	47.53
Israel	22.02	29.94	15.41	43.74	58.94	30.54	30.36	40.60	21.37	60.62	81.60	42.74	52.38	70.33	36.57	104.36	140.47	73.22
Italy	307.64	416.39	214.18	375.72	505.89	264.54	482.24	646.36	341.95	536.29	724.20	378.86	789.87	1060.14	553.94	912.01	1232.23	643.68
Jamaica	6.05	8.14	4.24	9.16	12.26	6.40	8.87	11.87	6.27	13.15	17.62	9.26	14.92	20.02	10.56	22.31	29.75	15.74
Japan	700.80	959.17	486.93	774.36	1047.95	543.35	1248.14	1684.69	874.78	1334.48	1796.52	944.76	1948.93	2643.21	1357.36	2108.84	2860.55	1494.75
Jordan	10.83	14.65	7.46	45.78	61.73	31.41	9.14	12.35	6.41	36.00	47.99	25.16	19.96	26.95	13.81	81.78	108.98	56.68
Kazakhstan	56.31	76.00	39.19	70.31	94.89	49.67	70.98	95.89	50.11	89.19	119.84	62.55	127.28	171.42	89.73	159.50	214.30	112.57
Kenya	53.39	71.35	37.48	147.06	196.31	102.43	42.85	56.98	30.17	108.44	144.56	75.89	96.23	128.03	67.70	255.50	339.53	179.02
Kiribati	0.22	0.29	0.15	0.40	0.54	0.28	0.31	0.42	0.22	0.58	0.78	0.41	0.53	0.71	0.37	0.98	1.31	0.69
Kuwait	7.28	9.98	4.92	21.76	29.82	14.76	4.58	6.17	3.16	17.88	24.06	12.15	11.86	16.14	8.09	39.64	53.82	26.98
Kyrgyzstan	12.70	16.92	8.90	20.91	28.43	14.67	17.25	23.22	12.02	26.38	35.42	18.61	29.95	40.24	20.99	47.29	63.27	33.33
Lao People's Democratic Republic	11.26	14.84	7.91	24.04	32.27	16.66	14.63	19.66	10.31	30.18	40.38	20.98	25.89	34.52	18.26	54.22	72.39	37.80
Latvia	11.42	15.30	8.04	10.06	13.61	7.18	19.04	25.68	13.47	16.54	22.16	11.78	30.46	40.86	21.54	26.60	35.72	18.92

Lebanon	9.91	13.40	6.95	18.85	25.42	13.14	9.65	13.08	6.86	20.02	26.62	14.18	19.56	26.49	13.88	38.87	52.12	27.27
Lesotho	4.15	5.56	2.94	5.39	7.22	3.76	4.19	5.64	2.96	5.27	7.02	3.73	8.33	11.22	5.97	10.66	14.22	7.54
Liberia	5.25	7.07	3.71	13.06	17.66	9.15	4.79	6.33	3.39	11.71	15.80	8.23	10.04	13.36	7.10	24.77	33.13	17.34
Libya	13.08	17.78	9.22	29.76	40.50	20.37	10.23	13.83	7.13	25.72	34.94	17.92	23.31	31.46	16.41	55.48	75.27	38.33
Lithuania	16.84	22.68	11.81	15.31	20.60	10.80	24.93	33.44	17.67	23.54	31.32	16.83	41.77	56.46	29.49	38.85	52.06	27.65
Luxembourg	1.95	2.64	1.36	3.30	4.48	2.28	3.22	4.34	2.29	5.19	6.97	3.67	5.17	6.99	3.62	8.49	11.39	6.01
Madagascar	31.79	42.99	22.37	72.83	98.89	50.50	24.70	32.93	17.38	55.58	74.73	38.65	56.49	75.86	39.63	128.42	172.44	89.30
Malawi	23.38	31.27	16.32	44.70	60.98	31.17	18.89	25.12	13.34	37.27	49.32	25.74	42.27	56.10	29.86	81.97	110.20	57.13
Malaysia	50.35	67.34	35.19	116.33	155.48	80.90	69.69	92.49	48.90	151.84	200.93	106.41	120.04	160.12	84.10	268.17	355.21	189.53
Maldives	0.55	0.73	0.39	2.18	2.95	1.50	0.60	0.81	0.41	1.66	2.22	1.15	1.15	1.54	0.80	3.85	5.20	2.64
Mali	18.15	24.38	12.82	47.03	63.97	33.17	17.26	23.32	12.19	43.87	58.95	30.85	35.40	47.83	25.06	90.90	122.77	64.19
Malta	2.04	2.79	1.43	2.96	4.04	2.07	2.82	3.80	2.00	4.15	5.57	2.95	4.87	6.57	3.41	7.11	9.60	5.02
Marshall Islands	0.12	0.16	0.08	0.20	0.27	0.14	0.15	0.20	0.11	0.25	0.34	0.18	0.27	0.36	0.19	0.46	0.61	0.32
Mauritania	5.36	7.25	3.78	11.27	15.39	7.86	4.74	6.35	3.37	10.33	13.98	7.24	10.10	13.56	7.18	21.60	29.25	15.07
Mauritius	3.73	4.98	2.59	5.81	7.91	4.12	3.98	5.32	2.78	6.81	9.19	4.77	7.71	10.35	5.40	12.62	16.98	8.95
Mexico	141.45	190.69	98.75	302.04	402.99	211.64	329.29	442.47	230.49	620.58	826.74	435.47	470.74	633.21	329.75	922.62	1231.35	650.63
Micronesia (Federated States of)	0.30	0.41	0.21	0.38	0.51	0.27	0.38	0.50	0.27	0.50	0.67	0.35	0.68	0.91	0.48	0.88	1.18	0.62
Monaco	0.18	0.25	0.13	0.23	0.31	0.16	0.28	0.38	0.20	0.33	0.45	0.24	0.47	0.63	0.33	0.56	0.76	0.39
Mongolia	5.75	7.78	4.02	11.31	15.31	7.86	7.03	9.47	4.90	14.29	19.09	10.04	12.77	17.20	8.94	25.60	34.63	17.94
Montenegro	3.50	4.76	2.43	4.19	5.65	2.93	3.74	5.07	2.61	4.39	5.93	3.07	7.23	9.77	5.04	8.58	11.59	6.05
Morocco	86.04	115.31	59.76	161.61	217.38	112.05	76.97	103.42	53.73	151.71	204.51	107.33	163.00	217.48	113.69	313.32	423.48	220.86
Mozambique	32.56	43.64	22.72	70.19	94.38	48.80	28.80	38.58	20.19	60.37	81.00	42.83	61.36	82.13	43.05	130.55	175.53	91.42
Myanmar	102.61	137.36	72.85	171.99	232.99	121.09	137.62	184.15	98.16	253.94	339.58	178.84	240.23	320.37	171.45	425.93	571.21	300.38
Namibia	3.33	4.52	2.35	6.39	8.53	4.43	3.17	4.24	2.23	6.45	8.70	4.51	6.51	8.74	4.59	12.85	17.22	8.97
Nauru	0.03	0.04	0.02	0.03	0.04	0.02	0.04	0.05	0.03	0.04	0.06	0.03	0.07	0.09	0.05	0.08	0.10	0.05
Nepal	52.07	69.86	36.57	90.36	122.34	64.00	64.48	86.34	45.50	140.31	187.74	98.58	116.55	156.35	82.06	230.67	310.38	162.58

Netherlands	71.53	95.91	50.09	89.72	122.52	63.21	97.77	130.81	69.06	125.55	168.79	88.25	169.30	228.34	119.21	215.27	292.92	151.46
New Zealand	17.02	23.05	11.91	23.55	32.25	16.52	21.90	29.63	15.16	31.64	42.92	22.29	38.92	52.70	27.08	55.18	74.26	38.72
Nicaragua	7.13	9.76	4.93	16.47	22.39	11.45	13.83	18.74	9.58	29.79	40.17	20.76	20.96	28.28	14.61	46.26	61.99	32.26
Niger	18.12	24.58	12.67	49.90	67.36	34.95	16.52	22.51	11.60	48.56	65.39	34.19	34.65	46.74	24.37	98.46	132.72	69.34
Nigeria	278.61	371.65	196.38	587.37	790.33	411.74	263.45	352.13	186.92	686.15	920.46	485.03	542.07	722.07	383.47	1273.52	1710.67	897.85
Niue	0.01	0.01	0.01	0.01	0.01	0.01	0.01	0.02	0.01	0.01	0.01	0.01	0.02	0.03	0.02	0.02	0.02	0.01
North Macedonia	10.12	13.72	7.04	13.73	18.56	9.59	10.74	14.36	7.50	13.75	18.61	9.77	20.86	28.12	14.60	27.49	36.90	19.37
Northern Mariana Islands	0.18	0.25	0.12	0.22	0.30	0.15	0.18	0.25	0.13	0.28	0.37	0.20	0.37	0.50	0.25	0.50	0.67	0.35
Norway	21.43	28.95	14.87	28.18	38.14	19.70	29.29	39.21	20.75	36.29	48.61	25.73	50.72	68.46	35.55	64.47	86.87	45.21
Oman	7.18	9.75	4.88	24.86	34.34	16.71	3.87	5.25	2.70	10.57	14.17	7.31	11.05	14.91	7.61	35.43	48.33	24.02
Pakistan	202.66	274.14	141.34	445.99	610.93	309.14	268.81	364.39	189.70	592.05	801.64	416.28	471.47	635.04	332.75	1038.04	1413.03	725.36
Palau	0.06	0.08	0.04	0.10	0.13	0.07	0.07	0.10	0.05	0.11	0.15	0.08	0.13	0.17	0.09	0.21	0.28	0.15
Palestine	5.23	7.04	3.59	15.57	21.19	10.82	5.30	7.15	3.74	14.39	19.29	10.09	10.53	14.17	7.32	29.96	39.89	20.91
Panama	5.60	7.52	3.87	12.05	16.24	8.37	9.67	13.06	6.72	19.85	26.80	13.91	15.27	20.41	10.61	31.90	42.73	22.25
Papua New Guinea	12.76	17.12	8.84	33.26	44.65	23.34	15.36	20.57	10.85	40.29	54.01	28.43	28.11	37.42	19.71	73.54	98.48	51.58
Paraguay	11.80	15.79	8.18	25.16	34.07	17.45	12.88	17.27	9.12	29.12	39.40	20.37	24.68	33.27	17.28	54.28	73.90	37.93
Peru	50.36	67.94	35.25	103.76	139.87	73.37	61.72	83.04	43.34	129.63	174.40	91.41	112.08	150.19	78.26	233.39	313.91	164.43
Philippines	203.81	273.08	141.87	398.39	530.76	279.20	281.65	377.88	197.98	583.78	777.92	412.02	485.47	645.98	339.69	982.18	1303.80	690.24
Poland	206.77	277.38	144.67	257.35	349.45	182.16	275.02	369.46	192.75	316.42	427.17	223.58	481.78	645.13	336.78	573.78	774.28	404.88
Portugal	52.57	71.03	36.36	63.36	85.82	44.75	85.23	114.57	60.61	110.99	147.85	78.89	137.80	186.14	96.84	174.35	234.90	124.04
Puerto Rico	10.24	13.68	7.22	12.71	17.05	9.02	15.79	21.29	11.08	20.76	27.91	14.81	26.02	34.95	18.37	33.48	44.89	23.86
Qatar	2.44	3.32	1.63	20.50	28.03	14.07	0.81	1.09	0.56	5.20	7.08	3.52	3.25	4.43	2.18	25.69	35.01	17.54
Republic of Korea	169.05	230.44	116.81	287.46	393.18	199.77	273.29	368.37	189.18	426.87	577.65	301.23	442.34	601.51	306.57	714.32	966.62	501.07
Republic of Moldova	18.04	24.27	12.63	19.03	25.61	13.30	26.26	35.04	18.49	26.93	35.89	18.73	44.31	58.92	31.27	45.96	61.58	32.04
Romania	146.57	197.40	103.05	137.53	186.88	96.50	169.04	228.99	120.36	159.66	215.20	113.55	315.61	425.51	225.14	297.19	403.49	210.12
Russian Federation	596.62	798.22	419.13	651.53	878.77	459.75	1158.14	1546.86	817.16	1298.52	1744.11	926.58	1754.76	2352.87	1235.40	1950.05	2632.31	1390.78
Rwanda	17.92	24.16	12.42	36.38	49.21	25.19	15.47	20.86	10.82	31.57	42.12	22.33	33.39	44.86	23.33	67.95	91.13	47.39

Saint Kitts and Nevis	0.11	0.14	0.08	0.20	0.28	0.14	0.16	0.21	0.11	0.28	0.38	0.20	0.27	0.36	0.19	0.49	0.66	0.34
Saint Lucia	0.33	0.44	0.23	0.62	0.83	0.43	0.50	0.66	0.35	0.89	1.20	0.63	0.83	1.10	0.58	1.50	2.03	1.06
Saint Vincent and the Grenadines	0.26	0.35	0.18	0.39	0.52	0.27	0.37	0.50	0.26	0.51	0.69	0.36	0.63	0.85	0.45	0.90	1.20	0.63
Samoa	0.58	0.78	0.41	0.82	1.10	0.57	0.71	0.95	0.50	1.02	1.37	0.72	1.29	1.72	0.91	1.85	2.47	1.30
San Marino	0.13	0.17	0.09	0.18	0.25	0.13	0.17	0.23	0.12	0.27	0.36	0.19	0.30	0.40	0.21	0.45	0.61	0.32
Sao Tome and Principe	0.27	0.36	0.19	0.55	0.74	0.38	0.27	0.36	0.19	0.51	0.69	0.35	0.54	0.72	0.38	1.05	1.42	0.74
Saudi Arabia	54.88	75.44	37.55	182.02	249.33	122.87	34.25	45.99	23.73	109.68	147.81	76.31	89.13	119.81	61.31	291.70	397.00	199.99
Senegal	16.51	22.21	11.67	37.02	49.68	25.88	15.97	21.27	11.16	36.49	49.40	25.51	32.48	43.39	22.81	73.51	98.40	51.59
Serbia	58.90	80.06	41.02	62.52	83.80	44.07	61.44	82.93	42.96	64.48	86.81	45.18	120.34	162.44	84.30	127.00	170.74	89.14
Seychelles	0.24	0.32	0.16	0.44	0.59	0.31	0.31	0.41	0.22	0.50	0.67	0.35	0.54	0.72	0.38	0.94	1.26	0.65
Sierra Leone	9.87	13.44	7.00	21.84	29.67	15.31	9.47	12.79	6.62	20.42	27.31	14.36	19.34	26.08	13.65	42.26	56.98	29.64
Singapore	10.21	13.89	7.00	24.08	33.33	16.88	15.94	21.53	10.98	33.18	45.12	23.27	26.15	35.51	17.93	57.27	78.64	40.46
Slovakia	32.26	43.40	22.55	39.04	53.19	27.51	32.17	43.49	22.60	39.45	53.31	27.74	64.43	86.44	45.14	78.49	106.44	55.51
Slovenia	12.10	16.32	8.45	15.40	20.74	10.90	12.50	16.57	8.81	14.94	20.10	10.60	24.60	32.79	17.24	30.34	40.79	21.20
Solomon Islands	0.96	1.31	0.68	2.08	2.82	1.46	1.13	1.52	0.79	2.64	3.55	1.86	2.09	2.81	1.48	4.72	6.32	3.30
Somalia	17.17	23.35	11.85	49.07	66.56	34.30	13.19	17.58	9.22	37.36	49.79	26.37	30.36	40.82	21.05	86.43	115.88	60.30
South Africa	85.77	114.53	60.11	150.62	201.25	105.56	98.86	131.45	69.60	173.52	231.44	122.39	184.63	245.19	130.26	324.14	432.74	227.79
South Sudan	15.84	21.18	11.19	24.51	33.12	17.37	10.06	13.52	7.03	18.61	24.96	12.98	25.89	34.59	18.20	43.12	57.93	30.39
Spain	201.94	264.72	143.41	194.38	267.11	137.85	252.17	331.04	178.80	321.50	438.00	230.33	454.11	603.92	325.51	515.88	703.16	371.03
Sri Lanka	54.19	72.71	38.20	85.20	113.69	60.34	58.77	78.61	41.33	109.10	146.90	76.98	112.96	150.45	79.81	194.30	261.71	137.88
Sudan	55.91	75.94	38.91	123.82	166.64	85.83	52.14	69.92	36.40	113.59	151.58	79.44	108.05	145.89	75.33	237.41	316.34	164.63
Suriname	1.00	1.35	0.70	1.78	2.42	1.24	1.37	1.87	0.96	2.63	3.51	1.85	2.37	3.19	1.66	4.41	5.93	3.11
Sweden	43.98	59.38	30.94	54.95	74.57	38.59	62.86	83.66	44.69	73.79	99.19	52.76	106.84	143.06	75.35	128.74	173.43	90.98
Switzerland	54.97	71.83	39.24	58.71	79.27	40.95	76.09	98.88	55.20	80.49	108.71	56.61	131.06	171.33	94.06	139.21	187.98	98.01
Syrian Arab Republic	36.25	48.80	25.29	56.66	75.82	39.44	32.42	43.18	22.96	56.72	76.17	39.53	68.67	91.39	48.22	113.38	152.27	79.65
Taiwan (Province of China)	99.60	132.68	70.11	124.11	167.52	87.62	125.99	166.94	88.49	170.94	230.74	120.42	225.59	299.17	158.44	295.05	401.30	208.35

Tajikistan	13.69	18.33	9.59	28.10	37.97	19.59	17.17	23.06	12.02	32.66	44.00	22.77	30.87	41.22	21.64	60.77	81.77	42.39
Thailand	161.72	219.47	114.58	305.52	412.10	215.75	207.02	276.69	144.54	412.55	553.20	293.20	368.73	495.63	260.40	718.07	967.17	506.88
Timor-Leste	2.10	2.82	1.46	3.82	5.15	2.68	2.44	3.30	1.69	4.64	6.20	3.30	4.54	6.11	3.17	8.46	11.36	5.99
Togo	7.82	10.59	5.45	20.49	27.61	14.31	7.98	10.83	5.57	21.50	28.86	15.16	15.80	21.52	11.12	42.00	56.57	29.43
Tokelau	0.01	0.01	0.00	0.01	0.01	0.00	0.01	0.01	0.01	0.01	0.01	0.01	0.01	0.02	0.01	0.01	0.02	0.01
Tonga	0.31	0.42	0.22	0.37	0.51	0.26	0.42	0.57	0.30	0.52	0.70	0.37	0.74	0.98	0.52	0.90	1.20	0.63
Trinidad and Tobago	3.20	4.31	2.20	4.92	6.63	3.47	4.47	6.02	3.12	6.96	9.36	4.93	7.67	10.30	5.33	11.88	16.04	8.39
Tunisia	27.86	37.37	19.36	51.68	69.89	36.41	23.98	32.07	17.05	49.85	66.35	34.54	51.85	68.76	36.40	101.53	135.33	71.42
Turkey	305.73	411.37	211.13	455.55	614.93	315.53	232.99	311.84	163.04	419.03	566.98	296.58	538.72	723.18	378.48	874.59	1178.27	608.05
Turkmenistan	9.74	13.02	6.75	17.90	24.20	12.33	12.67	16.91	8.91	21.03	28.32	14.74	22.41	30.03	15.76	38.93	52.01	27.24
Tuvalu	0.03	0.04	0.02	0.05	0.07	0.03	0.05	0.07	0.03	0.06	0.08	0.04	0.08	0.11	0.06	0.11	0.15	0.08
Uganda	38.85	52.19	27.47	94.85	128.46	66.06	31.20	41.44	22.00	78.86	105.73	54.82	70.05	93.04	49.33	173.70	234.27	120.70
Ukraine	254.89	344.14	179.02	238.90	322.16	168.44	424.97	568.36	303.38	405.57	541.47	288.71	679.86	914.24	485.62	644.46	860.17	457.33
United Arab Emirates	9.21	12.44	6.20	70.22	97.06	47.09	3.01	4.02	2.08	18.81	25.96	12.65	12.22	16.40	8.33	89.03	122.76	60.32
United Kingdom	346.66	471.40	242.43	393.29	529.55	275.12	475.77	637.72	333.69	566.84	759.55	401.03	822.43	1108.53	575.42	960.13	1285.20	675.14
United Republic of Tanzania	60.81	81.73	42.74	143.05	191.15	99.16	50.51	67.62	36.09	116.17	155.12	80.73	111.32	149.11	78.78	259.22	347.39	181.00
United States of America	1968.13	2644.35	1386.47	2528.57	3343.90	1824.74	2536.73	3395.65	1788.83	3168.58	4132.01	2289.54	4504.86	6039.64	3168.68	5697.15	7474.69	4114.14
United States Virgin Islands	0.29	0.39	0.20	0.37	0.49	0.26	0.45	0.61	0.31	0.60	0.80	0.42	0.74	0.99	0.51	0.96	1.28	0.68
Uruguay	10.76	14.50	7.50	13.24	18.11	9.25	15.02	20.27	10.53	19.82	26.73	14.04	25.78	34.62	18.03	33.06	44.60	23.21
Uzbekistan	53.62	72.28	37.33	108.09	146.56	75.06	68.61	91.98	48.60	131.96	177.83	92.42	122.23	163.97	85.78	240.05	321.81	167.10
Vanuatu	0.50	0.67	0.35	1.14	1.52	0.79	0.59	0.80	0.41	1.41	1.90	0.99	1.09	1.47	0.77	2.55	3.40	1.79
Venezuela (Bolivarian Republic of)	38.34	51.54	26.58	76.21	103.06	52.83	78.65	106.73	53.91	145.45	196.05	101.32	117.00	157.34	80.56	221.66	297.37	153.43
Viet Nam	179.97	244.20	126.41	395.74	533.35	276.88	250.39	336.96	176.80	530.05	717.49	372.25	430.37	577.21	304.88	925.79	1242.25	648.66
Yemen	35.05	47.39	24.39	93.77	126.87	64.30	33.09	43.98	23.08	87.13	115.99	60.80	68.14	91.05	47.57	180.91	242.35	124.72
Zambia	15.89	21.34	11.21	44.89	60.75	31.28	11.88	15.77	8.40	33.60	44.89	23.28	27.76	36.85	19.66	78.48	104.11	54.69

Zimbabwe	22.55	30.32	15.84	38.08	51.59	26.53	20.65	27.58	14.42	39.29	53.34	27.66	43.20	58.36	30.26	77.37	104.79	54.13

Footnote: LBP: low back pain; YLDs: years lived with disability; UUI: upper uncertainty interval; LUI: lower uncertainty interval

Table S3 The number and point prevalence of LBP by provinces/regions in China (2019 and the 30-year change)

men

province-level administrative units	2019									30-year change*								
	Number (×1000)			All-age prevalence (per 100,000)			Age-standardized (per 100,000)			Number (%)			All-age prevalence (%)			Age-standardized (%)		
Anhui	1650.88	1896.10	1436.38	5375.6	6174.1	4677.2	4391.7	4952.8	3852.3	3.0	11.3	-4.7	0.4	8.4	-7.2	-30.5	-26.7	-34.3
Beijing	667.12	760.33	575.14	5297.4	6037.6	4567.0	4279.1	4863.1	3743.9	102.7	116.6	89.1	-6.1	0.3	-12.5	-27.5	-23.1	-31.7
Chongqing	699.22	799.44	608.98	5629.6	6436.4	4903.0	4404.2	5003.5	3872.9	47.8	61.3	37.3	-5.0	3.7	-11.8	-30.2	-26.2	-33.8
Fujian	1026.15	1181.05	892.92	5059.4	5823.2	4402.5	4310.3	4881.4	3765.2	29.2	38.6	20.4	1.0	8.4	-5.9	-30.5	-26.7	-34.6
Gansu	695.53	800.43	606.52	5232.6	6021.8	4563.0	4435.3	5035.4	3884.4	11.6	20.1	3.1	0.7	8.4	-7.0	-29.8	-25.7	-33.6
Guangdong	3553.70	4030.48	3120.95	5640.9	6397.7	4954.0	5027.8	5634.9	4444.5	112.6	126.9	99.5	11.3	18.8	4.5	-17.5	-12.2	-22.4
Guangxi	1218.76	1386.47	1062.73	4827.4	5491.6	4209.4	4383.6	4972.9	3849.3	5.8	13.7	-0.9	-5.3	1.8	-11.2	-30.7	-26.7	-34.6
Guizhou	883.72	1006.41	770.46	4999.7	5693.7	4358.9	4446.4	5039.1	3912.1	-0.1	6.9	-7.3	-2.4	4.5	-9.4	-30.6	-26.6	-34.4
Hainan	249.20	284.90	216.09	4883.3	5582.9	4234.3	4357.9	4947.4	3815.9	42.7	53.6	33.4	-0.8	6.8	-7.3	-30.8	-26.9	-34.5
Hebei	1974.74	2260.67	1721.20	5101.3	5840.0	4446.4	4418.3	4980.4	3876.4	11.0	19.0	3.1	-8.2	-1.6	-14.8	-30.2	-26.2	-34.1
Heilongjiang	1158.61	1340.63	1005.19	5876.1	6799.3	5098.0	4444.9	5033.6	3896.5	18.3	29.8	7.9	12.3	23.2	2.4	-29.4	-25.2	-33.3
Henan	2383.63	2723.40	2087.01	4888.5	5585.3	4280.1	4431.1	5028.1	3881.8	-1.2	5.7	-8.0	-9.2	-2.9	-15.5	-30.3	-26.3	-34.1
Hong Kong	260.24	296.85	228.44	7567.4	8631.7	6642.7	5419.9	6116.1	4732.2	41.0	52.6	29.8	20.9	30.8	11.3	-11.2	-6.3	-16.5
Hubei	1422.30	1636.02	1244.04	5064.1	5825.1	4429.4	4002.0	4563.9	3490.4	3.9	13.8	-5.5	5.8	15.9	-3.8	-29.0	-23.7	-34.6
Hunan	1825.94	2077.47	1593.05	5288.3	6016.8	4613.8	4410.9	5000.8	3854.8	2.2	9.9	-5.5	-4.2	3.1	-11.4	-30.2	-26.0	-33.9
Inner Mongolia	741.24	856.18	645.84	5545.5	6405.4	4831.8	4402.2	4984.5	3869.4	22.9	33.5	12.7	7.1	16.4	-1.7	-29.9	-25.4	-33.7
Jiangsu	2291.40	2629.74	2005.13	5523.0	6338.5	4833.0	4324.4	4885.5	3788.8	13.0	21.6	5.4	-3.8	3.5	-10.3	-30.3	-25.8	-34.3
Jiangxi	1187.01	1354.80	1039.37	4829.0	5511.6	4228.3	4348.9	4938.8	3835.6	17.1	25.5	9.4	-4.9	1.8	-11.2	-30.7	-26.6	-34.3
Jilin	839.70	950.90	745.19	6065.4	6868.6	5382.7	4590.3	5126.9	4095.2	32.4	45.1	20.7	24.9	36.8	13.9	-19.4	-13.3	-24.9
Liaoning	1327.22	1527.06	1157.07	5879.9	6765.2	5126.1	4385.3	4962.5	3854.7	10.9	20.5	1.5	2.8	11.7	-5.9	-29.7	-25.3	-34.0
Macao	17.81	20.35	15.51	5083.1	5808.3	4425.0	4262.6	4812.4	3745.1	93.1	107.2	79.8	-4.6	2.4	-11.2	-28.5	-24.5	-32.8
Ningxia	173.90	199.12	150.79	4811.6	5509.4	4172.1	4345.9	4919.7	3800.5	48.6	59.5	37.8	1.2	8.7	-6.1	-30.6	-26.7	-34.3

women

province-level administrative units	2019									30-year change*								
	Number (×1000)			All-age prevalence (per 100,000)			Age-standardized (per 100,000)			Number (%)			All-age prevalence (%)			Age-standardized (%)		
Anhui	2520.06	2914.85	2200.73	8272.3	9568.2	7224.1	6211.6	7051.3	5472.6	16.0	25.9	6.5	6.1	15.2	-2.6	-29.4	-25.1	-33.4
Beijing	945.30	1078.40	830.38	7849.8	8955.0	6895.5	6053.4	6834.8	5349.3	110.0	123.7	95.6	-5.4	0.8	-11.9	-26.6	-22.6	-30.6
Chongqing	1105.85	1257.81	965.87	8734.3	9934.5	7628.7	6211.3	7000.7	5437.6	78.2	93.5	63.3	3.7	12.6	-4.9	-29.5	-25.2	-33.7
Fujian	1463.11	1670.17	1284.79	7615.1	8692.8	6687.0	6077.2	6863.3	5372.9	34.6	45.3	24.1	4.8	13.1	-3.3	-29.7	-25.8	-33.6
Gansu	1019.71	1169.03	894.28	7830.2	8976.9	6867.1	6251.6	7058.7	5518.0	25.6	36.6	15.8	7.3	16.7	-1.1	-29.0	-24.9	-33.4
Guangdong	4318.91	4869.12	3843.11	7747.3	8734.3	6893.8	6511.9	7314.1	5799.7	98.2	114.2	84.3	11.4	20.4	3.6	-17.0	-11.2	-22.0
Guangxi	1783.10	2041.89	1567.25	7422.8	8500.1	6524.3	6190.5	7013.2	5451.1	15.5	23.6	7.6	-1.5	5.4	-8.2	-30.0	-26.0	-34.1
Guizhou	1311.89	1495.65	1160.87	7597.9	8662.1	6723.3	6284.6	7112.8	5561.0	11.3	20.0	3.1	2.9	10.9	-4.6	-29.6	-25.7	-33.5
Hainan	329.92	376.49	290.70	7295.5	8325.4	6428.2	6171.7	6980.3	5457.0	36.9	45.8	28.0	-1.6	4.8	-8.0	-29.7	-25.5	-33.5
Hebei	2977.24	3409.07	2604.26	7916.7	9064.9	6924.9	6212.6	7022.2	5464.1	22.9	32.7	13.6	-0.2	7.8	-7.8	-29.6	-25.4	-33.4
Heilongjiang	1759.36	2037.40	1538.48	9019.4	10444.8	7887.0	6285.8	7099.9	5561.5	36.6	50.7	23.8	24.1	36.9	12.5	-28.5	-24.4	-32.5
Henan	3788.64	4341.79	3330.72	7736.4	8866.0	6801.4	6249.4	7046.4	5506.4	12.1	20.3	3.3	-2.3	4.9	-10.0	-29.6	-25.5	-33.7
Hong Kong	412.81	472.36	365.51	10156.6	11621.8	8992.8	6893.5	7819.1	6094.3	70.5	83.2	58.9	17.9	26.7	9.9	-15.2	-10.5	-19.8
Hubei	1994.06	2293.88	1734.70	7279.2	8373.7	6332.5	5256.3	5935.3	4596.6	15.2	25.6	5.2	12.6	22.8	2.8	-28.5	-23.6	-33.7
Hunan	2716.86	3088.21	2397.13	8158.9	9274.1	7198.8	6235.0	7015.7	5510.4	16.5	25.6	7.6	4.3	12.4	-3.7	-29.4	-24.7	-33.1
Inner Mongolia	1026.94	1176.63	897.42	8331.9	9546.4	7281.0	6202.2	6985.8	5463.2	35.9	47.9	24.7	18.2	28.7	8.5	-29.3	-25.3	-33.1
Jiangsu	3564.58	4087.88	3116.65	8697.3	9974.1	7604.3	6132.6	6913.3	5403.8	24.1	33.4	15.6	2.8	10.5	-4.2	-29.2	-25.3	-32.9
Jiangxi	1733.10	1996.05	1518.97	7406.9	8530.7	6491.7	6159.4	7001.4	5418.7	27.0	37.0	17.5	0.9	8.8	-6.7	-29.7	-25.3	-33.6
Jilin	1002.77	1171.06	860.41	7372.0	8609.2	6325.4	5074.9	5800.5	4406.0	30.1	44.5	15.4	18.6	31.8	5.3	-30.9	-25.8	-35.5
Liaoning	2080.37	2402.49	1823.08	9296.5	10736.0	8146.8	6211.0	7048.7	5481.2	29.1	41.6	18.2	15.2	26.4	5.5	-28.6	-24.7	-32.5
Macao	28.75	32.95	25.35	7730.8	8858.7	6816.3	6033.0	6822.5	5321.2	101.9	119.0	85.6	-0.8	7.6	-8.8	-27.6	-22.9	-31.5
Ningxia	242.02	277.09	211.55	7012.9	8029.1	6130.2	6142.9	6935.8	5458.3	59.2	71.7	46.4	7.1	15.6	-1.5	-29.5	-25.2	-33.5

Both

province-level administrative units	2019									30-year change*								
	Number (×1000)			All-age prevalence (per 100,000)			Age-standardized (per 100,000)			Number (%)			All-age prevalence (%)			Age-standardized (%)		
Anhui	4170.94	4767.41	3660.34	6818.1	7793.2	5983.5	5313.9	5989.8	4697.1	10.5	18.5	2.3	4.4	11.9	-3.3	-29.6	-26.5	-32.7
Beijing	1612.42	1836.48	1416.63	6545.1	7454.5	5750.3	5171.2	5814.8	4562.9	106.9	118.0	95.6	-5.4	-0.4	-10.6	-26.9	-23.6	-29.8
Chongqing	1805.07	2050.28	1579.56	7196.8	8174.4	6297.7	5328.7	5991.6	4685.6	65.0	77.5	53.5	1.0	8.7	-6.0	-29.3	-26.3	-32.3
Fujian	2489.26	2848.69	2179.40	6302.7	7212.8	5518.1	5197.7	5840.6	4591.8	32.3	41.3	23.9	3.3	10.3	-3.3	-30.0	-27.0	-33.0
Gansu	1715.24	1959.99	1508.57	6518.1	7448.2	5732.8	5353.2	6037.1	4715.0	19.5	28.4	11.7	5.0	12.8	-1.9	-28.9	-25.8	-32.2
Guangdong	7872.61	8897.57	6979.41	6629.8	7493.0	5877.6	5766.1	6450.6	5146.2	104.5	116.9	93.6	10.8	17.5	4.9	-17.4	-13.1	-21.3
Guangxi	3001.86	3427.49	2645.06	6092.8	6956.7	5368.6	5305.9	5999.3	4692.2	11.3	18.0	5.0	-2.6	3.3	-8.1	-29.9	-26.8	-32.8
Guizhou	2195.62	2498.61	1936.30	6283.6	7150.7	5541.5	5386.9	6080.5	4769.6	6.4	12.9	-0.4	1.2	7.4	-5.2	-29.6	-26.5	-32.4
Hainan	579.12	661.17	507.63	6016.6	6869.0	5273.8	5239.8	5913.5	4606.6	39.4	47.3	31.9	-1.6	4.0	-6.8	-30.5	-27.4	-33.4
Hebei	4951.98	5661.40	4339.76	6488.7	7418.2	5686.4	5340.3	6008.4	4702.9	17.9	25.3	10.9	-3.4	2.7	-9.2	-29.4	-26.2	-32.2
Heilongjiang	2917.97	3367.79	2554.10	7439.3	8586.1	6511.6	5392.2	6091.2	4749.4	28.7	40.4	17.9	19.6	30.4	9.5	-28.2	-25.1	-31.0
Henan	6172.27	7017.17	5448.48	6315.5	7180.0	5574.9	5381.5	6062.8	4755.8	6.6	13.3	-0.2	-4.6	1.4	-10.7	-29.4	-26.5	-32.6
Hong Kong	673.05	766.88	597.35	8969.9	10220.3	7961.0	6185.2	6978.5	5439.0	57.7	68.1	48.0	21.1	29.1	13.6	-13.0	-9.3	-16.3
Hubei	3416.37	3914.38	2991.84	6157.9	7055.5	5392.7	4638.5	5250.2	4077.7	10.2	19.0	0.9	10.1	18.8	0.7	-28.5	-24.2	-33.4
Hunan	4542.80	5147.73	4002.29	6697.6	7589.5	5900.7	5330.4	6004.1	4705.3	10.3	17.1	3.0	1.1	7.4	-5.5	-29.3	-25.9	-32.3
Inner Mongolia	1768.18	2021.84	1551.41	6882.2	7869.5	6038.5	5304.4	5962.7	4701.1	30.1	39.4	20.5	13.3	21.4	5.0	-28.8	-25.9	-31.8
Jiangsu	5855.97	6679.88	5150.37	7100.4	8099.4	6244.9	5246.4	5929.1	4642.9	19.5	26.8	12.3	0.4	6.5	-5.7	-29.6	-26.4	-32.7
Jiangxi	2920.11	3346.19	2565.27	6086.2	6974.2	5346.6	5265.0	5941.7	4672.2	22.8	30.2	15.1	-1.3	4.6	-7.5	-29.9	-26.9	-32.8
Jilin	1842.47	2111.69	1612.39	6712.9	7693.8	5874.7	4841.9	5443.7	4271.9	31.1	43.3	19.1	21.7	33.0	10.5	-25.5	-20.7	-29.7
Liaoning	3407.59	3901.83	2998.38	7580.8	8680.3	6670.5	5323.1	5996.6	4703.8	21.3	30.2	12.9	10.4	18.5	2.7	-28.6	-25.7	-31.4
Macao	46.57	52.85	41.12	6446.4	7315.8	5692.0	5197.3	5848.9	4585.7	98.4	111.9	85.2	-2.3	4.4	-8.8	-27.9	-24.7	-30.9
Ningxia	415.91	474.42	364.45	5886.8	6714.9	5158.4	5252.7	5906.5	4651.0	54.6	64.5	45.0	4.7	11.4	-1.8	-29.4	-26.4	-32.5

Qinghai	160.99	184.95	139.77	4863.0	5586.8	4222.2	4366.7	4954.8	3853.5	40.8	52.6	30.2	1.8	10.3	-5.9	-29.9	-26.2	-33.9
Shaanxi	988.79	1121.56	871.13	4913.8	5573.6	4329.1	4092.4	4615.7	3632.1	16.7	26.7	7.1	1.3	9.9	-7.1	-28.0	-22.2	-33.3
Shandong	2590.36	2951.13	2281.03	4976.1	5669.1	4381.9	4127.3	4689.5	3621.9	16.7	27.2	7.5	-1.6	7.2	-9.3	-27.5	-21.5	-32.8
Shanghai	655.62	785.39	542.82	4453.5	5335.0	3687.2	3496.2	4141.9	2927.2	50.4	62.7	37.1	-27.3	-21.3	-33.7	-39.7	-34.8	-44.7
Shanxi	1010.93	1157.72	881.85	5162.4	5912.1	4503.3	4349.1	4921.9	3820.1	21.6	31.0	13.1	-4.0	3.4	-10.7	-30.5	-26.7	-34.3
Sichuan	2318.35	2652.60	2025.61	5580.9	6385.5	4876.2	4415.5	5006.3	3870.0	-29.7	-24.6	-35.0	-3.5	3.6	-10.7	-30.4	-26.1	-34.5
Tianjin	453.63	520.87	393.29	5269.9	6051.1	4568.9	4282.6	4845.1	3737.4	71.0	82.7	60.3	-7.6	-1.2	-13.3	-28.7	-24.3	-32.8
Tibet	75.82	87.49	65.27	4201.7	4848.3	3617.0	4340.0	4922.8	3807.1	40.9	50.5	31.8	-11.6	-5.6	-17.3	-30.3	-26.3	-34.2
Xinjiang	607.17	697.94	527.11	4800.2	5517.8	4167.2	4314.3	4895.7	3777.3	56.1	68.0	44.6	-1.6	5.8	-8.9	-29.8	-25.7	-33.8
Yunnan	1069.68	1250.60	905.60	4233.2	4949.2	3583.9	3767.3	4372.5	3216.8	19.7	31.5	8.5	-7.2	2.1	-15.8	-34.0	-28.9	-39.0
Zhejiang	1388.16	1646.43	1169.39	4368.5	5181.2	3680.0	3513.9	4132.5	2971.9	15.1	27.4	3.4	-18.9	-10.2	-27.1	-39.4	-33.7	-44.3
**China**	37567.53	42858.89	33023.98	5183.0	5913.0	4556.2	4328.8	4898.4	3824.3	16.0	21.5	10.5	-2.3	2.3	-7.0	-29.4	-27.1	-31.8

Qinghai	212.75	243.63	185.27	6973.5	7985.8	6072.8	6166.7	6977.1	5439.1	41.4	53.0	30.9	2.4	10.8	-5.2	-29.1	-25.1	-33.0
Shaanxi	1286.56	1480.11	1119.00	6781.7	7802.0	5898.5	5171.3	5880.9	4520.7	26.2	37.5	15.4	7.3	16.9	-1.9	-29.8	-24.3	-34.8
Shandong	3429.24	3965.26	2996.58	6749.5	7804.5	5897.9	4974.2	5693.7	4337.1	17.7	29.6	7.5	-1.7	8.2	-10.2	-32.6	-27.4	-37.9
Shanghai	901.65	1041.90	786.90	6569.8	7591.7	5733.7	4827.0	5517.6	4218.2	64.3	78.1	52.4	-18.7	-11.9	-24.6	-32.3	-26.8	-37.1
Shanxi	1444.57	1659.39	1267.46	7697.8	8842.4	6753.9	6148.0	6992.0	5431.5	33.6	43.5	23.9	1.3	8.8	-6.0	-29.5	-25.2	-33.4
Sichuan	3577.03	4119.17	3148.40	8627.5	9935.1	7593.7	6258.1	7078.5	5527.2	-18.1	-11.0	-24.5	4.6	13.7	-3.6	-29.2	-24.7	-33.2
Tianjin	567.59	650.88	498.03	8122.2	9314.1	7126.8	6050.4	6840.3	5336.4	54.7	66.7	43.5	-0.9	6.7	-8.1	-27.9	-23.4	-32.2
Tibet	100.49	114.21	87.97	5995.4	6814.1	5248.6	6148.0	6934.3	5399.6	24.8	33.1	17.8	-16.4	-10.9	-21.1	-29.2	-24.8	-33.5
Xinjiang	803.74	919.86	706.76	6777.5	7756.7	5959.7	6102.9	6876.8	5422.1	63.6	76.2	51.7	2.5	10.4	-4.9	-28.9	-24.8	-32.8
Yunnan	1403.04	1621.75	1211.96	5864.9	6779.1	5066.2	4954.1	5696.4	4331.6	23.0	34.7	12.7	-5.1	3.9	-13.0	-33.2	-27.7	-38.5
Zhejiang	1919.90	2309.34	1602.59	6402.4	7701.1	5344.3	4777.4	5668.5	4011.3	24.0	36.6	10.6	-13.1	-4.3	-22.5	-38.4	-33.6	-43.6
**China**	53771.90	61618.27	47456.18	7708.9	8833.8	6803.5	5915.8	6670.1	5240.2	25.3	31.9	18.2	3.0	8.4	-2.8	-29.4	-27.3	-31.4

Qinghai	373.73	426.31	324.89	5875.2	6701.8	5107.4	5268.2	5947.5	4657.9	41.2	50.6	32.1	2.1	8.9	-4.5	-29.3	-26.3	-32.5
Shaanxi	2275.35	2592.09	2009.45	5820.3	6630.5	5140.1	4635.4	5245.5	4106.3	21.9	31.5	12.8	4.7	13.0	-3.1	-28.7	-23.7	-33.1
Shandong	6019.60	6896.05	5289.65	5852.0	6704.1	5142.4	4574.7	5170.9	4005.2	17.3	27.2	8.5	-1.6	6.8	-8.9	-30.2	-25.5	-34.7
Shanghai	1557.27	1822.84	1343.06	5474.5	6408.1	4721.5	4150.3	4788.4	3572.8	58.1	68.8	48.5	-22.7	-17.5	-27.4	-35.9	-31.6	-40.0
Shanxi	2455.50	2803.19	2155.66	6403.1	7309.8	5621.2	5254.1	5925.4	4652.3	28.4	36.4	20.8	-0.6	5.6	-6.4	-29.5	-26.5	-32.4
Sichuan	5895.38	6750.13	5190.01	7102.7	8132.5	6252.9	5358.8	6025.3	4734.7	-23.1	-18.1	-28.4	1.9	8.5	-5.1	-29.2	-25.9	-32.2
Tianjin	1021.22	1167.51	898.52	6547.9	7486.0	5761.2	5140.3	5807.0	4516.8	61.5	70.9	53.2	-5.5	0.0	-10.3	-28.6	-25.4	-31.5
Tibet	176.31	199.81	154.71	5065.4	5740.6	4444.7	5270.7	5928.8	4646.6	31.2	38.0	25.4	-15.0	-10.6	-18.8	-29.9	-26.8	-33.0
Xinjiang	1410.90	1605.05	1237.18	5757.0	6549.1	5048.1	5189.8	5807.9	4603.4	60.3	70.1	51.1	0.7	6.9	-5.1	-28.8	-25.8	-31.7
Yunnan	2472.72	2853.62	2129.28	5026.7	5801.0	4328.5	4380.9	5009.2	3816.6	21.5	30.8	12.3	-6.0	1.2	-13.2	-33.2	-29.0	-37.9
Zhejiang	3308.06	3939.43	2778.50	5356.0	6378.2	4498.6	4147.5	4876.0	3494.8	20.1	30.2	9.1	-15.6	-8.4	-23.3	-38.6	-34.6	-43.0
**China**	91339.43	104119.89	80527.99	6421.7	7320.3	5661.6	5134.7	5787.0	4548.5	21.3	27.2	15.0	0.9	5.8	-4.3	-29.1	-27.2	-30.9

Footnote: LBP: low back pain;

* Changes are the total mean values at 2019 compared with 1990

For each indicators, the 1st column was mean value and the 2nd and 3rd were upper uncertainty interval (UUI) and lower uncertainty interval (LUI) respectively.

Table S4 The number and point prevalence of of YLDs caused by LBP by provinces/regions in China (2019 and the 30-year change)

province-level administrative units	men 2019									men 30-year change*								
	Number (×1000)			All-age prevalence (per 100,000)			Age-standardized (per 100,000)			Number (%)			All-age prevalence (%)			Age-standardized (%)		
Anhui	189.12	258.70	132.66	615.8	842.4	432.0	501.6	684.3	353.1	2.6	11.8	-6.1	0.0	8.9	-8.5	-30.2	-25.9	-34.6
Beijing	76.60	104.02	52.96	608.3	826.0	420.6	487.1	655.4	342.3	102.6	119.4	87.6	-6.2	1.6	-13.1	-27.3	-22.4	-32.2
Chongqing	79.44	107.37	55.92	639.5	864.5	450.2	501.3	664.7	353.3	46.0	59.8	34.1	-6.1	2.7	-13.8	-30.0	-25.2	-34.4
Fujian	118.07	160.81	82.21	582.2	792.9	405.3	491.8	664.0	341.5	29.3	39.9	18.9	1.1	9.4	-7.0	-30.3	-25.7	-34.8
Gansu	79.82	108.91	55.44	600.5	819.3	417.1	505.8	686.7	354.0	11.5	21.2	2.6	0.6	9.3	-7.5	-29.5	-24.6	-34.0
Guangdong	408.57	550.34	282.72	648.5	873.6	448.8	572.1	772.4	402.2	113.1	129.4	98.2	11.6	20.1	3.8	-17.2	-11.4	-22.8
Guangxi	139.83	189.97	98.32	553.9	752.5	389.4	500.0	668.5	351.5	5.8	14.1	-1.4	-5.2	2.2	-11.7	-30.4	-25.7	-34.8
Guizhou	100.94	137.75	70.22	571.0	779.3	397.3	505.7	685.1	354.6	-0.2	7.5	-7.6	-2.5	5.1	-9.7	-30.3	-26.0	-34.6
Hainan	28.60	38.99	19.94	560.4	764.1	390.7	495.8	672.2	348.3	42.9	55.2	31.9	-0.7	7.9	-8.3	-30.6	-25.8	-35.1
Hebei	226.64	309.48	159.17	585.5	799.5	411.2	503.8	679.3	354.9	10.7	19.4	2.4	-8.5	-1.3	-15.3	-30.1	-25.8	-34.5
Heilongjiang	133.03	181.77	93.28	674.7	921.9	473.1	506.3	682.1	357.6	18.2	30.5	7.2	12.2	23.9	1.8	-29.1	-24.6	-33.4
Henan	273.13	370.82	191.05	560.1	760.5	391.8	505.5	681.3	356.3	-1.6	6.2	-9.1	-9.6	-2.5	-16.5	-30.2	-25.6	-34.7
Hong Kong	29.49	40.01	20.72	857.6	1163.4	602.6	619.1	842.6	435.8	38.7	51.2	27.3	19.0	29.7	9.2	-11.2	-5.4	-16.5
Hubei	163.09	221.89	114.39	580.7	790.1	407.3	456.9	616.3	322.4	3.6	14.3	-6.2	5.5	16.4	-4.4	-28.7	-22.8	-34.9
Hunan	208.67	282.79	146.73	604.4	819.0	425.0	502.4	679.5	350.3	1.7	9.9	-6.3	-4.6	3.1	-12.1	-30.1	-25.4	-34.5
Inner Mongolia	85.22	117.85	58.76	637.6	881.7	439.6	501.3	674.2	351.8	22.9	34.5	11.8	7.1	17.3	-2.5	-29.7	-24.8	-34.0
Jiangsu	261.79	353.68	182.54	631.0	852.5	440.0	493.1	671.5	347.8	12.3	21.5	3.8	-4.4	3.5	-11.6	-30.1	-25.0	-34.6
Jiangxi	136.31	183.74	95.53	554.5	747.5	388.6	496.3	665.3	348.3	17.2	26.6	8.4	-4.8	2.8	-12.0	-30.4	-26.0	-34.4
Jilin	96.49	130.09	67.58	696.9	939.6	488.1	524.2	709.9	372.3	32.3	46.6	19.6	24.8	38.3	12.9	-19.0	-12.6	-24.9
Liaoning	151.97	207.76	105.73	673.2	920.4	468.4	500.1	679.7	352.3	10.2	20.4	0.1	2.1	11.6	-7.2	-29.6	-24.7	-34.6
Macao	2.05	2.79	1.43	586.0	796.8	407.5	487.8	657.0	341.1	93.1	109.7	77.6	-4.6	3.6	-12.3	-28.2	-23.7	-33.1
Ningxia	20.07	27.53	13.98	555.4	761.6	386.9	496.5	681.6	347.0	48.9	61.1	36.6	1.5	9.8	-6.9	-30.5	-25.9	-34.9

province-level administrative units	women 2019									women 30-year change*								
	Number (×1000)			All-age prevalence (per 100,000)			Age-standardized (per 100,000)			Number (%)			All-age prevalence (%)			Age-standardized (%)		
Anhui	282.63	380.49	200.03	927.8	1249.0	656.6	697.4	940.3	494.3	15.9	26.4	5.3	6.0	15.6	-3.7	-29.0	-24.1	-33.6
Beijing	105.98	142.08	74.88	880.1	1179.9	621.8	677.8	907.5	479.1	108.8	124.5	92.9	-5.9	1.1	-13.1	-26.4	-22.0	-30.7
Chongqing	123.05	163.39	87.12	971.8	1290.5	688.1	694.9	929.0	491.7	76.2	92.7	61.5	2.5	12.2	-6.0	-29.4	-25.0	-33.9
Fujian	165.01	223.83	115.07	858.8	1165.0	598.9	683.5	923.7	479.3	34.7	46.3	24.0	4.9	13.9	-3.4	-29.5	-25.2	-34.0
Gansu	114.34	154.59	81.53	878.0	1187.1	626.1	698.5	938.1	502.2	25.0	37.0	14.3	6.7	17.0	-2.3	-28.9	-24.2	-33.5
Guangdong	485.17	649.85	342.33	870.3	1165.7	614.1	729.0	983.5	517.2	98.2	114.7	83.2	11.4	20.7	3.0	-16.9	-10.8	-22.4
Guangxi	199.85	270.07	141.44	832.0	1124.3	588.8	694.6	932.4	492.1	15.2	24.1	6.6	-1.7	5.9	-9.1	-29.8	-25.4	-34.2
Guizhou	146.55	195.40	104.84	848.8	1131.6	607.2	702.0	933.7	500.4	10.9	20.1	2.8	2.6	11.1	-4.9	-29.3	-24.7	-33.4
Hainan	37.03	50.28	26.18	818.9	1111.9	578.9	690.6	940.5	491.3	37.1	47.1	27.5	-1.5	5.7	-8.4	-29.5	-24.9	-33.8
Hebei	333.95	452.96	233.74	888.0	1204.4	621.5	695.8	938.1	497.0	22.0	32.0	12.4	-1.0	7.1	-8.8	-29.8	-25.5	-33.8
Heilongjiang	197.37	266.16	139.94	1011.8	1364.5	717.4	702.9	943.5	500.0	35.5	50.7	22.3	23.1	36.9	11.1	-28.3	-23.7	-32.6
Henan	424.94	573.20	302.12	867.7	1170.5	616.9	700.8	944.9	496.4	11.6	20.8	3.0	-2.7	5.2	-10.2	-29.6	-25.2	-34.0
Hong Kong	45.99	62.22	32.43	1131.5	1530.8	798.0	774.2	1038.9	545.5	68.5	82.7	56.1	16.5	26.3	7.9	-15.4	-9.8	-20.4
Hubei	223.99	304.75	158.84	817.7	1112.5	579.8	590.2	797.0	419.1	15.0	26.1	4.1	12.4	23.2	1.8	-28.3	-22.8	-33.7
Hunan	303.66	406.88	214.34	911.9	1221.9	643.7	697.8	927.2	492.7	16.0	26.1	6.0	3.9	12.9	-5.1	-29.1	-23.9	-33.6
Inner Mongolia	115.59	157.66	81.79	937.8	1279.2	663.6	694.5	929.6	493.1	34.9	47.3	22.9	17.3	28.1	6.9	-29.3	-24.6	-33.7
Jiangsu	397.04	530.56	279.84	968.7	1294.5	682.8	686.7	920.3	485.4	23.1	33.0	14.1	2.0	10.2	-5.4	-29.0	-24.7	-33.1
Jiangxi	195.20	263.83	136.46	834.3	1127.5	583.2	691.7	930.7	484.4	27.3	37.5	17.2	1.2	9.3	-6.9	-29.3	-24.9	-33.6
Jilin	112.70	154.06	80.06	828.6	1132.6	588.6	568.8	770.4	403.9	28.9	44.4	13.7	17.6	31.7	3.7	-30.8	-25.2	-35.7
Liaoning	232.58	313.15	165.01	1039.3	1399.4	737.4	695.7	930.7	492.5	27.5	40.4	16.0	13.8	25.3	3.6	-28.6	-24.2	-33.1
Macao	3.24	4.36	2.28	871.4	1172.4	613.6	678.1	901.5	481.4	101.9	121.9	83.9	-0.8	9.0	-9.6	-27.7	-22.4	-32.1
Ningxia	27.33	37.08	19.34	791.8	1074.4	560.4	688.3	923.6	492.0	58.4	71.9	45.1	6.6	15.7	-2.3	-29.6	-25.0	-34.1

province-level administrative units	Both 2019									Both 30-year change*								
	Number (×1000)			All-age prevalence (per 100,000)			Age-standardized (per 100,000)			Number (%)			All-age prevalence (%)			Age-standardized (%)		
Anhui	471.75	639.04	333.12	771.2	1044.6	544.5	600.4	811.3	425.0	10.2	18.8	2.0	4.1	12.3	-3.7	-29.2	-25.5	-32.5
Beijing	182.58	246.52	128.25	741.1	1000.6	520.6	582.6	777.4	410.9	106.2	118.8	94.3	-5.8	0.0	-11.2	-26.7	-23.3	-29.9
Chongqing	202.48	271.39	143.01	807.3	1082.0	570.2	600.0	807.5	425.4	63.0	76.3	51.3	-0.2	7.9	-7.4	-29.2	-25.9	-32.4
Fujian	283.08	384.33	196.90	716.8	973.1	498.5	587.6	789.5	411.7	32.4	42.1	23.5	3.4	10.9	-3.6	-29.7	-26.4	-33.2
Gansu	194.16	260.55	138.00	737.8	990.1	524.4	602.8	807.8	430.4	19.1	28.3	10.6	4.6	12.7	-2.9	-28.7	-25.3	-32.1
Guangdong	893.74	1202.96	622.87	752.6	1013.1	524.5	649.8	872.0	459.1	104.7	117.9	93.5	10.9	18.0	4.8	-17.2	-12.8	-21.4
Guangxi	339.68	457.62	239.31	689.4	928.8	485.7	598.9	803.3	426.4	11.1	17.8	4.6	-2.7	3.0	-8.5	-29.7	-26.3	-32.9
Guizhou	247.49	331.82	174.53	708.3	949.6	499.5	605.7	808.3	427.5	6.1	13.4	-0.9	1.0	7.9	-5.7	-29.3	-26.1	-32.5
Hainan	65.63	89.19	46.05	681.9	926.6	478.4	590.0	793.2	417.8	39.6	48.0	31.5	-1.4	4.5	-7.2	-30.3	-27.0	-33.7
Hebei	560.59	763.72	393.17	734.5	1000.7	515.2	602.0	811.3	427.8	17.2	25.4	9.8	-4.0	2.7	-10.0	-29.5	-26.3	-32.8
Heilongjiang	330.40	448.53	233.83	842.3	1143.5	596.2	607.0	815.7	431.4	28.0	40.4	17.0	18.9	30.5	8.7	-28.0	-24.7	-31.1
Henan	698.07	944.48	494.20	714.3	966.4	505.7	607.1	820.5	430.6	6.1	13.5	-1.4	-5.1	1.6	-11.8	-29.4	-25.9	-32.9
Hong Kong	75.48	101.85	53.15	1006.0	1357.4	708.4	699.4	944.8	494.5	55.4	66.3	45.8	19.3	27.7	11.9	-13.0	-8.9	-16.8
Hubei	387.08	529.28	273.63	697.7	954.0	493.2	524.2	708.0	370.2	9.9	19.2	0.2	9.7	19.0	0.0	-28.3	-23.7	-33.2
Hunan	512.33	690.24	361.45	755.3	1017.7	532.9	600.5	804.2	425.0	9.7	17.4	1.8	0.6	7.7	-6.7	-29.1	-25.5	-32.4
Inner Mongolia	200.81	271.41	140.88	781.6	1056.4	548.4	597.7	806.4	422.9	29.5	39.7	19.5	12.8	21.6	4.1	-28.8	-25.4	-32.1
Jiangsu	658.83	892.19	462.53	798.8	1081.8	560.8	591.4	799.1	420.3	18.6	26.7	11.1	-0.4	6.4	-6.7	-29.4	-26.0	-32.8
Jiangxi	331.51	449.21	232.48	690.9	936.2	484.5	594.8	803.0	421.7	23.0	31.1	15.1	-1.2	5.3	-7.5	-29.6	-26.2	-32.8
Jilin	209.19	282.72	149.21	762.2	1030.1	543.6	547.3	738.8	389.5	30.4	43.7	18.1	21.0	33.3	9.6	-25.3	-20.3	-29.6
Liaoning	384.55	518.75	272.32	855.5	1154.1	605.8	600.2	807.4	423.8	20.1	30.0	10.9	9.2	18.3	0.9	-28.6	-25.1	-32.1
Macao	5.29	7.14	3.72	732.9	988.2	515.4	588.1	787.2	415.5	98.4	114.0	83.9	-2.3	5.4	-9.4	-27.8	-24.1	-31.1
Ningxia	47.40	64.21	33.26	670.9	908.8	470.8	592.9	794.9	422.4	54.3	64.7	43.7	4.5	11.6	-2.7	-29.4	-26.2	-32.9

Qinghai	18.61	25.63	12.85	562.1	774.1	388.2	498.5	672.2	351.5	41.1	54.1	29.3	2.0	11.4	-6.5	-29.8	-25.4	-34.5
Shaanxi	113.81	155.58	79.59	565.6	773.1	395.5	467.3	626.9	330.6	16.7	28.3	6.2	1.3	11.3	-7.9	-27.8	-21.6	-33.8
Shandong	297.54	403.53	209.72	571.6	775.2	402.9	471.8	644.2	333.0	16.4	27.8	6.2	-1.9	7.8	-10.4	-27.3	-20.4	-33.2
Shanghai	75.23	104.15	51.63	511.0	707.4	350.7	398.5	546.3	280.3	50.6	64.2	36.6	-27.2	-20.6	-34.0	-39.4	-34.1	-44.8
Shanxi	116.29	158.62	80.51	593.8	810.0	411.1	496.0	669.1	347.1	21.9	32.0	12.6	-3.7	4.3	-11.1	-30.2	-25.7	-34.7
Sichuan	264.09	359.83	184.44	635.7	866.2	444.0	501.8	682.7	354.8	-30.3	-24.7	-35.8	-4.3	3.5	-11.8	-30.4	-25.5	-34.8
Tianjin	52.36	71.84	36.39	608.3	834.6	422.8	489.0	662.1	343.5	71.4	83.7	59.6	-7.4	-0.7	-13.7	-28.5	-23.7	-33.3
Tibet	8.77	11.97	6.06	486.0	663.4	335.7	494.8	662.8	347.0	42.2	53.2	32.3	-10.8	-3.8	-17.0	-29.8	-25.5	-34.4
Xinjiang	70.05	94.79	48.03	553.8	749.4	379.7	492.0	662.1	344.9	56.9	70.3	43.9	-1.2	7.3	-9.3	-29.6	-25.0	-34.2
Yunnan	122.46	171.28	84.83	484.6	677.8	335.7	427.0	585.1	300.3	19.8	31.8	7.7	-7.0	2.3	-16.5	-33.9	-28.2	-39.5
Zhejiang	159.15	225.33	112.11	500.8	709.1	352.8	400.4	561.8	282.5	15.1	27.0	2.6	-18.9	-10.4	-27.7	-39.1	-33.3	-44.3
**China**	4307.31	5857.23	3017.05	594.3	808.1	416.2	493.4	660.8	349.0	15.8	21.6	9.8	-2.5	2.3	-7.5	-29.2	-26.9	-31.6

Qinghai	24.09	32.78	16.82	789.7	1074.6	551.3	692.1	936.1	487.5	41.3	53.1	29.9	2.3	10.9	-5.9	-29.1	-24.8	-33.5
Shaanxi	145.28	197.52	102.87	765.8	1041.2	542.2	582.2	786.8	414.2	25.9	38.4	14.9	7.0	17.7	-2.3	-29.6	-23.8	-34.6
Shandong	384.98	522.15	273.91	757.7	1027.7	539.1	559.3	754.3	400.7	17.0	28.7	6.7	-2.3	7.5	-10.8	-32.5	-26.9	-37.9
Shanghai	100.60	136.46	71.88	733.0	994.3	523.8	539.8	725.9	387.2	64.3	79.2	51.6	-18.7	-11.3	-25.0	-31.9	-26.4	-37.0
Shanxi	163.04	220.42	113.44	868.8	1174.6	604.5	690.8	931.9	486.1	33.6	44.7	23.4	1.3	9.7	-6.4	-29.3	-24.6	-33.7
Sichuan	398.32	535.70	281.39	960.7	1292.1	678.7	698.5	934.6	495.5	-18.8	-11.1	-25.8	3.6	13.5	-5.2	-29.1	-24.5	-33.1
Tianjin	63.77	86.30	44.67	912.6	1235.0	639.2	679.3	911.9	481.6	53.6	66.0	42.2	-1.7	6.3	-8.9	-27.8	-23.0	-32.5
Tibet	11.37	15.50	7.96	678.6	924.6	475.0	688.2	932.0	488.4	25.7	34.7	17.8	-15.8	-9.8	-21.1	-29.0	-24.3	-33.4
Xinjiang	90.74	122.89	63.86	765.2	1036.3	538.5	682.0	912.0	485.6	63.4	77.0	51.4	2.4	10.9	-5.1	-28.9	-24.3	-33.1
Yunnan	156.95	216.09	111.11	656.1	903.3	464.4	551.6	750.8	393.1	22.5	34.9	11.2	-5.5	4.1	-14.2	-33.3	-27.4	-38.7
Zhejiang	214.46	296.69	152.56	715.2	989.4	508.7	534.1	738.0	375.8	23.7	37.6	10.3	-13.3	-3.6	-22.7	-38.2	-33.0	-43.5
**China**	6026.80	8179.08	4270.86	864.0	1172.6	612.3	662.8	884.0	471.4	24.7	31.7	17.4	2.5	8.3	-3.4	-29.2	-27.1	-31.3

Qinghai	42.70	58.32	29.61	671.3	916.7	465.5	595.0	801.2	418.8	41.2	51.6	31.7	2.1	9.7	-4.8	-29.2	-26.0	-32.7
Shaanxi	259.09	353.50	182.92	662.7	904.2	467.9	524.9	705.6	372.2	21.7	32.5	12.3	4.6	13.8	-3.5	-28.5	-23.6	-33.3
Shandong	682.51	925.24	481.41	663.5	899.5	468.0	517.9	695.9	369.4	16.7	27.5	7.8	-2.0	7.0	-9.5	-30.0	-24.9	-34.7
Shanghai	175.84	239.36	123.88	618.1	841.5	435.5	467.7	633.8	335.1	58.2	70.5	47.1	-22.7	-16.6	-28.1	-35.6	-31.1	-39.8
Shanxi	279.33	377.64	196.08	728.4	984.8	511.3	593.6	799.8	416.9	28.5	36.4	20.1	-0.5	5.6	-7.0	-29.3	-26.0	-32.5
Sichuan	662.41	897.89	469.21	798.1	1081.8	565.3	602.1	807.9	425.2	-23.8	-18.3	-29.4	0.9	8.3	-6.5	-29.2	-25.6	-32.4
Tianjin	116.14	158.59	81.05	744.7	1016.8	519.7	580.9	781.6	413.9	61.1	71.1	52.3	-5.7	0.1	-10.9	-28.5	-25.1	-31.8
Tibet	20.15	27.37	14.01	578.8	786.4	402.5	593.9	801.0	419.5	32.4	39.8	25.7	-14.3	-9.4	-18.6	-29.6	-26.2	-32.9
Xinjiang	160.79	218.69	113.06	656.1	892.3	461.3	584.8	788.9	415.3	60.5	71.2	50.6	0.9	7.6	-5.4	-28.7	-25.4	-31.9
Yunnan	279.41	383.42	198.69	568.0	779.4	403.9	491.1	668.5	350.7	21.3	31.3	11.6	-6.1	1.6	-13.7	-33.3	-28.6	-37.9
Zhejiang	373.60	521.49	266.27	604.9	844.3	431.1	467.2	645.5	331.0	19.9	30.9	8.4	-15.7	-8.0	-23.8	-38.3	-34.2	-42.8
**China**	10334.10	14004.81	7329.88	726.6	984.6	515.3	579.1	778.1	411.6	20.8	26.9	14.3	0.6	5.6	-4.9	-29.0	-27.0	-30.8

Footnote: LBP: low back pain; YLDs: years lived with disability;

* Changes are the total mean values at 2019 compared with 1990

For each indicators, the 1st column was mean value and the 2nd and 3rd were upper uncertainty interval (UUI) and lower uncertainty interval (LUI) respectively.

Table S5 The prevalence of LBP and the proportion of each age-group (5-14y, 15-49y, 50-69y, and ≥70y) in all provinces/regions in China

province-level administrative units	Prevalence in 1990 (per 100,000)				Prevalence in 2019 (per 100,000)				Proportion 1990 (%)				Proportion 2019 (%)			
	5-14	15-49	50-69	≥70	5-14	15-49	50-69	≥70	5-14 (%)	15-49 (%)	50-69 (%)	≥70 (%)	5-14 (%)	15-49 (%)	50-69 (%)	≥70 (%)
Anhui	1606.7	6236.9	15627.6	23750.7	1446.6	5085.2	10533.5	15817.7	3.4	13.2	33.1	50.3	4.4	15.5	32.0	48.1
Beijing	1289.8	6057.1	14552.4	22361.1	1377.9	4775.3	10382.4	16062.0	2.9	13.7	32.9	50.5	4.2	14.6	31.9	49.3
Chongqing	1527.4	6425.8	15546.8	23425.4	1513.6	5089.8	10823.7	15857.4	3.3	13.7	33.1	49.9	4.5	15.3	32.5	47.6
Fujian	1511.9	6045.5	15336.5	23436.3	1395.1	4939.3	10324.9	15859.5	3.3	13.0	33.1	50.6	4.3	15.2	31.8	48.8
Gansu	1520.5	6228.0	15199.6	23177.6	1490.6	5043.6	10532.0	15762.8	3.3	13.5	33.0	50.2	4.5	15.4	32.1	48.0
Guangdong	1315.5	5725.8	14479.8	22182.2	1619.6	5352.9	11487.9	17070.9	3.0	13.1	33.1	50.8	4.6	15.1	32.3	48.0
Guangxi	1537.4	6248.9	15592.5	23862.6	1494.2	4944.1	10544.5	16176.7	3.3	13.2	33.0	50.5	4.5	14.9	31.8	48.8
Guizhou	1641.1	6287.1	15526.0	23861.3	1576.0	5087.6	10708.8	16022.3	3.5	13.3	32.8	50.4	4.7	15.2	32.1	48.0
Hainan	1469.5	6122.1	15482.7	24000.9	1448.8	4871.2	10290.2	16037.1	3.1	13.0	32.9	51.0	4.4	14.9	31.5	49.1
Hebei	1448.3	6480.0	15708.6	23653.6	1476.0	4906.3	10768.2	15839.1	3.1	13.7	33.2	50.0	4.5	14.9	32.6	48.0
Heilongjiang	1590.3	6242.5	15266.1	23144.3	1594.8	5276.2	10819.0	16021.2	3.4	13.5	33.0	50.0	4.7	15.7	32.1	47.5
Henan	1584.2	6324.4	15924.1	23951.9	1484.7	4911.9	10834.6	16115.1	3.3	13.2	33.3	50.1	4.5	14.7	32.5	48.3
Hong Kong	1462.3	6374.1	14846.4	21139.4	1789.2	5951.2	12515.8	19216.2	3.3	14.5	33.9	48.2	4.5	15.1	31.7	48.7
Hubei	1163.5	5335.8	13725.7	20761.7	1184.3	4466.9	9222.5	13743.2	2.8	13.0	33.5	50.7	4.1	15.6	32.2	48.0
Hunan	1528.3	6272.3	15513.9	23641.4	1506.8	5028.3	10633.9	15978.1	3.3	13.4	33.0	50.3	4.5	15.2	32.1	48.2
Inner Mongolia	1520.0	6169.9	15109.1	22888.9	1516.1	5165.3	10529.7	15914.2	3.3	13.5	33.1	50.1	4.6	15.6	31.8	48.0
Jiangsu	1549.8	6382.1	15554.5	23473.3	1435.6	4916.3	10529.1	15919.6	3.3	13.6	33.1	50.0	4.4	15.0	32.1	48.5
Jiangxi	1667.4	6096.1	15532.2	23594.4	1474.4	4934.7	10528.9	15810.2	3.6	13.0	33.1	50.3	4.5	15.1	32.2	48.3
Jilin	1263.3	5366.5	13562.0	20489.2	1389.8	4496.6	9991.4	15063.8	3.1	13.2	33.3	50.4	4.5	14.5	32.3	48.7
Liaoning	1576.7	6384.9	15492.6	23082.2	1529.4	5113.0	10799.2	16019.1	3.4	13.7	33.3	49.6	4.6	15.3	32.3	47.9
Macao	1289.7	6098.8	15473.4	23344.0	1343.6	4746.6	10598.7	16116.1	2.8	13.2	33.5	50.5	4.1	14.5	32.3	49.1
Ningxia	1572.9	6084.8	15060.8	22802.5	1504.8	4939.0	10335.4	15598.0	3.5	13.4	33.1	50.1	4.6	15.3	31.9	48.2
Qinghai	1629.3	6012.8	14873.6	23197.8	1517.9	5002.1	10157.6	15596.3	3.6	13.2	32.5	50.7	4.7	15.5	31.5	48.3
Shaanxi	1150.2	5400.6	13579.0	20451.0	1248.3	4309.4	9291.4	13865.7	2.8	13.3	33.5	50.4	4.3	15.0	32.4	48.3

Shandong	1189.2	5459.2	13876.3	20996.4	1262.8	4284.3	9222.8	13690.2	2.9	13.1	33.4	50.6	4.4	15.1	32.4	48.1
Shanghai	1023.3	5757.0	14095.3	20908.0	1122.6	3978.1	8217.6	12678.3	2.4	13.8	33.7	50.0	4.3	15.3	31.6	48.8
Shanxi	1491.3	6208.9	15401.4	23136.6	1498.1	4917.6	10433.4	15707.5	3.2	13.4	33.3	50.0	4.6	15.1	32.0	48.2
Sichuan	1639.2	6358.7	15587.1	23606.1	1523.5	5146.5	10811.3	15717.4	3.5	13.5	33.0	50.0	4.6	15.5	32.6	47.3
Tianjin	1379.4	6284.4	15043.3	22441.5	1404.6	4717.0	10315.5	15571.4	3.1	13.9	33.3	49.7	4.4	14.7	32.2	48.6
Tibet	1455.6	6173.9	15646.7	23412.4	1476.3	4742.8	10225.2	15804.2	3.1	13.2	33.5	50.1	4.6	14.7	31.7	49.0
Xinjiang	1485.8	6045.1	14649.6	22353.7	1457.8	4947.2	10070.6	15266.7	3.3	13.6	32.9	50.2	4.6	15.6	31.7	48.1
Yunnan	1228.6	5237.5	13757.8	20985.8	1242.4	3877.4	9050.1	13981.0	3.0	12.7	33.4	50.9	4.4	13.8	32.1	49.7
Zhejiang	1173.3	5665.7	14312.4	21826.0	1086.7	4096.9	7809.6	12941.7	2.7	13.2	33.3	50.8	4.2	15.8	30.1	49.9
**China**	1453.5	6044.9	15036.9	22766.5	1435.4	4834.0	10251.6	15399.9	3.2	13.3	33.2	50.3	4.5	15.1	32.1	48.2

Footnote: LBP: low back pain

Table S6 The YLDs of LBP and the proportion of each age-group (5-14y, 15-49y, 50-69y, and ≥70y) in all provinces/regions in China

province-level administrative units	Prevalence in 1990 (per 100,000)				Prevalence in 2019 (per 100,000)				Proportion 1990 (%)				Proportion 2019 (%)			
	5-14	15-49	50-69	≥70	5-14	15-49	50-69	≥70	5-14 (%)	15-49 (%)	50-69 (%)	≥70 (%)	5-14 (%)	15-49 (%)	50-69 (%)	≥70 (%)
Anhui	175.8	720.3	1771.6	2499.5	158.8	593.9	1204.0	1658.9	4.4	16.4	33.3	45.9	4.4	16.4	33.3	45.9
Beijing	141.4	703.1	1649.8	2340.3	151.5	557.6	1179.0	1670.0	4.3	15.7	33.1	46.9	4.3	15.7	33.1	46.9
Chongqing	167.6	743.1	1759.2	2473.4	165.9	593.0	1226.8	1651.5	4.6	16.3	33.7	45.4	4.6	16.3	33.7	45.4
Fujian	165.6	699.8	1742.8	2471.7	153.3	577.8	1181.9	1656.6	4.3	16.2	33.1	46.4	4.3	16.2	33.1	46.4
Gansu	166.0	717.6	1725.1	2451.5	163.7	587.3	1198.2	1652.3	4.5	16.3	33.3	45.9	4.5	16.3	33.3	45.9
Guangdong	144.2	663.3	1641.8	2327.9	178.0	623.4	1308.6	1771.3	4.6	16.1	33.7	45.6	4.6	16.1	33.7	45.6
Guangxi	168.6	722.6	1770.1	2508.2	164.5	576.3	1203.0	1689.5	4.5	15.9	33.1	46.5	4.5	15.9	33.1	46.5
Guizhou	179.1	723.3	1754.4	2511.0	173.1	590.5	1215.1	1673.0	4.7	16.2	33.3	45.8	4.7	16.2	33.3	45.8
Hainan	161.3	707.5	1751.9	2510.1	158.9	567.5	1172.6	1663.1	4.5	15.9	32.9	46.7	4.5	15.9	32.9	46.7
Hebei	159.4	752.7	1788.6	2495.0	162.1	572.6	1225.7	1655.9	4.5	15.8	33.9	45.8	4.5	15.8	33.9	45.8
Heilongjiang	174.4	721.7	1729.5	2429.3	175.0	615.3	1229.2	1675.5	4.7	16.7	33.3	45.3	4.7	16.7	33.3	45.3
Henan	173.6	732.6	1811.6	2522.9	163.3	572.1	1236.2	1684.2	4.5	15.6	33.8	46.1	4.5	15.6	33.8	46.1
Hong Kong	160.4	742.1	1692.1	2231.7	196.6	693.8	1427.4	1988.2	4.6	16.1	33.1	46.2	4.6	16.1	33.1	46.2
Hubei	127.7	617.5	1559.0	2191.4	130.1	522.2	1053.0	1445.9	4.1	16.6	33.4	45.9	4.1	16.6	33.4	45.9
Hunan	167.5	724.4	1755.6	2489.9	165.2	586.2	1208.8	1665.9	4.6	16.2	33.3	45.9	4.6	16.2	33.3	45.9
Inner Mongolia	166.5	714.7	1718.6	2418.6	166.7	602.5	1199.4	1663.6	4.6	16.6	33.0	45.8	4.6	16.6	33.0	45.8
Jiangsu	170.1	739.0	1761.2	2465.4	158.1	574.2	1199.2	1650.7	4.4	16.0	33.5	46.1	4.4	16.0	33.5	46.1
Jiangxi	182.0	704.0	1760.1	2489.3	161.6	575.5	1202.3	1658.2	4.5	16.0	33.4	46.1	4.5	16.0	33.4	46.1
Jilin	138.5	622.2	1543.3	2165.5	153.0	526.6	1140.3	1585.9	4.5	15.5	33.5	46.6	4.5	15.5	33.5	46.6
Liaoning	172.7	742.1	1760.6	2432.5	167.9	597.4	1227.3	1671.7	4.6	16.3	33.5	45.6	4.6	16.3	33.5	45.6
Macao	141.6	710.1	1761.8	2448.2	147.8	554.7	1211.0	1673.7	4.1	15.5	33.8	46.7	4.1	15.5	33.8	46.7
Ningxia	172.3	704.5	1719.2	2427.9	165.3	576.0	1180.0	1639.7	4.6	16.2	33.1	46.0	4.6	16.2	33.1	46.0
Qinghai	178.5	695.2	1697.3	2472.5	166.8	583.2	1162.0	1649.0	4.7	16.4	32.6	46.3	4.7	16.4	32.6	46.3
Shaanxi	126.2	626.1	1546.6	2182.0	137.1	504.3	1063.4	1466.2	4.3	15.9	33.5	46.2	4.3	15.9	33.5	46.2

Shandong	130.7	633.7	1582.6	2225.0	138.4	500.5	1057.1	1439.4	4.4	16.0	33.7	45.9	4.4	16.0	33.7	45.9
Shanghai	112.1	667.2	1590.3	2187.2	123.5	464.1	933.5	1314.0	4.4	16.4	32.9	46.3	4.4	16.4	32.9	46.3
Shanxi	163.1	719.3	1747.1	2441.9	164.6	574.6	1190.0	1649.9	4.6	16.1	33.2	46.1	4.6	16.1	33.2	46.1
Sichuan	179.3	733.8	1764.4	2498.9	167.3	597.7	1223.8	1643.9	4.6	16.5	33.7	45.3	4.6	16.5	33.7	45.3
Tianjin	151.2	731.2	1711.0	2370.5	154.4	552.9	1176.0	1632.3	4.4	15.7	33.5	46.4	4.4	15.7	33.5	46.4
Tibet	158.5	712.0	1772.2	2462.6	161.9	552.3	1167.7	1657.5	4.6	15.6	33.0	46.8	4.6	15.6	33.0	46.8
Xinjiang	163.1	698.0	1664.1	2367.0	160.1	576.5	1148.0	1602.7	4.6	16.5	32.9	46.0	4.6	16.5	32.9	46.0
Yunnan	134.4	603.7	1555.8	2208.8	136.2	450.3	1027.0	1457.5	4.4	14.7	33.4	47.5	4.4	14.7	33.4	47.5
Zhejiang	128.5	655.2	1618.8	2284.8	119.3	478.2	889.3	1338.9	4.2	16.9	31.5	47.4	4.2	16.9	31.5	47.4
**China**	159.3	699.5	1706.2	2399.8	157.7	563.8	1167.9	1608.7	4.5	16.1	33.4	46.0	4.5	16.1	33.4	46.0

Footnote: LBP: low back pain; YLDs: years lived with disability

Table S7 SDI of all provinces/regions in China from 1990 to 2019

Region	Province	1990	1991	1992	1993	1994	1995	1996	1997	1998	1999	2000	2001	2002	2003	2004	2005	2006	2007	2008	2009	2010	2011	2012	2013	2014	2015	2016	2017	2018	2019
Central	Anhui	0.363	0.373	0.384	0.393	0.401	0.409	0.421	0.433	0.442	0.450	0.459	0.466	0.469	0.474	0.482	0.494	0.506	0.517	0.528	0.540	0.554	0.565	0.573	0.579	0.588	0.592	0.594	0.605	0.618	0.626
Eastern	Beijing	0.591	0.601	0.610	0.620	0.627	0.636	0.647	0.658	0.667	0.677	0.687	0.695	0.703	0.711	0.720	0.730	0.739	0.748	0.756	0.764	0.772	0.779	0.784	0.789	0.795	0.799	0.802	0.808	0.814	0.819
Western	Chongqing	0.419	0.428	0.438	0.447	0.454	0.460	0.471	0.481	0.488	0.494	0.500	0.505	0.507	0.510	0.517	0.526	0.537	0.548	0.559	0.571	0.584	0.596	0.605	0.613	0.623	0.630	0.635	0.647	0.659	0.668
Eastern	Fujian	0.431	0.442	0.452	0.463	0.470	0.479	0.491	0.504	0.515	0.524	0.534	0.541	0.546	0.551	0.559	0.569	0.580	0.590	0.599	0.609	0.620	0.629	0.635	0.640	0.647	0.651	0.652	0.662	0.672	0.680
Western	Gansu	0.334	0.344	0.354	0.364	0.371	0.379	0.392	0.404	0.414	0.423	0.433	0.441	0.445	0.452	0.461	0.473	0.485	0.497	0.508	0.520	0.533	0.543	0.551	0.558	0.566	0.571	0.574	0.584	0.594	0.602
Eastern	Guangdong	0.484	0.495	0.506	0.517	0.526	0.535	0.548	0.560	0.570	0.579	0.589	0.597	0.602	0.609	0.618	0.628	0.639	0.649	0.658	0.668	0.678	0.686	0.692	0.697	0.703	0.707	0.710	0.719	0.727	0.734
Western	Guangxi	0.396	0.406	0.416	0.426	0.433	0.441	0.452	0.464	0.472	0.480	0.487	0.493	0.496	0.501	0.508	0.519	0.531	0.541	0.552	0.564	0.576	0.586	0.593	0.599	0.607	0.611	0.613	0.624	0.635	0.643
Western	Guizhou	0.298	0.308	0.318	0.327	0.334	0.341	0.352	0.363	0.372	0.380	0.388	0.394	0.397	0.401	0.410	0.422	0.436	0.449	0.460	0.474	0.489	0.501	0.510	0.518	0.529	0.535	0.538	0.553	0.569	0.579
Eastern	Hainan	0.420	0.431	0.443	0.454	0.460	0.467	0.479	0.490	0.498	0.505	0.513	0.518	0.520	0.524	0.531	0.541	0.552	0.562	0.572	0.583	0.595	0.604	0.611	0.618	0.626	0.631	0.634	0.645	0.656	0.664
Eastern	Hebei	0.422	0.432	0.443	0.452	0.460	0.468	0.480	0.493	0.503	0.512	0.522	0.529	0.534	0.540	0.549	0.560	0.572	0.583	0.593	0.604	0.616	0.625	0.631	0.636	0.642	0.645	0.646	0.655	0.664	0.671
Central	Heilongjiang	0.460	0.470	0.481	0.490	0.497	0.505	0.517	0.528	0.537	0.545	0.553	0.559	0.563	0.568	0.575	0.585	0.595	0.604	0.613	0.622	0.631	0.640	0.646	0.651	0.658	0.661	0.664	0.671	0.678	0.683
Central	Henan	0.393	0.403	0.414	0.424	0.432	0.441	0.454	0.467	0.477	0.487	0.497	0.505	0.511	0.517	0.527	0.540	0.553	0.565	0.577	0.589	0.602	0.612	0.618	0.624	0.632	0.636	0.638	0.648	0.659	0.667
Eastern	Hong Kong	0.660	0.665	0.671	0.677	0.685	0.693	0.700	0.707	0.712	0.718	0.723	0.727	0.732	0.736	0.741	0.745	0.750	0.755	0.760	0.764	0.770	0.778	0.786	0.792	0.798	0.802	0.807	0.812	0.817	0.821
Central	Hubei	0.409	0.419	0.430	0.440	0.448	0.456	0.469	0.481	0.490	0.499	0.508	0.515	0.520	0.526	0.534	0.545	0.558	0.569	0.580	0.592	0.606	0.617	0.625	0.632	0.640	0.645	0.648	0.658	0.669	0.677
Central	Hunan	0.402	0.411	0.421	0.431	0.438	0.446	0.458	0.470	0.479	0.488	0.497	0.504	0.508	0.514	0.522	0.533	0.545	0.556	0.567	0.579	0.592	0.602	0.610	0.616	0.625	0.629	0.632	0.643	0.654	0.662
Central	Inner Mongolia	0.417	0.427	0.438	0.448	0.456	0.465	0.477	0.489	0.499	0.509	0.519	0.527	0.534	0.542	0.553	0.568	0.583	0.597	0.611	0.625	0.639	0.650	0.658	0.665	0.672	0.676	0.677	0.685	0.693	0.699
Eastern	Jiangsu	0.456	0.466	0.476	0.486	0.493	0.500	0.512	0.524	0.533	0.542	0.551	0.559	0.563	0.570	0.579	0.592	0.605	0.617	0.629	0.641	0.654	0.664	0.671	0.678	0.686	0.690	0.693	0.703	0.713	0.720
Central	Jiangxi	0.376	0.386	0.396	0.406	0.413	0.420	0.432	0.444	0.454	0.462	0.471	0.478	0.481	0.486	0.495	0.507	0.521	0.533	0.544	0.557	0.570	0.581	0.588	0.594	0.603	0.606	0.608	0.620	0.632	0.641
Central	Jilin	0.448	0.459	0.470	0.480	0.488	0.496	0.508	0.519	0.528	0.537	0.546	0.553	0.559	0.565	0.574	0.585	0.597	0.608	0.619	0.630	0.642	0.653	0.661	0.668	0.676	0.680	0.684	0.692	0.700	0.706
Eastern	Liaoning	0.490	0.500	0.510	0.520	0.527	0.534	0.544	0.555	0.563	0.571	0.579	0.585	0.589	0.595	0.602	0.612	0.623	0.632	0.642	0.652	0.663	0.672	0.679	0.684	0.691	0.694	0.695	0.701	0.708	0.713
Eastern	Macao	0.672	0.678	0.685	0.693	0.700	0.707	0.714	0.720	0.725	0.730	0.734	0.738	0.743	0.748	0.755	0.762	0.770	0.779	0.787	0.795	0.801	0.806	0.812	0.817	0.822	0.827	0.831	0.835	0.839	0.843
Western	Ningxia	0.373	0.384	0.395	0.405	0.412	0.419	0.432	0.444	0.454	0.463	0.473	0.480	0.483	0.489	0.498	0.511	0.525	0.538	0.550	0.564	0.580	0.591	0.599	0.607	0.616	0.620	0.623	0.635	0.648	0.656
Western	Qinghai	0.363	0.373	0.383	0.393	0.399	0.406	0.417	0.428	0.436	0.444	0.452	0.458	0.462	0.467	0.475	0.486	0.499	0.510	0.521	0.533	0.546	0.556	0.564	0.570	0.579	0.584	0.586	0.597	0.608	0.616
Western	Shaanxi	0.394	0.405	0.415	0.426	0.434	0.442	0.454	0.466	0.476	0.486	0.496	0.504	0.511	0.519	0.530	0.544	0.558	0.572	0.585	0.599	0.614	0.626	0.636	0.645	0.654	0.660	0.665	0.675	0.684	0.692
Eastern	Shandong	0.440	0.450	0.460	0.470	0.478	0.486	0.498	0.510	0.519	0.528	0.538	0.545	0.551	0.558	0.567	0.579	0.591	0.603	0.613	0.624	0.635	0.644	0.650	0.655	0.662	0.665	0.667	0.676	0.685	0.692

Eastern	Shanghai	0.582	0.592	0.602	0.611	0.618	0.626	0.637	0.648	0.658	0.666	0.676	0.683	0.689	0.695	0.703	0.713	0.722	0.731	0.738	0.746	0.754	0.760	0.765	0.769	0.774	0.777	0.779	0.785	0.792	0.797
Central	Shanxi	0.412	0.423	0.433	0.443	0.451	0.459	0.471	0.484	0.494	0.503	0.513	0.520	0.526	0.533	0.543	0.556	0.570	0.582	0.594	0.606	0.618	0.628	0.636	0.642	0.649	0.653	0.655	0.664	0.673	0.680
Western	Sichuan	0.355	0.365	0.375	0.385	0.393	0.401	0.414	0.427	0.437	0.447	0.457	0.465	0.471	0.477	0.487	0.499	0.512	0.524	0.536	0.548	0.561	0.572	0.579	0.586	0.595	0.600	0.603	0.614	0.625	0.633
Eastern	Tianjin	0.542	0.551	0.561	0.571	0.579	0.587	0.599	0.611	0.620	0.630	0.640	0.648	0.655	0.662	0.672	0.684	0.696	0.707	0.717	0.729	0.740	0.750	0.758	0.766	0.773	0.779	0.783	0.790	0.798	0.803
Western	Tibet	0.235	0.242	0.250	0.258	0.264	0.271	0.281	0.291	0.300	0.309	0.319	0.327	0.333	0.340	0.349	0.359	0.369	0.379	0.387	0.396	0.406	0.414	0.420	0.426	0.433	0.437	0.440	0.450	0.460	0.468
Western	Xinjiang	0.431	0.441	0.452	0.462	0.469	0.476	0.487	0.499	0.507	0.514	0.522	0.528	0.531	0.534	0.541	0.551	0.562	0.572	0.580	0.591	0.602	0.610	0.616	0.622	0.629	0.633	0.635	0.645	0.656	0.664
Western	Yunnan	0.344	0.354	0.365	0.375	0.382	0.389	0.401	0.413	0.422	0.430	0.438	0.444	0.446	0.450	0.457	0.467	0.478	0.487	0.497	0.508	0.520	0.530	0.537	0.544	0.554	0.559	0.562	0.574	0.586	0.595
Eastern	Zhejiang	0.451	0.461	0.472	0.481	0.488	0.496	0.508	0.520	0.530	0.539	0.549	0.557	0.563	0.570	0.579	0.591	0.604	0.615	0.625	0.636	0.647	0.656	0.662	0.667	0.674	0.678	0.681	0.689	0.698	0.705
	**China**	0.429	0.439	0.450	0.460	0.467	0.475	0.487	0.499	0.508	0.517	0.527	0.534	0.539	0.545	0.554	0.566	0.578	0.589	0.599	0.610	0.622	0.632	0.638	0.644	0.652	0.655	0.658	0.667	0.677	0.685

Footnote: SDI: socio-demographic index

Table S8 The number and point prevalence of LBP by 21 regions all over the world (2019 and the 30-year change)

Regions	men																		women																		Both																	
	2019									30-year change*									2019									30-year change*									2019									30-year change*								
	Number (×100,000)			All-age prevalence (per 100)			All-age prevalence (per 100)			Number (%)			All-age prevalence (%)			Age-standardized (%)			Number (×100,000)			All-age prevalence (per 100)			Age-standardized (per 100)			Number (%)			All-age prevalence (%)			Age-standardized (%)			Number (×100,000)			All-age prevalence (per 100)			Age-standardized (per 100)			Number (%)			All-age prevalence (%)			Age-standardized (%)		
Andean Latin America	16.3	18.3	14.4	5.1	5.8	4.5	5.4	6.1	4.8	109.4	118.8	100.1	25.0	30.6	19.4	-1.3	2.3	-4.9	20.4	22.9	18.2	6.4	7.2	5.7	6.5	7.3	5.8	112.9	123.9	102.2	28.6	35.2	22.1	0.1	4.2	-3.8	36.7	41.1	32.6	5.8	6.5	5.1	6.0	6.7	5.3	111.3	119.9	103.5	26.9	32.0	22.1	-0.5	2.2	-3.1
Australasia	14.2	16.3	12.3	9.9	11.4	8.6	8.1	9.3	7.0	38.4	48.3	28.4	-2.6	4.4	-9.7	-14.6	-9.2	-20.2	17.5	20.0	15.3	11.9	13.6	10.4	9.5	10.8	8.2	51.8	62.0	42.1	5.0	12.1	-1.7	-7.1	-1.2	-13.1	31.7	36.4	27.7	10.9	12.5	9.5	8.8	10.1	7.7	45.5	53.9	36.6	1.5	7.4	-4.7	-10.7	-5.9	-15.7
Caribbean	12.8	14.4	11.3	5.5	6.2	4.9	5.3	5.9	4.7	56.2	61.6	51.0	16.9	20.9	13.0	-2.5	0.3	-4.9	19.6	21.9	17.6	8.2	9.2	7.4	7.6	8.5	6.8	61.7	68.0	55.7	20.9	25.6	16.4	-1.2	2.2	-4.3	32.4	36.4	29.0	6.9	7.7	6.1	6.5	7.2	5.8	59.5	64.8	54.6	19.3	23.3	15.6	-1.6	0.7	-3.8
Central Asia	28.5	32.4	24.9	6.1	7.0	5.4	6.8	7.7	6.1	59.4	64.8	54.0	16.6	20.5	12.6	-1.6	0.7	-3.8	36.0	40.8	31.5	7.6	8.7	6.7	7.8	8.8	6.9	52.1	58.1	47.2	14.0	18.5	10.4	-0.9	1.6	-3.3	64.4	73.1	56.5	6.9	7.8	6.0	7.3	8.3	6.5	55.3	60.3	50.9	15.0	18.7	11.7	-1.4	0.4	-2.9
Central Europe	71.7	81.8	63.5	12.9	14.7	11.4	9.8	11.1	8.7	10.9	14.4	7.7	20.0	23.8	16.6	-2.3	-0.8	-3.8	80.9	91.9	72.0	13.8	15.7	12.3	10.2	11.6	9.0	7.7	11.2	4.4	15.4	19.1	11.9	-3.3	-1.7	-4.9	152.6	174.0	135.8	13.4	15.2	11.9	10.0	11.3	8.8	9.2	12.4	6.3	17.6	21.0	14.5	-2.8	-1.6	-4.0
Central Latin America	58.5	66.2	51.2	4.8	5.4	4.2	4.8	5.5	4.3	110.0	118.0	102.2	39.3	44.6	34.1	5.7	7.8	3.7	114.7	129.7	100.7	9.0	10.1	7.9	8.6	9.8	7.6	95.9	104.2	87.5	27.3	32.7	21.8	-3.4	-1.6	-5.1	173.2	195.8	152.1	6.9	7.8	6.1	6.8	7.7	6.0	100.4	108.2	92.4	31.6	36.7	26.3	0.0	1.4	-1.4
Central Sub-Saharan Africa	27.7	31.6	24.0	4.2	4.8	3.7	6.5	7.4	5.8	151.3	162.1	140.2	5.7	10.3	1.0	0.3	4.1	-3.6	29.7	33.8	25.7	4.5	5.1	3.9	6.8	7.7	6.0	140.6	151.0	131.0	1.9	6.3	-2.2	-2.5	1.7	-6.3	57.4	65.4	49.7	4.4	5.0	3.8	6.7	7.5	5.9	145.6	153.4	138.2	3.7	6.9	0.5	-1.1	1.6	-3.9
East Asia	395.7	451.2	348.0	5.3	6.0	4.6	4.4	5.0	3.9	17.1	22.5	11.5	-1.5	3.0	-6.1	-28.7	-26.5	-30.9	566.4	648.7	500.4	7.8	9.0	6.9	6.0	6.8	5.3	26.0	32.5	18.9	3.7	9.0	-2.2	-28.8	-27.0	-30.7	962.1	1096.8	848.7	6.5	7.5	5.8	5.2	5.9	4.6	22.2	28.0	15.9	1.7	6.5	-3.6	-28.5	-26.7	-30.2
Eastern Europe	88.0	100.2	77.9	9.0	10.3	8.0	7.4	8.4	6.6	4.1	7.2	1.2	12.9	16.3	9.7	-5.0	-3.4	-6.4	167.9	189.7	149.4	14.9	16.9	13.3	10.7	12.0	9.5	7.1	9.4	4.7	15.0	17.4	12.5	-0.2	1.2	-1.6	256.0	289.1	228.0	12.2	13.8	10.9	9.2	10.3	8.2	6.1	8.4	3.7	14.4	16.9	11.9	-2.3	-1.2	-3.3
Eastern Sub-Saharan Africa	91.1	103.8	79.2	4.5	5.1	3.9	7.0	7.9	6.2	133.5	137.9	129.1	7.7	9.7	5.7	0.9	2.3	-0.5	74.5	84.3	65.0	3.6	4.1	3.1	5.8	6.6	5.1	125.5	129.1	121.5	4.2	5.9	2.4	-3.1	-1.7	-4.5	165.6	187.9	144.1	4.0	4.6	3.5	6.4	7.2	5.6	129.8	133.0	126.4	6.1	7.6	4.6	-1.0	0.0	-1.9
Global	2368.3	2662.4	2096.1	6.1	6.9	5.4	5.9	6.7	5.3	46.8	49.9	43.3	1.9	4.1	-0.5	-16.2	-15.4	-16.9	3316.1	3736.8	2951.1	8.6	9.7	7.7	7.9	9.0	7.1	47.6	51.3	43.8	1.6	4.2	-0.9	-16.6	-15.7	-17.5	5684.4	6406.0	5050.0	7.3	8.3	6.5	7.0	7.9	6.2	47.3	50.6	43.6	1.8	4.2	-0.7	-16.4	-15.6	-17.1
High-income Asia Pacific	95.7	109.2	83.4	10.4	11.8	9.0	7.4	8.4	6.4	24.8	31.8	18.8	15.9	22.5	10.4	-9.5	-7.7	-11.0	161.2	182.5	142.0	17.0	19.2	15.0	11.6	13.2	10.2	18.1	23.6	12.9	9.1	14.2	4.3	-12.9	-11.3	-14.4	256.9	291.5	225.5	13.7	15.6	12.0	9.5	10.8	8.3	20.5	26.4	15.3	11.6	17.1	6.8	-12.4	-11.0	-13.5
High-income North America	246.8	269.4	227.2	13.8	15.0	12.7	11.2	12.1	10.3	32.2	40.0	25.0	1.2	7.2	-4.3	-11.4	-6.7	-15.6	316.8	344.4	292.8	17.1	18.6	15.8	13.4	14.6	12.3	29.2	35.9	22.2	0.1	5.4	-5.2	-10.1	-5.4	-14.6	563.6	612.7	520.6	15.5	16.8	14.3	12.3	13.4	11.3	30.5	37.4	23.3	0.5	5.9	-5.0	-10.9	-6.3	-15.3
North Africa and Middle East	229.6	259.6	200.6	7.3	8.2	6.3	7.8	8.8	6.9	113.6	121.9	104.5	19.4	24.0	14.3	-8.5	-6.1	-11.0	202.8	228.4	178.0	6.9	7.8	6.1	7.5	8.5	6.7	117.3	123.7	110.0	25.0	28.7	20.8	-2.7	-1.1	-4.3	432.4	488.2	377.7	7.1	8.0	6.2	7.7	8.6	6.8	115.4	121.8	107.9	22.1	25.7	17.8	-5.8	-4.3	-7.4
Oceania	4.0	4.6	3.5	5.9	6.7	5.2	7.6	8.6	6.8	124.8	134.4	116.1	9.9	14.7	5.7	0.1	3.8	-3.2	5.1	5.8	4.4	7.9	9.0	6.9	10.1	11.4	9.0	127.6	137.1	117.9	10.5	15.1	5.8	0.0	3.8	-3.6	9.1	10.3	7.9	6.9	7.8	6.0	8.8	10.0	7.8	126.3	134.1	119.5	10.3	14.1	7.0	0.1	2.9	-2.3
South Asia	317.4	360.8	277.5	3.4	3.9	3.0	3.9	4.4	3.4	50.2	54.6	45.1	-7.0	-4.3	-10.1	-24.0	-22.5	-25.8	577.4	657.3	505.5	6.5	7.4	5.7	7.1	8.0	6.2	65.3	71.9	59.3	-1.4	2.5	-5.0	-22.7	-20.7	-24.6	894.7	1016.9	784.6	5.0	5.6	4.3	5.5	6.2	4.8	59.6	64.9	54.1	-3.0	0.3	-6.3	-22.2	-20.7	-23.8
Southeast Asia	224.1	253.9	197.3	6.7	7.5	5.9	6.8	7.6	6.0	86.5	93.4	80.1	28.5	33.2	24.0	-3.0	-1.5	-4.4	321.4	364.5	283.3	9.5	10.8	8.4	9.2	10.3	8.1	87.9	94.6	81.3	30.9	35.6	26.3	-1.0	0.8	-2.7	545.4	616.0	481.0	8.1	9.1	7.1	8.0	9.0	7.1	87.3	94.1	81.2	29.8	34.5	25.6	-2.0	-0.6	-3.1

Southern Latin America	21.4	24.7	18.6	6.6	7.6	5.7	6.0	6.9	5.2	55.2	61.9	48.3	15.6	20.6	10.5	-0.8	3.3	-5.1	31.0	35.4	27.0	9.1	10.4	7.9	7.9	9.1	6.8
Southern Sub-Saharan Africa	18.6	21.1	16.3	4.8	5.5	4.2	5.7	6.5	5.1	75.4	80.1	70.7	16.3	19.4	13.2	-5.4	-3.5	-7.3	21.2	24.0	18.7	5.3	6.0	4.6	5.8	6.6	5.1
Tropical Latin America	81.6	92.6	71.3	7.5	8.5	6.5	7.0	7.9	6.2	86.2	93.3	79.0	28.8	33.7	23.8	-0.6	1.1	-2.2	105.1	119.2	92.4	9.2	10.4	8.1	8.3	9.4	7.3
Western Europe	221.0	252.6	193.7	10.3	11.8	9.0	7.9	9.0	7.0	18.9	23.7	14.3	3.8	7.9	-0.2	-8.3	-6.1	-10.4	333.5	378.1	295.5	15.0	17.1	13.3	10.9	12.4	9.6
Western Sub-Saharan Africa	103.8	117.8	90.9	4.7	5.3	4.1	7.0	7.9	6.2	127.1	131.3	123.0	-2.6	-0.8	-4.3	-1.1	-0.1	-2.1	113.0	128.4	99.0	4.8	5.5	4.2	7.2	8.1	6.4

Southern Latin America	55.0	61.7	48.4	14.6	19.6	9.8	-1.4	2.8	-5.4	52.4	60.0	45.7	7.9	9.0	6.8	7.0	8.0	6.1	55.0	60.4	50.1	15.1	19.0	11.4	-1.2	1.9	-4.1
Southern Sub-Saharan Africa	79.3	84.8	74.2	20.6	24.3	17.3	-4.8	-2.8	-6.6	39.7	45.0	34.9	5.1	5.7	4.4	5.8	6.5	5.1	77.4	81.6	73.2	18.5	21.3	15.7	-5.0	-3.7	-6.3
Tropical Latin America	96.6	104.7	88.6	32.9	38.4	27.5	2.1	4.0	0.4	186.7	211.3	163.4	8.4	9.4	7.3	7.7	8.7	6.7	91.9	99.3	84.5	31.2	36.3	26.2	1.0	2.2	-0.3
Western Europe	20.1	23.6	16.4	6.8	10.0	3.6	-4.0	-2.2	-6.0	554.5	630.9	489.5	12.7	14.5	11.2	9.4	10.7	8.3	19.6	23.5	15.7	5.4	8.8	2.0	-6.3	-4.4	-8.2
Western Sub-Saharan Africa	155.8	159.9	151.9	6.3	8.0	4.7	1.0	2.0	0.1	216.8	245.6	190.1	4.8	5.4	4.2	7.1	8.0	6.3	141.2	144.5	138.2	1.8	3.2	0.5	0.0	0.7	-0.7

Footnote: LBP: low back pain;

* Changes are the total mean values at 2019 compared with 1990

For each indicators, the 1st column was mean value and the 2nd and 3rd were upper uncertainty interval (UUI) and lower uncertainty interval (LUI) respectively.

Table S9 The number and prevalence of YLDs caused by LBP by 21 regions all over the world (2019 and the 30-year change)

men

Regions	2019									30-year change*								
	Number (×100,000)			All-age prevalence (per 100)			All-age prevalence (per 100)			Number (%)			All-age prevalence (%)			Age-standardized (%)		
Andean Latin America	1.9	2.5	1.3	0.6	0.8	0.4	0.6	0.8	0.4	109.4	120.0	99.4	25.0	31.3	19.0	-1.2	3.1	-5.2
Australasia	10.3	13.8	7.2	1.1	1.5	0.8	0.9	1.2	0.6	37.4	47.8	27.7	-3.3	4.0	-10.2	-14.6	-8.8	-20.3
Caribbean	268.4	359.2	189.0	0.6	0.8	0.4	0.6	0.8	0.4	55.5	61.7	49.6	16.3	21.0	11.9	-2.8	0.3	-5.6
Central Asia	27.3	36.2	19.6	0.7	0.9	0.5	0.8	1.0	0.5	59.9	65.6	53.6	17.0	21.1	12.3	-1.6	0.9	-4.2
Central Europe	6.7	8.9	4.7	1.4	2.0	1.0	1.1	1.5	0.8	10.6	14.2	7.2	19.7	23.5	16.0	-1.8	-0.2	-3.5
Central Latin America	9.9	13.3	7.0	0.5	0.7	0.4	0.5	0.7	0.4	109.6	118.6	101.4	39.0	45.1	33.6	5.8	8.2	3.5
Central Sub-Saharan Africa	1.6	2.2	1.1	0.5	0.6	0.3	0.7	1.0	0.5	153.7	166.0	140.6	6.7	11.9	1.2	1.0	5.3	-3.3
East Asia	11.8	15.9	8.3	0.6	0.8	0.4	0.5	0.7	0.4	16.8	22.5	10.8	-1.7	3.1	-6.8	-28.5	-26.3	-30.8
Eastern Europe	9.2	12.4	6.5	1.0	1.4	0.7	0.8	1.1	0.6	4.2	7.6	1.1	13.1	16.8	9.7	-4.2	-2.5	-5.8
Eastern Sub-Saharan Africa	3.1	4.2	2.2	0.5	0.7	0.4	0.8	1.1	0.6	135.2	140.0	130.6	8.5	10.7	6.4	1.4	3.0	-0.1
Global	35.6	47.9	25.1	0.7	0.9	0.5	0.7	0.9	0.5	46.4	49.8	43.0	1.6	4.0	-0.7	-16.1	-15.3	-16.8
High-income Asia Pacific	25.0	33.8	17.6	1.2	1.6	0.8	0.8	1.2	0.6	23.5	30.5	17.5	14.7	21.2	9.2	-9.2	-7.4	-11.0
High-income North America	2.1	2.8	1.5	1.5	2.0	1.1	1.2	1.6	0.9	30.3	38.1	23.0	-0.2	5.8	-5.8	-11.9	-7.2	-16.1
North Africa and Middle East	3.3	4.4	2.3	0.8	1.1	0.6	0.9	1.2	0.6	113.6	122.3	104.0	19.4	24.3	14.0	-8.8	-6.2	-11.5
Oceania	26.3	35.2	18.2	0.7	0.9	0.5	0.9	1.1	0.6	124.4	134.5	114.8	9.8	14.7	5.1	-0.1	3.8	-3.9
South Asia	1.5	1.9	1.0	0.4	0.5	0.3	0.4	0.6	0.3	49.9	54.7	44.9	-7.2	-4.2	-10.3	-23.9	-22.3	-25.8
Southeast Asia	8.1	10.9	5.7	0.8	1.0	0.5	0.8	1.0	0.5	87.1	94.1	80.4	28.9	33.7	24.2	-2.6	-1.0	-4.1
Southern Latin America	2.4	3.3	1.7	0.7	1.0	0.5	0.7	0.9	0.5	54.6	62.1	47.3	15.1	20.7	9.7	-1.0	3.7	-5.5

women

Regions	2019									30-year change*								
	Number (×100,000)			All-age prevalence (per 100)			Age-standardized (per 100)			Number (%)			All-age prevalence (%)			Age-standardized (%)		
Andean Latin America	2.3	3.1	1.6	0.7	1.0	0.5	0.7	1.0	0.5	113.1	124.8	102.0	20.3	25.6	15.5	0.3	4.7	-3.9
Australasia	1.9	2.6	1.4	1.3	1.8	0.9	1.1	1.4	0.7	51.2	62.5	41.3	6.3	9.6	3.1	-7.0	-1.0	-13.3
Caribbean	2.2	2.9	1.6	0.9	1.2	0.7	0.8	1.1	0.6	60.9	68.1	54.6	31.1	36.1	26.2	-1.4	2.0	-4.8
Central Asia	4.1	5.4	2.9	0.9	1.2	0.6	0.9	1.2	0.6	52.8	59.1	47.4	14.3	19.9	9.2	-0.9	1.9	-3.6
Central Europe	9.0	12.2	6.4	1.5	2.1	1.1	1.2	1.5	0.8	7.3	10.8	4.2	15.0	18.7	11.6	-2.9	-1.1	-4.6
Central Latin America	12.9	17.2	9.0	1.0	1.3	0.7	1.0	1.3	0.7	95.7	104.1	87.3	14.5	19.2	10.5	-3.1	-1.1	-4.9
Central Sub-Saharan Africa	3.3	4.5	2.3	0.5	0.7	0.4	0.8	1.0	0.5	142.4	153.7	131.3	28.7	35.7	22.0	-1.8	2.8	-6.0
East Asia	63.5	86.3	45.0	0.9	1.2	0.6	0.7	0.9	0.5	25.5	32.3	18.3	10.4	15.6	5.0	-28.7	-26.6	-30.6
Eastern Europe	18.6	24.9	13.2	1.7	2.2	1.2	1.2	1.6	0.8	7.0	9.5	4.4	4.6	12.4	-2.2	0.2	1.8	-1.4
Eastern Sub-Saharan Africa	8.4	11.1	5.8	0.4	0.5	0.3	0.6	0.9	0.5	127.1	131.8	122.4	33.2	39.0	27.6	-2.4	-0.7	-4.0
Global	368.5	494.5	260.7	1.0	1.3	0.7	0.9	1.2	0.6	47.2	51.2	43.4	27.1	32.6	21.7	-16.5	-15.6	-17.5
High-income Asia Pacific	18.0	24.2	12.7	1.9	2.6	1.3	1.3	1.8	0.9	16.8	22.7	12.0	-1.4	2.6	-5.1	-12.5	-10.8	-14.2
High-income North America	34.4	45.0	24.9	1.9	2.4	1.3	1.5	1.9	1.1	27.6	34.4	20.4	25.1	29.0	20.9	-10.7	-6.1	-15.1
North Africa and Middle East	22.7	30.3	15.9	0.8	1.0	0.5	0.8	1.1	0.6	117.5	124.2	110.1	3.2	8.8	-2.7	-2.7	-1.0	-4.5
Oceania	0.6	0.8	0.4	0.9	1.2	0.6	1.1	1.5	0.8	127.5	138.2	116.3	19.7	23.8	16.2	-0.2	4.0	-4.1
South Asia	63.6	84.8	45.1	0.7	1.0	0.5	0.8	1.0	0.5	65.3	72.0	59.1	-1.1	4.2	-6.7	-22.4	-20.4	-24.3
Southeast Asia	36.2	48.3	25.5	1.1	1.4	0.8	1.0	1.4	0.7	88.2	95.4	81.2	7.9	13.4	3.5	-0.6	1.3	-2.3
Southern Latin America	3.5	4.7	2.4	1.0	1.4	0.7	0.9	1.2	0.6	54.5	62.2	47.7	14.9	17.6	12.1	-1.3	3.2	-5.6

Both

Regions	2019									30-year change*								
	Number (×100,000)			All-age prevalence (per 100)			Age-standardized (per 100)			Number (%)			All-age prevalence (%)			Age-standardized (%)		
Andean Latin America	4.2	5.6	2.9	0.7	0.9	0.5	0.7	0.9	0.5	111.4	120.4	102.8	26.9	32.3	21.8	-0.3	2.8	-3.2
Australasia	3.5	4.8	2.5	1.2	1.6	0.9	1.0	1.3	0.7	44.7	53.6	35.8	1.0	7.2	-5.3	-10.7	-5.7	-15.9
Caribbean	3.6	4.9	2.6	0.8	1.0	0.5	0.7	1.0	0.5	58.7	64.3	53.4	18.7	22.8	14.7	-1.9	0.5	-4.4
Central Asia	7.3	9.8	5.1	0.8	1.0	0.5	0.8	1.1	0.6	55.9	61.4	50.9	15.4	19.5	11.8	-1.4	0.5	-3.1
Central Europe	17.1	23.1	12.1	1.5	2.0	1.1	1.1	1.5	0.8	8.8	12.1	6.0	17.2	20.7	14.1	-2.4	-1.1	-3.6
Central Latin America	19.5	26.0	13.7	0.8	1.0	0.5	0.8	1.0	0.5	100.2	108.1	91.9	31.4	36.6	26.0	0.3	1.8	-1.2
Central Sub-Saharan Africa	6.4	8.6	4.5	0.5	0.7	0.3	0.7	1.0	0.5	147.7	156.2	139.0	4.6	8.1	0.9	-0.3	2.8	-3.4
East Asia	108.9	147.5	77.2	0.7	1.0	0.5	0.6	0.8	0.4	21.7	27.7	15.2	1.3	6.3	-4.2	-28.4	-26.5	-30.2
Eastern Europe	28.5	38.2	20.2	1.4	1.8	1.0	1.0	1.4	0.7	6.0	8.6	3.4	14.4	17.1	11.6	-1.8	-0.6	-3.0
Eastern Sub-Saharan Africa	18.7	24.8	13.0	0.5	0.6	0.3	0.7	1.0	0.5	131.5	135.1	127.8	6.9	8.6	5.2	-0.4	0.7	-1.4
Global	636.9	851.9	450.0	0.8	1.1	0.6	0.8	1.0	0.5	46.9	50.5	43.3	1.5	4.1	-0.9	-16.3	-15.5	-17.1
High-income Asia Pacific	28.8	39.0	20.4	1.5	2.1	1.1	1.1	1.5	0.8	19.2	25.2	14.4	10.5	16.0	5.9	-12.0	-10.6	-13.3
High-income North America	61.7	81.2	44.5	1.7	2.2	1.2	1.4	1.8	1.0	28.8	35.9	21.8	-0.8	4.7	-6.2	-11.4	-6.9	-15.6
North Africa and Middle East	49.0	65.3	34.0	0.8	1.1	0.6	0.9	1.2	0.6	115.4	122.5	107.8	22.1	26.1	17.8	-6.0	-4.2	-7.7
Oceania	1.0	1.4	0.7	0.8	1.0	0.5	1.0	1.3	0.7	126.1	134.9	118.6	10.2	14.5	6.5	-0.2	2.9	-3.0
South Asia	99.2	132.5	70.3	0.5	0.7	0.4	0.6	0.8	0.4	59.4	64.7	54.1	-3.1	0.2	-6.3	-22.0	-20.4	-23.7
Southeast Asia	61.7	82.5	43.3	0.9	1.2	0.6	0.9	1.2	0.6	87.7	94.7	80.8	30.1	34.9	25.3	-1.5	-0.1	-2.9
Southern Latin America	5.9	8.0	4.1	0.9	1.2	0.6	0.8	1.1	0.6	54.5	60.2	49.2	14.7	18.9	10.7	-1.3	2.2	-4.4

Southern Sub-Saharan Africa	10.9	14.8	7.6	0.5	0.7	0.4	0.6	0.9	0.5	74.6	79.8	69.5	15.8	19.2	12.4	-6.0	-3.8	-8.0	2.3	3.1	1.6	0.6	0.8	0.4	0.6	0.9	0.4	77.9	83.9	72.6	6.9	8.6	5.0	-5.5	-3.2	-7.5	4.4	5.9	3.1	0.6	0.8	0.4	0.6	0.9	0.5	76.3	81.1	71.9	17.8	21.0	14.8	-5.6	-4.0	-7.1
Tropical Latin America	0.5	0.6	0.3	0.8	1.1	0.6	0.8	1.1	0.6	86.4	94.0	78.6	28.9	34.2	23.6	-0.1	1.7	-2.0	11.7	15.6	8.3	1.0	1.4	0.7	0.9	1.2	0.7	97.0	105.6	88.7	2.6	7.4	-2.1	2.7	4.8	0.7	21.0	27.9	14.8	0.9	1.2	0.7	0.9	1.1	0.6	92.2	99.9	84.2	31.4	36.7	26.0	1.5	2.9	0.0
Western Europe	45.4	61.7	31.8	1.2	1.6	0.8	0.9	1.2	0.6	18.0	22.9	13.5	3.0	7.3	-0.9	-8.3	-6.0	-10.6	36.8	49.3	26.0	1.7	2.2	1.2	1.2	1.6	0.9	19.4	23.2	15.8	1.4	4.1	-1.2	-4.0	-2.1	-6.2	61.7	83.2	43.4	1.4	1.9	1.0	1.1	1.4	0.7	18.9	22.8	15.2	4.8	8.3	1.5	-6.3	-4.4	-8.2
Western Sub-Saharan Africa	25.6	34.3	17.9	0.5	0.7	0.4	0.8	1.1	0.6	128.4	132.9	124.1	-2.0	-0.1	-3.9	-0.5	0.5	-1.7	12.6	16.9	8.9	0.5	0.7	0.4	0.8	1.1	0.6	157.2	161.5	152.8	5.0	7.2	2.8	1.4	2.5	0.3	24.5	32.8	17.2	0.5	0.7	0.4	0.8	1.1	0.6	142.5	145.8	139.1	2.3	3.7	0.9	0.5	1.3	-0.4

LBP: low back pain; YLDs: years lived with disability;

* Changes are the total mean values at 2019 compared with 1990

For each indicators, the 1st column was mean value and the 2nd and 3rd were upper uncertainty interval (UUI) and lower uncertainty interval (LUI) respectively.

Figure S1 The global map on the prevalent number of LBP and YLDs. (A) The prevalent number of LBP in 2019. (B) The prevalent number of YLDs caused by LBP in 2019. (C) The global map on the 30-year change of prevalent number of population with LBP (from 1990 to 2019). (D) The global map on the 30-year change of prevalent number of YLDs caused by LBP (from 1990 to 2019).(LBP: low back pain; YLDs: years lived with disability)

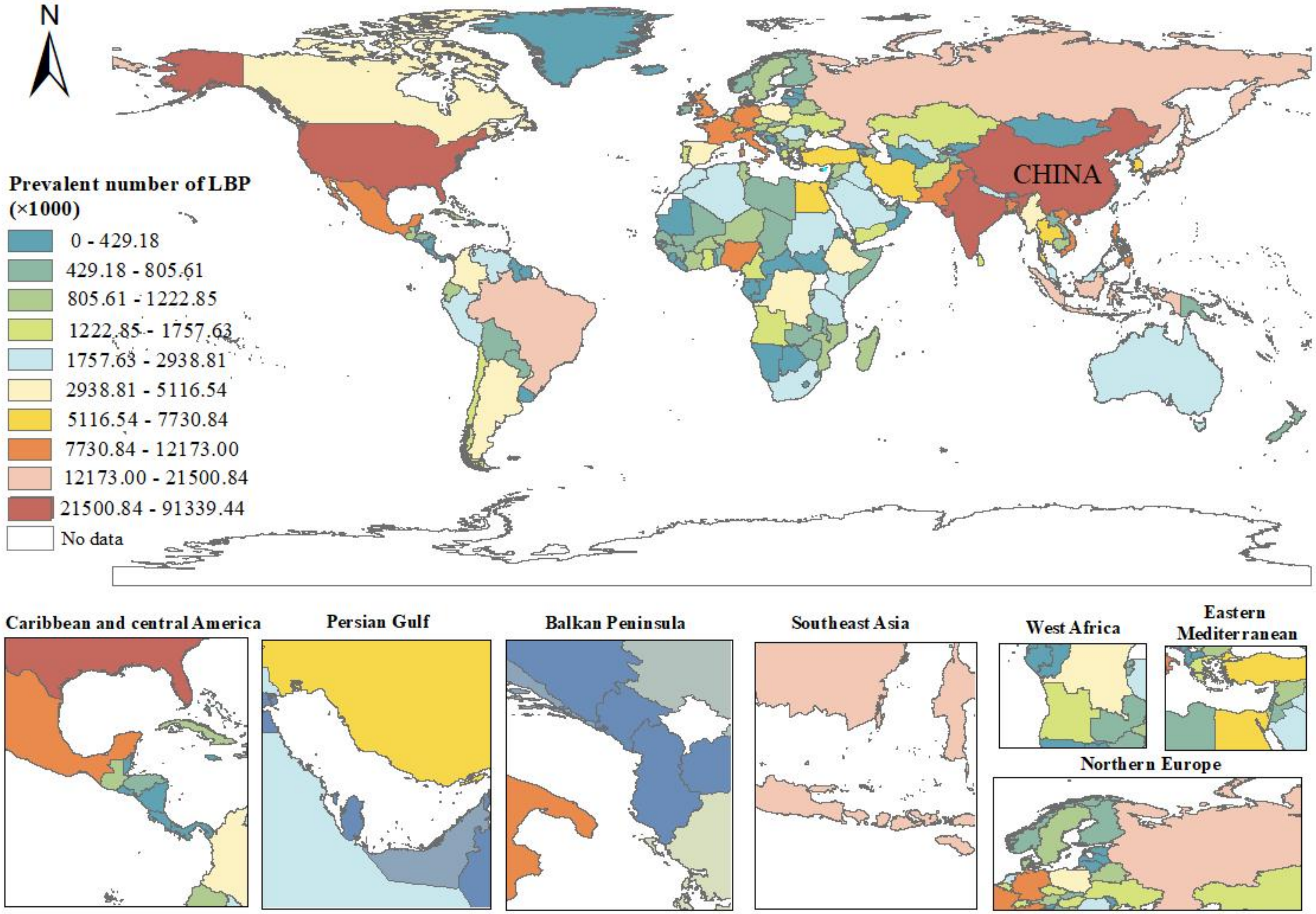

(A)

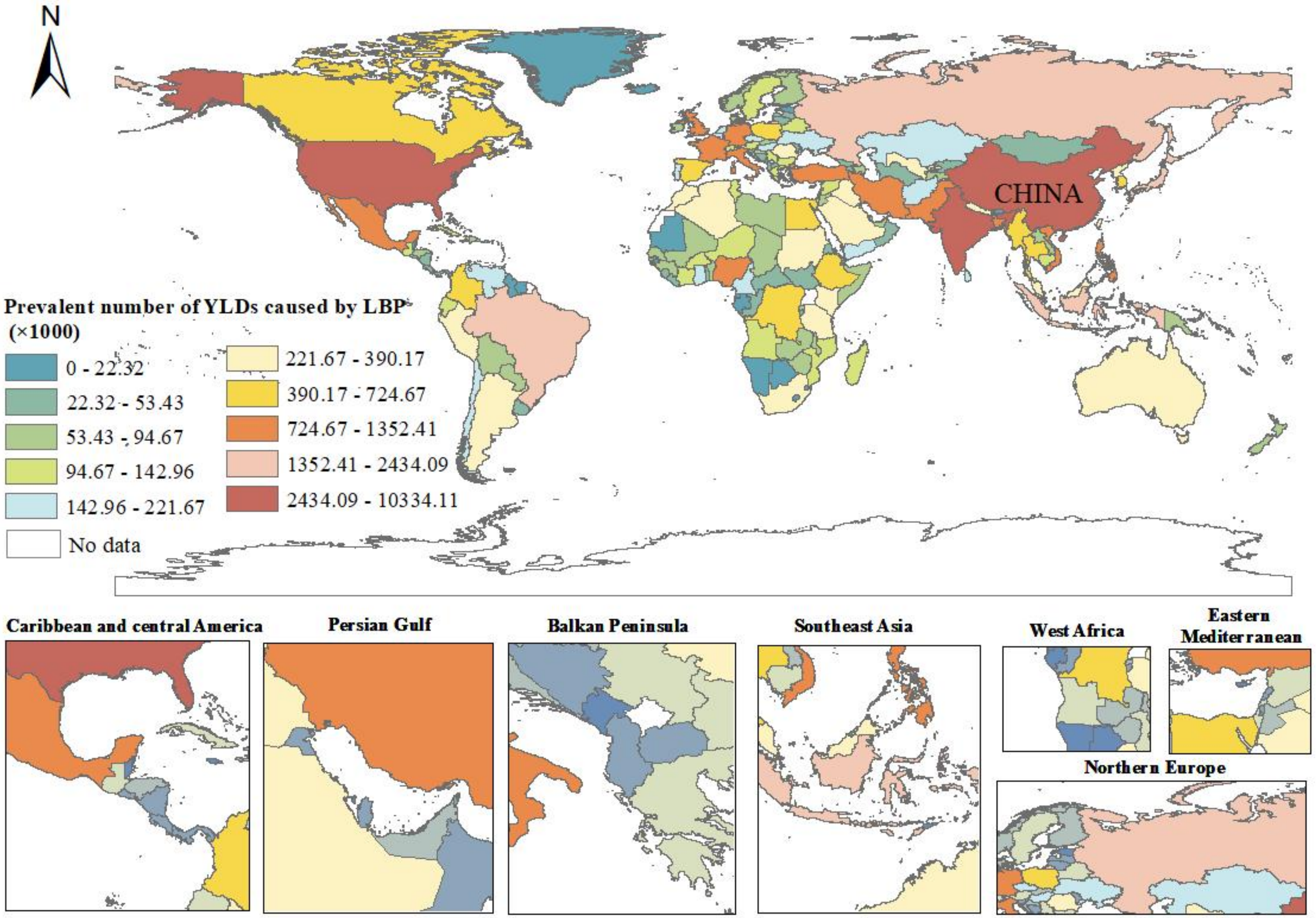
N
CHINA
Prevalent number of YLDs caused by LBP
(×1000)
0 - 22.32
22.32 - 53.43
53.43 - 94.67
94.67 - 142.96
142.96 - 221.67
221.67 - 390.17
390.17 - 724.67
724.67 - 1352.41
1352.41 - 2434.09
2434.09 - 10334.11
No data
Caribbean and central America
Persian Gulf
Balkan Peninsula
Southeast Asia
West Africa
Eastern Mediterranean
Northern Europe
(B)

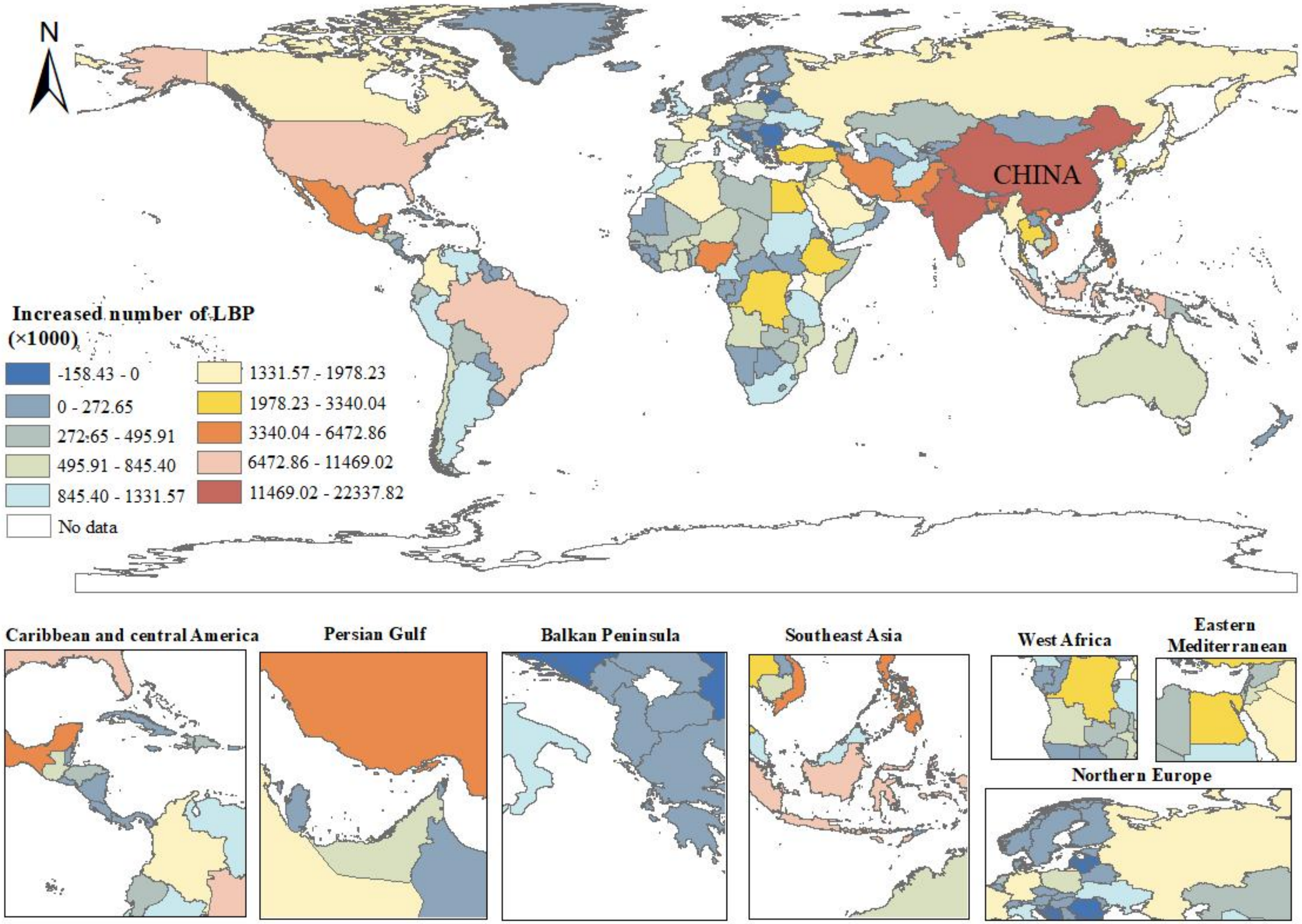
N
CHINA
Increased number of LBP (×1000)
-158.43 - 0
0 - 272.65
272.65 - 495.91
495.91 - 845.40
845.40 - 1331.57
No data
1331.57 - 1978.23
1978.23 - 3340.04
3340.04 - 6472.86
6472.86 - 11469.02
11469.02 - 22337.82
Caribbean and central America
Persian Gulf
Balkan Peninsula
Southeast Asia
West Africa
Eastern Mediterranean
Northern Europe

(C)

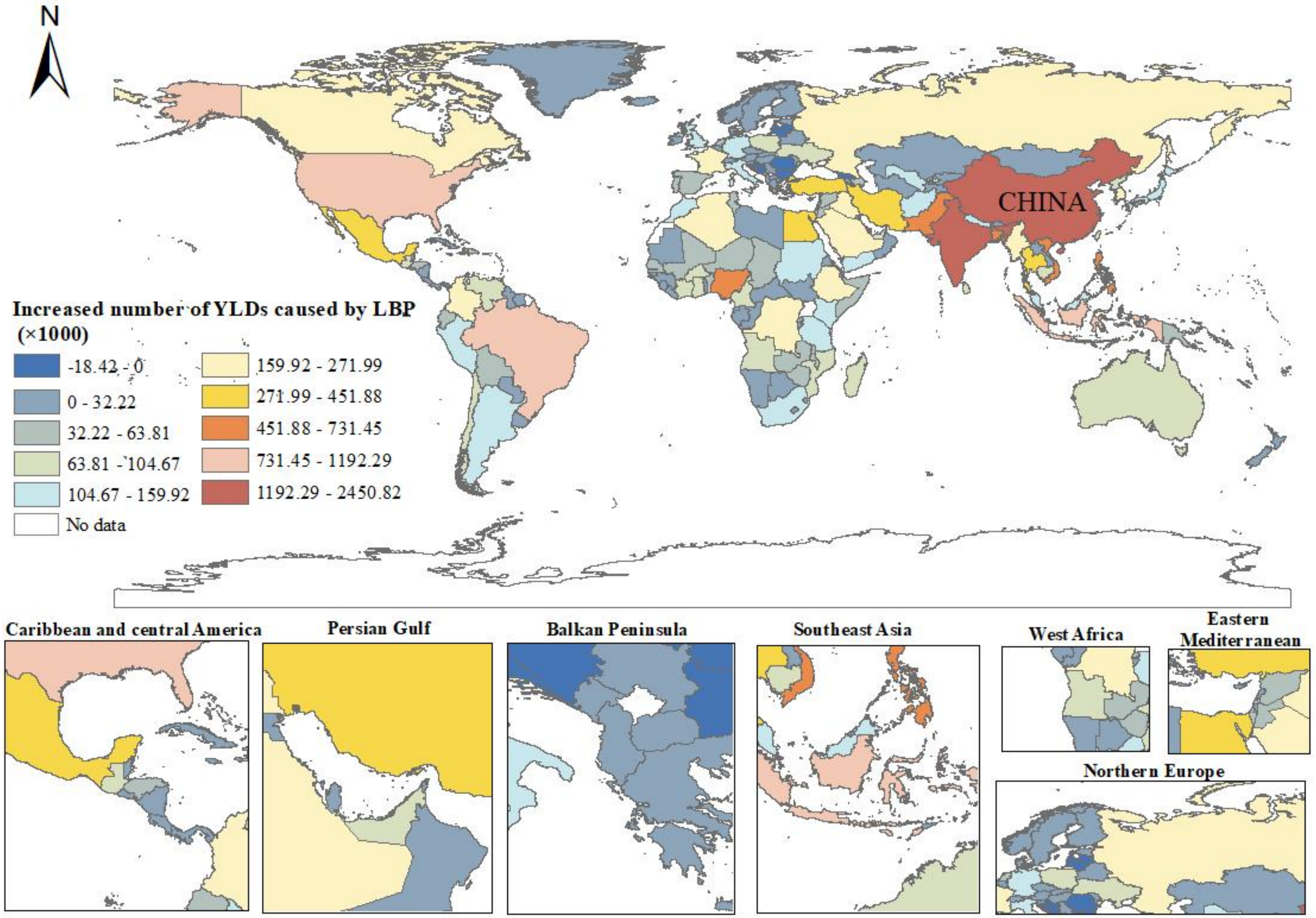
N
CHINA
Increased number of YLDs caused by LBP (×1000)
-18.42 - 0
0 - 32.22
32.22 - 63.81
63.81 - 104.67
104.67 - 159.92
159.92 - 271.99
271.99 - 451.88
451.88 - 731.45
731.45 - 1192.29
1192.29 - 2450.82
No data
Caribbean and central America
Persian Gulf
Balkan Peninsula
Southeast Asia
West Africa
Eastern Mediterranean
Northern Europe

(D)

Figure S2 (A)The rank of all cause of YLDs in 33 provinces/regions and in China (1990). (B) The rank of all cause of YLDs in 33 provinces/regions and in China (2019).

Both sexes, All ages, 1990, YLDs per 100,000

	**China**	Anhui	Beijing	Chongqing	Fujian	Gansu	Guangdong	Guangxi	Guizhou	Hainan	Hebei	Heilongjiang	Henan	Hubei	Inner Mongolia	Hunan	Jiangsu	Jiangxi	Jilin	Liaoning	Ningxia	Qinghai	Shaanxi	Shandong	Shanghai	Shanxi	Sichuan	Tianjin	Tibet	Xinjiang	Yunnan	Zhejiang	Hong Kong	Macao
Low back pain	1	1	1	1	1	1	1	1	1	1	1	1	1	1	1	1	1	1	1	1	1	1	1	1	1	1	1	1	1	1	1	1	1	1
Age-related hearing loss	2	3	2	2	2	4	2	2	2	2	2	4	3	4	4	4	2	2	3	2	4	4	4	3	2	2	2	2	2	4	2	3	2	2
Depressive disorders	3	2	6	3	3	2	4	4	4	3	4	3	2	2	3	2	4	4	4	4	5	3	2	2	7	4	4	6	5	2	4	2	3	4
Headache disorders	4	4	3	4	4	3	3	3	3	4	3	2	4	3	2	5	3	3	2	3	3	2	3	4	3	3	3	3	3	3	3	4	4	3
Anxiety disorders	5	5	11	7	5	5	12	5	5	6	7	6	5	6	5	3	8	5	5	11	2	7	5	5	17	6	7	7	6	5	6	7	11	7
Gynecological diseases	6	7	4	5	7	6	5	6	6	7	6	5	7	7	6	6	6	8	6	5	6	5	8	7	4	8	5	4	9	7	5	5	7	5
Neck pain	7	8	5	6	6	7	6	7	8	8	5	7	6	8	7	7	7	7	7	6	7	6	7	6	5	7	6	5	7	8	7	8	5	6
Dietary iron deficiency	8	6	20	8	10	8	13	8	7	5	8	10	8	5	11	8	5	6	9	10	8	8	6	9	6	5	8	14	4	6	21	6	19	14
COPD	9	9	14	9	8	10	7	9	11	11	14	13	10	9	10	9	9	11	15	13	10	9	14	8	10	11	9	12	11	13	10	10	13	9
Other musculoskeletal	10	12	9	13	12	15	11	12	18	16	11	11	11	11	12	11	11	17	11	9	14	13	9	11	8	12	12	10	17	12	20	13	6	8
Diabetes	11	10	12	11	13	14	10	18	13	9	13	9	14	17	13	12	15	14	10	8	16	17	12	15	12	9	14	9	18	9	17	15	17	22
Stroke	12	14	7	19	16	20	15	17	17	12	9	8	9	14	9	16	16	18	8	7	12	18	15	12	13	10	19	8	13	16	18	18	15	10
Oral disorders	13	11	15	15	14	13	17	13	12	15	12	15	13	13	14	14	13	12	12	14	11	12	10	13	18	13	10	13	14	14	16	12	8	12
Endo/metab/blood/immune	14	15	8	16	15	17	9	14	16	14	17	14	15	12	18	15	12	16	13	12	9	11	11	14	9	17	13	11	15	15	14	11	10	11
Schizophrenia	15	16	10	18	11	16	8	19	19	17	10	12	16	15	15	20	18	9	19	15	13	15	13	10	15	15	16	15	21	19	13	14	16	13
Drug use disorders	16	18	17	14	17	9	16	10	9	18	15	16	17	16	16	10	17	10	14	16	15	10	18	17	20	14	17	16	12	11	8	17	9	15
Blindness and vision loss	17	13	16	12	18	18	14	11	21	13	16	18	12	10	17	13	14	15	17	17	18	21	16	16	19	20	20	17	8	21	12	16	12	18
Falls	18	17	13	10	9	21	18	16	14	10	20	19	20	18	23	17	10	13	18	19	21	14	17	18	11	18	18	18	10	18	15	9	14	16
Osteoarthritis	19	21	18	17	23	19	22	20	15	21	19	21	18	19	20	18	19	21	21	18	23	19	19	19	21	16	15	19	25	17	19	20	18	19
Dermatitis	20	19	23	21	20	22	24	21	22	20	18	20	19	20	19	19	20	19	20	20	17	20	20	20	14	21	21	20	22	20	22	21	20	21
Alcohol use disorders	21	22	22	20	21	11	19	25	20	26	22	17	23	21	8	27	22	27	16	23	19	16	22	22	23	22	26	21	24	22	9	22	25	20
Scabies	22	23	24	23	24	23	25	22	24	22	21	22	21	22	21	21	23	20	23	22	20	22	23	21	26	23	23	23	26	23	24	23	23	23
Hemoglobinopathies	23	20	35	43	19	24	26	15	10	19	27	23	22	31	28	23	31	24	24	49	24	24	21	45	40	42	11	27	16	38	11	26	46	31
Congenital defects	24	24	30	22	25	25	21	23	26	23	24	25	24	26	24	22	27	23	25	25	22	23	34	24	32	26	24	26	23	24	26	25	33	32
Other mental disorders	25	26	25	24	29	28	28	28	32	29	25	26	25	27	26	25	25	29	26	24	29	28	25	25	25	25	25	24	31	29	30	24	22	24

(A)

Both sexes, Age-standardized, 2019, YLDs per 100,000

	**China**	Anhui	Beijing	Chongqing	Fujian	Gansu	Guangdong	Guangxi	Guizhou	Hainan	Hebei	Heilongjiang	Henan	Hubei	Hunan	Inner Mongolia	Jiangsu	Jiangxi	Jilin	Liaoning	Hong Kong	Macao	Ningxia	Qinghai	Shaanxi	Zhejiang	Yunnan	Shandong	Shanghai	Shanxi	Sichuan	Tianjin	Tibet	Xinjiang
Low back pain	1	1	1	1	1	1	1	1	1	1	1	2	1	2	1	1	1	1	2	1	1	1	1	1	2	4	2	2	3	1	1	1	2	1
Age-related hearing loss	2	2	2	2	2	2	2	2	2	2	2	3	2	1	2	2	2	2	1	2	2	2	2	2	1	1	1	1	1	2	2	2	1	2
Headache disorders	3	3	3	3	3	3	3	3	3	3	3	1	3	3	4	3	3	3	3	3	4	3	3	3	3	3	3	3	2	3	3	3	3	3
Depressive disorders	4	4	6	4	4	4	4	4	4	5	4	5	4	4	5	4	4	4	5	6	3	4	5	4	4	2	4	4	9	4	4	9	4	4
Neck pain	5	5	5	5	5	6	6	5	5	6	6	7	6	5	6	6	5	5	7	7	6	6	6	5	5	5	5	6	5	6	5	7	6	6
Other musculoskeletal	6	6	4	6	6	7	5	6	7	8	8	8	7	6	7	7	6	6	6	8	5	5	8	7	6	7	12	7	4	8	8	6	9	7
Diabetes	7	7	7	8	7	8	7	9	8	4	7	6	8	13	8	8	9	10	8	5	14	14	9	6	9	9	6	10	8	5	9	4	11	5
Stroke	8	8	8	13	12	10	9	7	6	7	5	4	5	10	9	5	7	13	4	4	15	9	7	10	8	16	8	8	13	7	13	5	7	10
Anxiety disorders	9	9	13	10	8	5	18	8	9	9	9	9	9	7	3	9	8	7	9	13	12	7	4	14	7	6	10	5	14	9	12	8	8	8
Gynecological diseases	10	10	9	9	14	12	10	11	10	12	10	11	11	9	10	12	10	14	10	9	8	8	10	9	14	10	7	14	6	13	7	11	17	12
Blindness and vision loss	11	11	14	12	13	11	11	10	15	10	11	10	10	8	12	10	14	9	11	14	9	16	12	13	12	15	9	15	18	14	17	13	5	14
COPD	12	13	21	7	16	9	14	12	11	15	15	14	14	11	11	11	13	11	16	17	18	15	13	8	16	17	11	12	22	16	6	17	10	9
Oral disorders	13	12	16	15	11	14	17	13	14	13	12	12	13	14	13	14	15	15	12	12	7	11	11	12	11	13	16	13	15	11	14	12	13	13
Neonatal disorders	14	14	11	14	10	15	15	16	16	11	13	17	12	12	16	15	12	16	13	10	20	12	14	16	13	12	19	11	12	12	15	16	15	16
Osteoarthritis	15	15	17	11	17	13	16	15	12	14	16	13	15	15	14	16	18	17	14	15	11	13	16	11	10	18	13	16	19	10	11	14	16	11
Endo/metab/blood/immune	16	16	10	17	18	16	8	14	18	16	19	15	16	17	15	18	16	18	15	11	10	10	15	15	15	11	15	17	10	18	16	10	18	17
Falls	17	18	12	16	9	22	12	17	13	17	20	20	18	16	17	22	11	12	22	20	13	18	18	17	17	8	14	18	7	19	10	19	12	19
Schizophrenia	18	17	15	18	15	17	13	18	19	18	14	16	17	18	21	17	20	8	19	16	16	17	17	18	18	19	17	9	16	15	18	15	20	18
Drug use disorders	19	20	18	19	20	21	19	19	17	19	18	19	20	20	18	20	19	20	17	18	17	19	21	22	20	20	20	19	20	20	19	18	24	15
Road injuries	20	21	19	21	19	23	21	21	20	20	17	23	23	19	20	19	17	19	23	22	21	21	19	19	19	14	22	21	17	17	20	22	21	22
Dermatitis	21	19	20	20	21	20	24	20	21	21	21	18	19	21	19	21	21	21	18	19	19	20	20	21	21	21	21	20	11	21	21	20	22	20
Alcohol use disorders	22	22	24	22	22	19	20	24	22	26	29	21	21	22	27	13	27	30	20	30	32	22	22	20	22	26	18	22	24	22	25	21	23	21
Alzheimer's disease	23	23	25	23	23	25	23	22	27	23	22	25	22	23	22	25	22	25	21	24	24	23	23	23	23	22	25	23	23	24	23	23	26	26
Scabies	24	24	23	24	24	24	25	23	23	22	23	24	24	24	23	23	23	22	24	23	22	24	24	24	24	23	24	24	25	23	24	25	25	25
Other mental disorders	25	25	26	26	25	28	26	25	25	24	24	26	26	25	25	26	24	23	26	25	25	25	27	25	25	24	26	26	26	25	27	26	28	27

(B)
